# Global analysis of lysine acetylation in strawberry leaves

**DOI:** 10.3389/fpls.2015.00739

**Published:** 2015-09-15

**Authors:** Xianping Fang, Wenyue Chen, Yun Zhao, Songlin Ruan, Hengmu Zhang, Chengqi Yan, Liang Jin, Lingling Cao, Jun Zhu, Huasheng Ma, Zhongyi Cheng

**Affiliations:** ^1^Institute of Biology, Hangzhou Academy of Agricultural SciencesHangzhou, China; ^2^Experiment Center, Hangzhou Academy of Agricultural SciencesHangzhou, China; ^3^Institute of Virology and Biotechnology, Zhejiang Academy of Agricultural SciencesHangzhou, China; ^4^Research and Development Center of Flower, Zhejiang Academy of Agricultural SciencesHangzhou, China; ^5^Jingjie PTM BiolabsHangzhou, China; ^6^Institute for Advanced Study of Translational Medicine, Tongji UniversityShanghai, China

**Keywords:** acetylome, bioinformatics, lysine acetylation, photosynthesis, proteomics, strawberry

## Abstract

Protein lysine acetylation is a reversible and dynamic post-translational modification. It plays an important role in regulating diverse cellular processes including chromatin dynamic, metabolic pathways, and transcription in both prokaryotes and eukaryotes. Although studies of lysine acetylome in plants have been reported, the throughput was not high enough, hindering the deep understanding of lysine acetylation in plant physiology and pathology. In this study, taking advantages of anti-acetyllysine-based enrichment and high-sensitive-mass spectrometer, we applied an integrated proteomic approach to comprehensively investigate lysine acetylome in strawberry. In total, we identified 1392 acetylation sites in 684 proteins, representing the largest dataset of acetylome in plants to date. To reveal the functional impacts of lysine acetylation in strawberry, intensive bioinformatic analysis was performed. The results significantly expanded our current understanding of plant acetylome and demonstrated that lysine acetylation is involved in multiple cellular metabolism and cellular processes. More interestingly, nearly 50% of all acetylated proteins identified in this work were localized in chloroplast and the vital role of lysine acetylation in photosynthesis was also revealed. Taken together, this study not only established the most extensive lysine acetylome in plants to date, but also systematically suggests the significant and unique roles of lysine acetylation in plants.

## Introduction

Protein post-translational modifications (PTMs) play important roles in cell signaling, protein–protein interactions, protein stability as well as activation/deactivation of enzymatic activity (Mann and Jensen, 2003; Jensen, 2004; Walsh et al., 2005; Witze et al., 2007; Meinnel and Giglione, 2008). As of today, more than 400 PTMs have been identified, and among them, lysine-N-epsilon-acetylation (lysine acetylation) is a highly conserved PTM occurring in a large number of proteins of prokaryotes and eukaryotes (Mischerikow and Heck, 2011). Lysine acetylation of proteins is a reversible and dynamic modification regulated by counteracting lysine acetyltransferases and lysine deacetylases (Liu et al., 2014). Since the first reveal of lysine acetylome in mammalian cells (Kim et al., 2006), acetylome in eukaryotes has been reported and the mechanistic studies showed that lysine acetylation impacted various cellular processes including transcriptional regulation and metabolic stability (Weinert et al., 2011; Bharathi et al., 2013; Rardin et al., 2013; Still et al., 2013). Besides, more and more studies indicated that lysine acetylation extensively occurred in prokaryotes, and functional annotation showed lysine acetylation functions in metabolic pathways, stress response, and enzymatic activity regulation in bacteria (Zhang et al., 2009, 2013; Thao and Escalante-Semerena, 2011).

Despite the popular studies and high throughput datasets of lysine acetylation in mammalian cells and bacteria, the progress of lysine acetylome in plants is relative limited. More recently, with the advantages of antibody-based affinity enrichment and high sensitive MS-based analysis, lysine acetylome in plants has been gradually studied (Rao et al., 2014a,b). Recent plant lysine acetylome studies have identified 35 lysine acetylation sites in 31 proteins in *Solanum tuberosum* (Xing and Poirier, 2012), 66 lysine acetylation sites in 44 proteins in *Oryza sativa* (Nallamilli et al., 2014), 138 lysine acetylation sites in 97 proteins *Vitis vinifera* (Melo-Braga et al., 2012), and 190 lysine acetylation sites in 121 proteins in *Glycine max* (Smith-Hammond et al., 2014a). Two studies on *A. thaliana* detected 64 acetylated sites in 57 proteins and 91 acetylated sites in 74 proteins, respectively (Finkemeier et al., 2011; Wu et al., 2011). The latest mitochondrial acetylome studies have identified 243 lysine acetylation sites in 120 proteins in *A. thaliana* (König et al., 2014), and 664 lysine acetylation sites in 358 proteins in *Pisum sativum* (Smith-Hammond et al., 2014b). Nevertheless, compared with thousands of lysine acetylation events and well revealed multiple functions of lysine acetylation in mammalian cells and bacteria, the extant number of lysine acetylated proteins is small in plants and only five plant species have been examined. Great challenges still remain to elaborate lysine acetylome in plants, hindering the deep understanding of lysine acetylation in plant physiology and pathology.

In this work, using strawberry as the material we adopted an integrated system by taking the combined advantages of anti-acetyllysine-based enrichment, high sensitive mass spectrometry, and intensive bioinformatic analysis to comprehensively characterize lysine acetylome in plants. By using the integrated approach, we successfully identified 1392 acetylation sites in 684 proteins in strawberry. To our best knowledge, it is the first systematic investigation of lysine acetylome in strawberry and also the largest dataset of acetylome in plants to date. With the help of advanced bioinformatic tools, a number of important biological processes, and functions highly related with lysine acetylation were revealed and lysine acetylation was demonstrated to target photosynthesis. As a consequence, this study not only greatly expanded our knowledge to plant acetylome, but also shed light on the functional annotation of lysine acetylation in plant physiology.

## Materials and methods

### Experiment design and workflow

**Figure 1:**
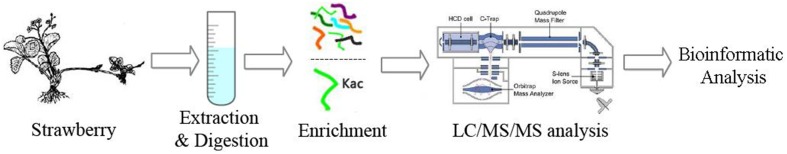
**The workflow of integrated strategy for global mapping of lysine acetylation in strawberry leaves**.

The purpose of this study was to perform the global identification of lysine acetylome in strawberry. The experimental design and workflow were outlined in Figure 1. Briefly, the protocol contained four key steps: (I) Strawberry leaves collection, protein extraction, and trypsin digestion, (II) Affinity enrichment of lysine acetylated peptides, (III) Analysis of lysine acetylated peptides by using nano-LC-MS/MS, (IV) Bioinformatics analysis for systematic interpretation of the identified lysine acetylated proteins. Three biological replicates were analyzed with LC-MS/MS.

### Plant materials

The *Fragaria ananassa* cultivar Hongjia was obtained from Hangzhou Academy of Agricultural Sciences, Zhejiang, China. Plant cultivation was carried as described previously (Fang et al., 2012). Briefly, the plants were grown in a tunnel greenhouse with a 10-h light/14-h dark cycle, a 30°C-day/26°C-night temperature cycle, 150 μmol m^−2^ s^−1^ light intensity, and a relative humidity of 60%. The strawberry leaves were picked from different greenhouses and frozen in liquid nitrogen, and stored at −80°C prior to protein extraction.

### Western blotting

To gain an initial overview of the extent of lysine acetylation on plant cell proteins, we performed the western-blot analysis using a commercially available antibody against acetyl-Lysine residues as previously descripted (Nallamilli et al., 2014). Proteins were separated by SDS-PAGE, transferred to a PVDF (Millipore) membrane and probed using acetylated lysine antibody in a 1:1000 dilution (PTM Biolabs, Hangzhou, China). Secondary anti-horseradish peroxidase antibody (HuaAn Biotechnology, Hangzhou, China) was used in a 1:10,000 dilution.

### Proteomic analysis

#### Protein extraction and tryptic digestion

The strawberry leaves were first ground in liquid nitrogen using a mortar and pestle. The powder was then transferred to a 50 mL centrifuge tube and precipitated with cold 10% TCA/acetone supplemented with 50 mM DTT, 0.1% Protease Inhibitor Cocktail Set VI and PVPP powder for 2 h at −20°C. After centrifugation at 20,000 g at 4°C for 10 min, the supernatant was discarded. The remaining precipitate was washed with cold acetone supplemented with 50 mM DTT, 1 mM PMSF for three times. After air drying, the precipitate was re-suspended in lysis buffer (8 M urea, 2 mM EDTA, 10 mM DTT, and 0.1% Protease Inhibitor Cocktail Set VI). The sample was sonicated three times on ice using a high intensity ultrasonic processor (Scientz). The remaining debris were removed by centrifugation at 20,000 g at 4°C for 10 min. The supernatant was transferred to a new tube, and proteins were reduced with 10 mM DTT for 1 h at 56°C and alkylated with 55 mM iodoacetamide for 45 min at room temperature in darkness. Proteins were precipitated with 3 volumes of pre-chilled acetone for 30 min at -20°C. After centrifugation, the pellet was then dissolved in 0.5 M TEAB and sonicated for 5 min. Following a second centrifugation step as above, the supernatant was collected. Protein content was determined with 2-D Quant kit (GE Healthcare) according to the manufacturer's instructions. Approximately 10 mg protein for each replicate was digested with trypsin (Promega) overnight at 37°C in a 1:50 trypsin-to-protein mass ratio.

#### Affinity enrichment of lysine-acetylated peptides

Affinity enrichment of lysine acetylated peptides was performed as described previously (Wu et al., 2013). Briefly, to enrich Kac peptides, tryptic peptides were dissolved in NETN buffer (100 mM NaCl, 1 mM EDTA, 50 mM Tris-HCl, 0.5% NP-40, pH 8.0) and incubated with pre-washed anti-acetyllysine agarose beads (PTM Biolabs, Hangzhou, China) at 4°C overnight with gentle shaking. The beads were carefully washed four times with NETN buffer and twice with ddH_2_O. The bound peptides were eluted from the beads with 0.1% trifluoroacetic acid (TFA). The eluted fractions were combined and vacuum-dried in the SpeedVac. The resulting peptides were cleaned with C18 ZipTips (Millipore) according to the manufacturer's instructions, followed by LC-MS/MS.

#### LC-MS/MS analysis

Peptides were dissolved in 0.1% FA, directly loaded onto a reversed-phase column and eluted with a linear gradient of 5–20% solvent B (0.1% FA in 98% ACN) for 30 min and 20–35% solvent B for 10 min at a constant flow rate of 300 nl/min on an EASY-nLC 1000 UPLC system. The resulting peptides were analyzed by Q Exactive™ hybrid quadrupole-Orbitrap mass spectrometer (ThermoFisher Scientific).

The peptides were subjected to NSI source followed by tandem mass spectrometry (MS/MS) in Q Exactive coupled online to the UPLC. Intact peptides were detected in the Orbitrap at a resolution of 70,000. Peptides were selected for MS/MS using 25% normalized collisional energy (NCE) with 12% stepped NCE and the fragments produced from low, medium, and high collisional energy were combined and detected simultaneously (Diedrich et al., 2013) Ion fragments were detected in the Orbitrap at a resolution of 17,500. A data-dependent procedure that alternated between one MS scan followed by 20 MS/MS scans was applied for the top 20 precursor ions above a threshold ion count of 3E4 in the MS survey scan with 15.0 s dynamic exclusion. The electrospray voltage applied was 2.0 kV. Automatic gain control (AGC) was used to prevent overfilling of the ion trap; 1E5 ions were accumulated for generation of MS/MS spectra. For MS scans, the m/z scan range was 350–1600.

#### Database search

The resulting MS/MS data were searched by using MaxQuant with integrated Andromeda search engine (v.1.4.0.5). Tandem mass spectra were searched against strawberry protein database (45,377 sequences, ftp://ftp.kazusa.or.jp/pub/strawberry/pep/FANhybrid_r1.2.pep) (Shulaev et al., 2011) concatenated with reverse decoy database and protein sequences of common contaminants. Trypsin/P was specified as cleavage enzyme allowing up to three missing cleavages, four modifications per peptide, and five charges. Mass error was set to 6 ppm for precursor ions and 0.02 Da for fragment ions. Carbamido methylation on Cys was specified as fixed modification and oxidation on Met, acetylation on Lys and acetylation on protein N-terminal were specified as variable modifications. False discovery rate (FDR) thresholds for protein, peptide, and modification site were specified at 0.01. Minimum peptide length was set at 7 Lysine acetylation site identifications with localization probability less than 0.75 or from reverse and contaminant protein sequences were removed.

### Bioinformatic analysis

#### Protein annotation, classification, and subcellular location prediction

Gene Ontology (GO) annotation proteome was derived from the UniProt-GOA database (http://www.ebi.ac.uk/GOA/). Firstly, lysine acetylated protein ID was converted to UniProt ID and then mapped to GO ID by protein ID. If identified lysine acetylation substrates were not annotated by UniProt-GOA database, the InterProScan soft then could be used to annotate protein's GO function based on protein sequence alignment method (Dimmer et al., 2012). Then lysine acetylation proteins were further classified by Gene Ontology annotation based on the categories of biological process and molecular function. Kyoto Encyclopedia of Genes and Genomes (KEGG) database was used to annotate protein pathway. Firstly, KEGG online service tools KAAS was used to annotate protein's KEGG database description (Moriya et al., 2007). Then annotation result was mapped on the KEGG pathway database using KEGG online service tools KEGG mapper. WoLF PSORT was used for subcellular localization predication (Horton et al., 2007).

#### Functional enrichment analysis

GO function enrichment analysis on three ontologies (biological process, cellular component, and molecular function) and KEGG pathway enrichment analyses were performed to gain further insights into the involved function and pathways of the acetylated proteins. Fisher's exact test was used to test for enrichment or depletion (right-tailed test) of specific annotation terms among members of resulting protein clusters. Derived *p*-values were further adjusted to address multiple hypotheses testing by the method proposed by Benjamini and Hochberg. Any terms having adjusted *p*-values below 0.05 in any of the clusters were treated as significant (Huang da et al., 2009).

#### Enrichment-based clustering analysis

All the lysine acetylation substrates categories (biological process, cellular component, and molecular function) were obtained after enrichment collated along with their *P*-values, and were then filtered for those categories which were at least enriched in one of the clusters with *P* < 0.05. This filtered *P*-value matrix was transformed by the function *x* = −log10 (*P*-value). Finally these *x*-values were z-transformed for each category. These z scores were then clustered by one-way hierarchical clustering (Euclidean distance, average linkage clustering) in Genesis. Cluster membership were visualized by a heat map using the “heatmap.2” function from the “gplots” R-package (Wu et al., 2013).

#### Analysis of sequence model around acetylated lysine

Software motif-x was used to analyze the model of sequences constituted with amino acids in specific positions of acetyl-21-mers (10 amino acids upstream and downstream of the acetylation site) in all protein sequences. All the database protein sequences were used as background database parameter, other parameters with default (Pan et al., 2014).

#### Protein-protein interaction network analysis

We analyzed protein-protein interaction for identified proteins using Cytoscape software (Shannon et al., 2003). Protein-protein interaction network was obtained from STRING database (Szklarczyk et al., 2011). STRING defines a metric called “confidence score” to define interaction confidence; we selected all interactions that had a confidence score ≥0.7 (high confidence). Interaction network form STRING was visualized in Cytoscape. A novel graph theoretical clustering algorithm, “Molecular Complex Detection” (MCODE), was used to detect densely connected regions in large protein-protein interaction networks that may represent molecular complexes. MCODE is part of the plug-in tool kit of the network analysis and visualization software Cytoscape.

## Results and discussion

### Identification of lysine acetylation in strawberry

As a kind of non-model plant, the genome information of cultivated strawberry, notably for the octoploid species and hybrids as the *Fragaria ananassa* cultivar presently used, is not complete, which complicates proteomics research, and functional analyses. But strawberry is a very important cash crop, and it is very meaningful to explore the physiological function of strawberries using proteomic methods. Previously, we investigated the global proteome of strawberry leaves toward *colletotrichum fragariae* infection and found a number of proteins (Fang et al., 2012). However, proteomic studies of PTMs such as lysine acetylome were not explored yet in strawberry. Recent studies show that protein acetylation may play an important role in a wide variety of physiological processes involving signal transduction, glucose responses, and pathogen-resistance pathway (Weinert et al., 2011; Bharathi et al., 2013; Rardin et al., 2013; Still et al., 2013). So, in this research we are willing to study protein PTM of the strawberry leaves, which will be beneficial to further explore the physiological functions and disease-resistance mechanism of strawberries.

**Figure 2:**
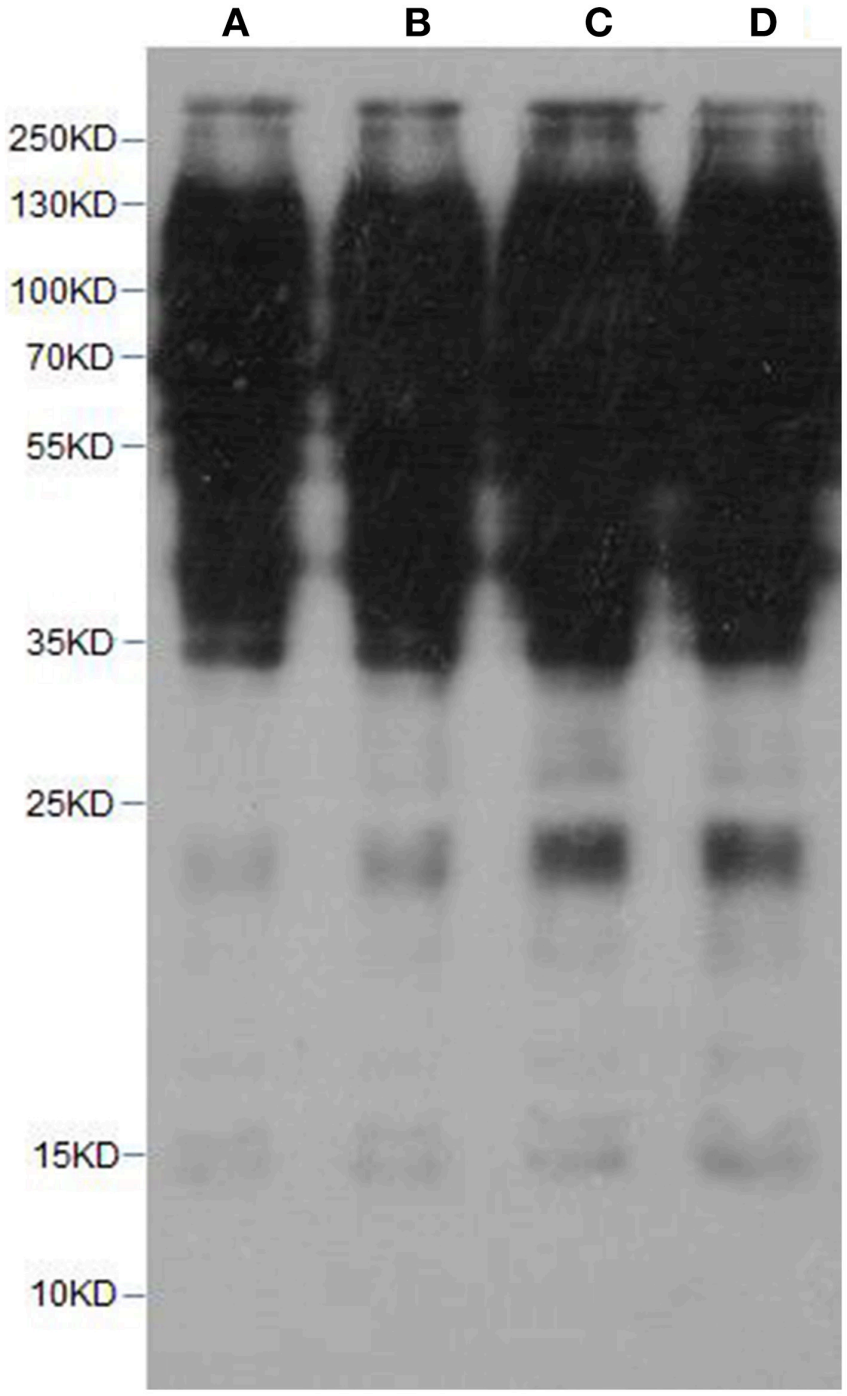
**Western blotting of strawberry leaves proteins with pan anti-acetylation antibody**. **(A,B)** lanes, 10 ug of crude protein from strawberry leaves; **(C,D)** lanes, 20 ug of crude protein from strawberry leaves.

In this work, lysine acetylation in strawberry was comprehensively studied by using high-throughput proteomic techniques. First of all, to roughly detect lysine acetylome in strawberry, proteins prepared from the strawberry leaves were examined by western blotting with lysine acetylation-specific pan antibodies as described previously (Nallamilli et al., 2014). As a result, multiple major protein bands with molecular weight higher than histones were successfully detected (Figure 2), indicating that lysine acetylation not only happens to histones, but also occurs in non-histone proteins, which is consistent with previous reports (Spange et al., 2009; Nallamilli et al., 2014).

Lysine acetylomes in plants were previously investigated by a number of researchers. Thus, Smith-Hammond et al. identified 664 acetylation sites in 358 proteins in pea seedling mitochondria (Smith-Hammond et al., 2014b). Moreover, in *Arabidopsis thaliana*, Wu et al. identified 64 acetylated sites in 57 proteins while Finkemeier et al. detected 91 acetylated sites in 74 proteins (Finkemeier et al., 2011; Wu et al., 2011). Nallmilli et al. recently reported the identification of 60 lysine acetylated sites in 44 proteins in rice (Nallamilli et al., 2014). In our present work, lysine acetylation identification was carried out on proteome level. A total of 1392 unique lysine acetylation sites in 684 proteins from three biological replicates were successfully identified as shown in Supplementary Table 1 and Supplementary Figure S1. The mass spectrometry proteomics data have been deposited to the ProteomeXchange Consortium (Vizcaíno et al., 2014) via the PRIDE partner repository with the dataset identifier PXD002758. To the best of our knowledge, this is the first time that more than 1000 lysine acetylation sites were identified in a single analysis and also the largest dataset of lysine acetylation in plants to date. In this study, the expansion of lysine acetylation may be attributed to the different intrinsic acetylation level of the proteins among various plant species. The great performance of pan anti-acetyllysine antibody-based enrichment technique also greatly promoted the increase of the identified lysine acetylated sites. The enlarged lysine acetylome in the present study may help reveal the important role of lysine acetylation in plant growth and development as well as plant physiology.

### Functional characterization of lysine acetylated proteins in strawberry

**Figure 3:**
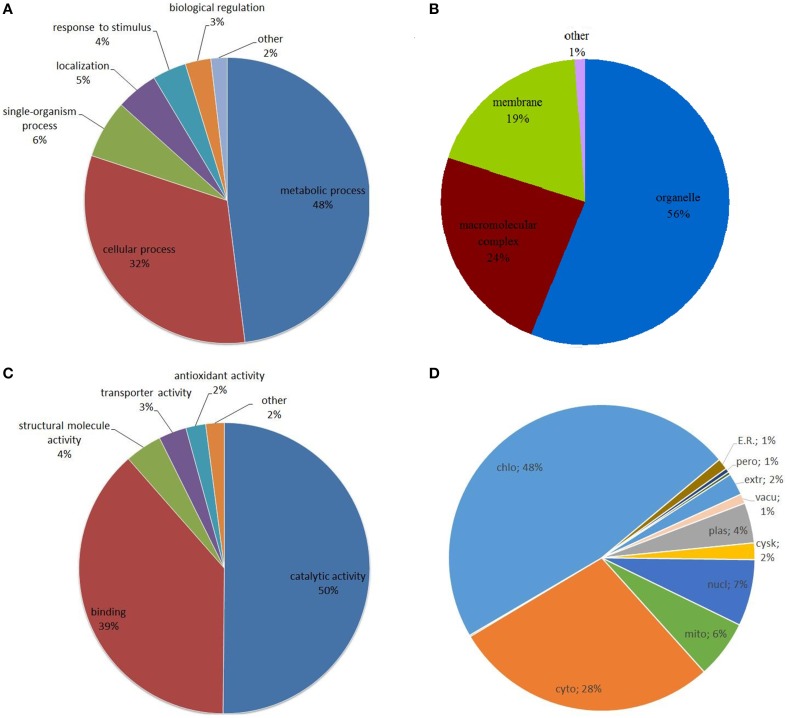
**Distribution of the identified lysine acetylated proteins in Gene Ontology categories of biological process (A), cellular component (B), and molecular function (C), and subcellular location prediction of all the lysine acetylated proteins (D)**.

To gain a better understanding of the distribution and function of the lysine acetylated proteins identified in this work, Gene Ontology (GO) function classification analysis was performed. First of all, an analysis of the biological process showed that metabolic process and cellular process related proteins were the major acetylated proteins in strawberry, which accounted for 53.5% (366) and 30.4% (208) of all the lysine acetylated proteins, respectively (Figure 3A). Previous studies had demonstrated many metabolism and cellular process related proteins can be acetylated in lysine sites and this reversible lysine acetylation is emerging as a major regulatory mechanism in cellular metabolism and cellular homoeostasis in bacteria, nimal and human (Zhao et al., 2010; Scott, 2012; Xiong and Guan, 2012). Here we speculate lysine acetylation may also play important role in plant metabolism and cellular regulation.

For the ontology of cellular component, the acetylated proteins belonged to various cellular components and the proportion of organelle, macromolecular complex and membrane related proteins were 55.66% (380), 24.53% (167), and 18.92% (129), respectively (Figure 3B).

For the molecular function ontology, it was shown that proteins associated with catalytic activity and binding corresponded to nearly 90% (609) of all lysine acetylated proteins as shown in Figure 3C. It is well-known that enzymes can regulate biochemical reactions in living organisms through their catalytic activity and specific binding to certain substrates (Porter et al., 2004). A recent review showed that reversible lysine acetylation could regulate metabolic enzymes on three principle levels: the amount of enzyme, the catalytic activity and the accessibility of substrates (Xiong and Guan, 2012). We infer the majority of acetylated proteins in the present study were enzyme related proteins.

Analysis of proteins distribution within the subcellular localization indicated that the majority of identified Kac proteins were predicted to localize in chloroplast (324, 47.5%) (Figure 3D), suggesting the important roles of lysine acetylation in this compartment. Moreover, a number of proteins were also localized in cytoplasm (192, 28.1%) and very few proteins were predicted to localize in other components such as nucleus (48, 7.0%), mitochondria (42, 6.1%), or plasma membrane (29, 4.2%) (Figure 3D). This data were then compared with those obtained for other plant systems such as Arabidopsis and rice. In Arabidopsis, the top three subcellular components are intracellular, cytoplasm and chloroplast, accounting for 23.1, 15.7, and 14.4% of all acetylated proteins, respectively (Wu et al., 2011). In rice, the top three subcellular components are nucleus, organelle and plastid, accounting for 17.2, 13.7, and 10.3% of all acetylated proteins, respectively (Nallamilli et al., 2014). However, in our data, the top three subcellular components are chloroplast, cytoplasmic components and nucleus, accounting for 48, 28, and 7% of all acetylated proteins. It is noted that in our data the percentage of acetylated proteins in chloroplast (48%) is much higher than in the previously published data (Wu et al., 2011; Nallamilli et al., 2014).

**Figure 4:**
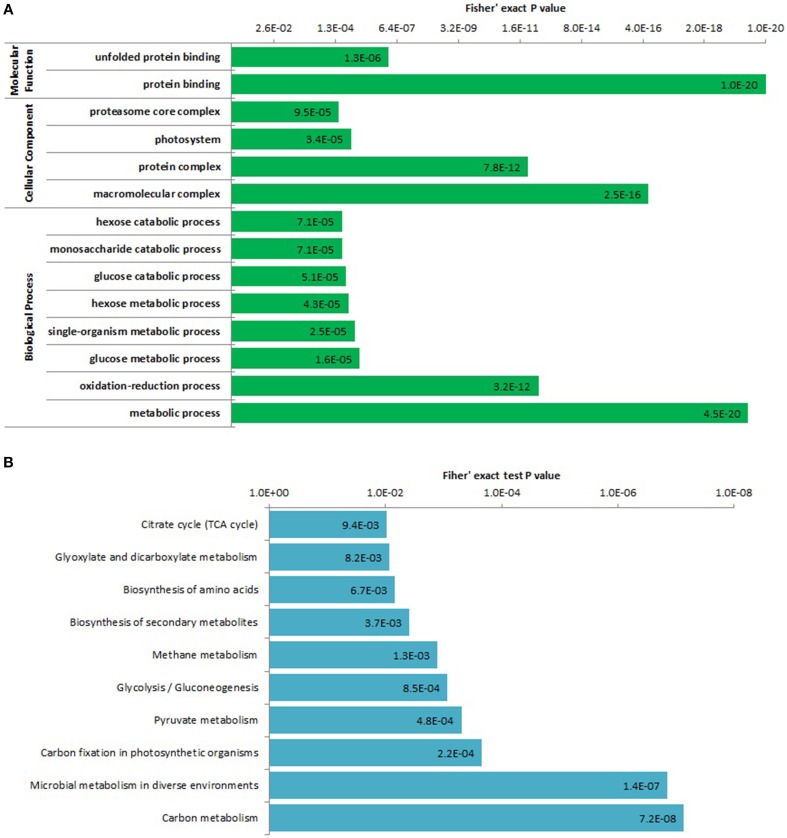
**Enrichment analysis of all the identified lysine acetylated proteins based on GO annotation (A) and KEGG pathway (B)**.

To determine which types of proteins are preferred targets for lysine acetylation, we performed the enrichment analysis of all the identified lysine acetylated proteins and the top significantly enriched GO terms and pathways were shown in Figure 4. In biological process ontology, several glycometabolism related terms were significantly enriched, such as GO terms metabolic process, glucose metabolic process, hexose metabolic process, glucose catabolic process, monosaccharide catabolic process, and hexose catabolic process. It is well-known that glycometabolism including glycolysis, citrate cycle, and gluconeogenesis can play important roles in plant life activities (Plaxton, 1996). Acetylation of glycometabolism and even general carbon metabolism related proteins (mainly enzymes) may be an important regulation mechanism of plant life activities such as plant growth and development. Previous studies had found enzymes of carbon metabolism such as glycolysis and the citrate cycle can be acetylated in *Escherichia coli, Saccharomyces cerevisiae, Drosophila*, human and rat (Zhang et al., 2009; Zhao et al., 2010; Weinert et al., 2011; Fritz et al., 2012; Henriksen et al., 2012). The acetylation of carbon metabolism related enzymes may be a conserved regulation pattern of life activity in organisms. We also obtained many significantly enriched carbon metabolism related pathways by KEGG pathway enrichment analysis (Figure 4B and Supplementary Package 1), such as pathway carbon metabolism, carbon fixation in photosynthetic organisms, pyruvate metabolism, glycolysis/gluconeogenesis, glyoxylate, and dicarboxylate metabolism, citrate cycle.

Two protein binding related terms were obtained by enrichment analysis on the category of molecular function, which was consistent with the GO classification results. For the category of cellular component, we found that the enriched terms were all involved in protein complex (Figure 4). We infer lysine acetylation probably participated in assembling and/or disassembling of macromolecular complexes in strawberry and subsequently affected protein function. However, further biochemical experiments are needed to verify this assumption.

### Functional enrichment based clustering analysis

**Figure 5:**
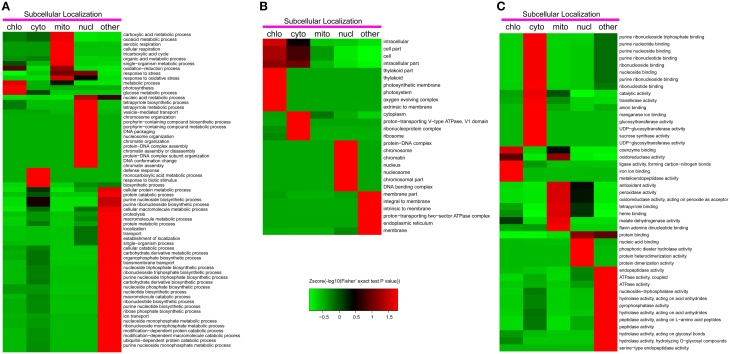
**Functional clustering analysis of all the identified lysine acetylated proteins in Gene Ontology categories of biological process (A), cellular component (B) and molecular function (C)**.

To reveal the preferential target substrates of lysine acetylation in different organelles, we carried out the functional enrichment based clustering analysis (Figure 5).

As shown in Figure 5A, in the biological process category, photosynthesis was significantly enriched in chloroplast. This result suggested that photosynthesis related proteins were the preferential target substrates of lysine acetylation in chloroplast. In agreement with this observation, the analysis by cellular component indicated that photosynthesis related GO terms including photosynthetic membrane, photosystem, oxygen evolving complex, and thylakoid were significantly enriched in chloroplast (Figure 5B). In mitochondrion, it was observed that glycometabolism and general carbon metabolism related terms such as carboxylic acid metabolic process, oxoacid metabolic process, tricarboxylic acid cycle, cellular respiration, and organic acid metabolic process were significantly enriched, suggesting that lysine acetylation mainly occurred on glycometabolism related proteins in this organelle. The latest studies on mitochondrial acetylome also revealed similar features in *A.thaliana* and *Pisum sativum* (König et al., 2014; Smith-Hammond et al., 2014b). In the nucleus, many DNA and chromatin assembling and organization related terms were significantly enriched (Figure 5A) as the well-described lysine acetylation on histones. Consistently, several nucleus and chromosome related terms were enriched on the nucleus in category of cellular components (Figure 5B).

Two possible factors may contribute to the differential clustering results in different organelles. Firstly, the intrinsic subcellular location and distribution of functional proteins in plant cells. For instance, chloroplast is the key organelle which is responsible for photosynthesis and most photosynthesis related proteins were located in chloroplast. Thus, we detected many photosynthesis related acetylation proteins in chloroplast and the clustering analysis results showed that photosynthesis related GO terms including photosynthetic membrane, photosystem, oxygen evolving complex, and thylakoid were significantly enriched in chloroplast. Secondly, the preferential subcellular distribution of diverse acetylases and deacetylases was another important factor. Taking an example, many acetylated sites were found to be located in both RuBisCo subunits in our study and previous studies in *A. thaliana* also found the lysine acetylation in both RBCL and RBCS presumably to regulate the activity of RuBisCo (Finkemeier et al., 2011; Wu et al., 2011). As RBCL is encoded by the chloroplast genome, it is probable that a protein lysine acetylase may exist in the chloroplast. Besides, histone deacetylases (HDACs) and histone acetyltransferases (HATs) were mainly located in the nucleus (Chen and Tian, 2007; Hirschey et al., 2011). It is well-known that histones participate in DNA and chromatin assembling and organization. Therefore, many DNA and chromatin assembling and organization related terms were significantly enriched in the nucleus.

### Motif analysis of lysine acetylated peptides

To identify possible specific sequence motifs surrounding acetylated lysine residues, we generated a type of sequence logo which computes the likelihood of amino acids being over-or underrepresented at the positions surrounding the acetylation site.

We identified five significantly enriched acetylation site motifs from 1127 unique sites accounting for 90% of sites identified. The five consensus sequence motifs were L^*^Kac, F^*^Kac, KacH, KacY, and KacF (Kac represents the acetylated lysine and ^*^ represents a random amino acid residu**e** (Supplementary Figure S2A). We noticed that aromatic amino acid including tyrosine (Y) and phenylalanine (F) appeared frequently in the consensus sequence motif. Previous studies of sequence motifs surrounding acetylated lysine residues in rice and Arabidopsis had not found a well-defined consensus sequence, but they reported charged amino acid histidine (H) and the aromatic amino acids (W, Y, and F) were presented in much lower frequency (Finkemeier et al., 2011; Nallamilli et al., 2014), which is contradictory with our study. Compared with the conserved motif sequence in bacteria, we found the motifs KacH and KacY were also conserved in bacteria (Zhang et al., 2009; Pan et al., 2014). Plants and bacteria may share some common conserved motifs surrounding acetylated lysine sites.

In the above subcellular prediction analysis, we noticed that approximately a half of all the acetylated proteins were chloroplast located. Thus, we also further studied the motif characteristics of all chloroplast related acetylated peptides. As shown in Supplementary Figure S2B, a total of five well-defined consensus motifs were extracted, of which all were overlapped with the conserved motifs in whole strawberry leaves acetylated peptides analysis. Motif analysis of all the photosynthesis related acetylated proteins only revealed two consensus motifs (Supplementary Figure S2C).

### Interaction networks analysis of lysine acetylated proteins in strawberry

**Figure 6:**
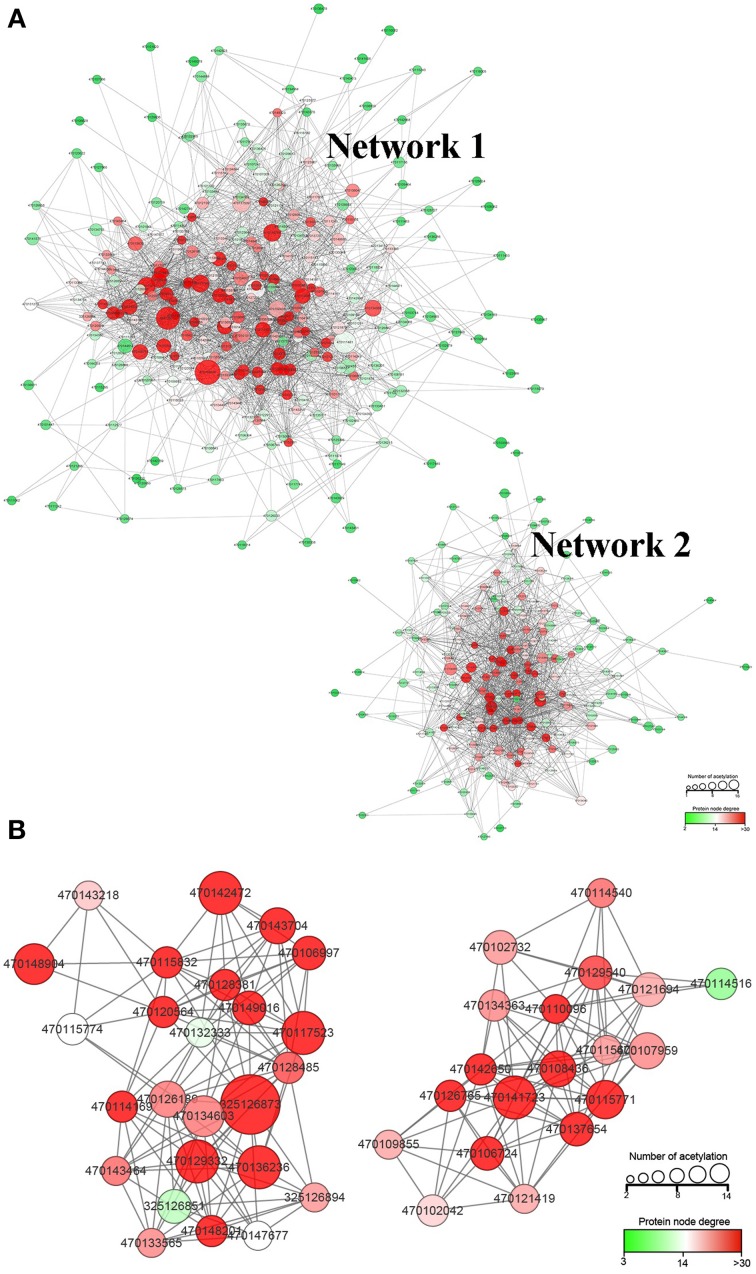
**Protein-protein interaction (PPI) network of all the lysine acetylated proteins. (A)**, the whole PPI network; **(B)**, the significantly enriched subcluster extracted from the whole PPI network.

To deeply understand how these acetylated proteins are related and how the acetylated proteins involved different pathways crosslink to each other, we constructed in protein-protein interaction (PPI) network for all of the acetylated proteins using STRING database and Cytoscape software (Figure 6).

Most proteins were clustered into two groups (Network 1 and Network 2). The detailed information of proteins involved in these two networks is presented in Supplementary Table 2. The degree of node is a very important parameter to evaluate the importance of node in network. Thus, we calculated the degree of each lysine acetylated protein. In the whole network, 21 lysine acetylated proteins were identified with degree over 40. The majority of the 21 Kac proteins were glycometabolism (such as tricarboxylic acid cycle, glycolysis) and photosynthesis related proteins, suggesting that the lysine acetylation on glycometabolism and photosynthesis related proteins may play important role in the regulation of strawberry growth and development. Among the 21 lysine acetylated proteins, phosphoglycerate kinase, chloroplastic-like, malate dehydrogenase, cytoplasmic-like, and malate dehydrogenase, mitochondrial-like had the highest degree. The lysine acetylation on malate dehydrogenase had been discovered in *Escherichia* coli, mammals, and *A. thaliana* (Kim et al., 2006; Zhang et al., 2013; König et al., 2014). Acetylation of malate dehydrogenase may be a conserved pattern in regulating mitochondrial energy metabolism on PTM level in organisms.

**Figure 7:**
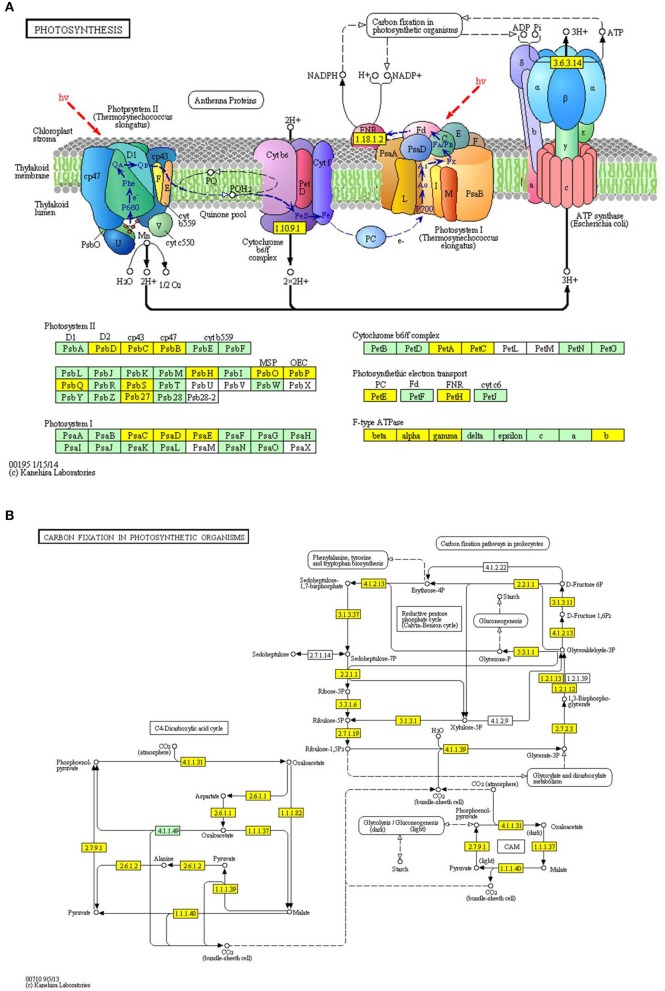
**Representative significantly enriched photosynthesis related KEGG pathways**. **(A)**, photosynthesis pathway; **(B)**, carbon fixation in photosynthetic organisms pathway. The acetylated proteins are marked in yellow.

By using the MCODE plug-in tool kit, we extracted several highly enriched interaction clusters from the whole interaction network. We found that both the top two significantly enriched clusters were photosynthesis related (Figure 6B and Supplementary Table 3), which is in consistence with the subcellular location prediction results that almost 50% acetylated proteins were located in chloroplast. The main members of these two clusters were components of photosynthetic system and many of them were acetylated in multi lysine sites. These acetylated photosynthesis related proteins can be roughly classified into two major groups according to their role in photosynthesis, namely light reaction related proteins including components of photosystem and components of photosynthetic electron transfer chain and carbon-fixation reaction (Calvin cycle) related proteins. Besides, in our KEGG pathway enrichment analysis, several photosynthetic related pathways were enriched (Supplementary Package 1) and two representative pathways are shown in Figure 7. Surprisingly, almost all the core parts of light reaction such as photosystems (I and II), electron transport chain, cytochrome b6f complex, and ATP synthase were lysine acetylated in several individual subunits, indicating lysine acetylation exists extensively in photosystem. Besides, many carbon fixation related enzymes were also acetylated in lysine sites. These further proved that many lysine acetylation occurs in the photosynthetic system in strawberry leaves.

### The possible role of lysine acetylation on photosynthesis system

In the present study, we identified 66 lysine acetylated photosynthesis related proteins in strawberry leaves (Supplementary Table 4). Previous two studies had identified nine and five lysine acetylated proteins involved in photosynthesis in *A. thaliana* by acetylome analysis, respectively (Finkemeier et al., 2011; Wu et al., 2011). Another study in rice only identified two photosynthesis related lysine acetylated proteins (Nallamilli et al., 2014). Our study expanded the dataset of lysine acetylated photosynthetic proteins greatly, which may facilitate the revealing of the mechanism of lysine acetylation regulated photosynthesis.

Protein post-translational modification may be involved in chloroplast metabolism regulation and photosynthetic machinery adjustment and phosphorylation is the best-characterized PTM in regulating photosynthetic performance (Lehtimäki et al., 2014). Our protein-protein interaction analysis and KEGG pathway enrichment analysis indicated the lysine acetylation on photosynthetic system was another important event in chloroplast and may play important role in photosynthesis regulation.

## Conclusion

In this work, by combining high affinity enrichment of acetylated peptides, high sensitive mass spectrometry, and advanced bioinformatic tools, we comprehensively investigated the lysine acetylome in strawberry leaves. The identification of 1392 acetylation sites from 684 proteins expanded the catalog of lysine acetylome in plants. Functional characterization of lysine acetylated proteins further indicated that lysine acetylation was involved in diverse biological processes and cellular components. Besides, chloroplast was the primary organelle where lysine acetylation occurred suggesting that lysine acetylation plays important role in photosynthesis regulation. In addition, motif analysis extracted five consensus sequence motifs. This study greatly expanded our knowledge of the plant acetylome and laid a sound foundation for the study of lysine acetylation in plants.

### Conflict of interest statement

The authors declare that the research was conducted in the absence of any commercial or financial relationships that could be construed as a potential conflict of interest.
