# Supplementary material for: Global analysis of lysine acetylation in strawberry leaves
Source: Front Plant Sci. 2015 Sep 15;6:739. doi: 10.3389/fpls.2015.00739 (PMC4569977; doi:10.3389/fpls.2015.00739)
Supplement: Supplementary file 5 [file DataSheet1.ZIP › Ascorbate and aldarate metabolism - Fragaria vesca (woodland strawberry).html]

KEGG PATHWAY: Ascorbate and aldarate metabolism - Fragaria vesca (woodland strawberry)


|  |  |
| --- | --- |
| **Ascorbate and aldarate metabolism - Fragaria vesca (woodland strawberry)** |  |

[
Pathway menu
| Organism menu
| Pathway entry
| Download KGML
| User data mapping
]

|  |  |
| --- | --- |
| Reference pathway Reference pathway (KO) Reference pathway (EC) Reference pathway (Reaction) -----< Set personalized menu >----- -----< Sort below by alphabet >----- Homo sapiens (human) Homo sapiens (human) + Disease/drug Pan troglodytes (chimpanzee) Pan paniscus (bonobo) Gorilla gorilla gorilla (western lowland gorilla) Pongo abelii (Sumatran orangutan) Macaca mulatta (rhesus monkey) Macaca fascicularis (crab-eating macaque) Mus musculus (mouse) Rattus norvegicus (rat) Cricetulus griseus (Chinese hamster) Heterocephalus glaber (naked mole rat) Tupaia chinensis (Chinese tree shrew) Canis familiaris (dog) Ailuropoda melanoleuca (giant panda) Felis catus (domestic cat) Panthera tigris altaica (Amur tiger) Bos taurus (cow) Bos mutus (wild yak) Pantholops hodgsonii (chiru) Capra hircus (goat) Ovis aries (sheep) Sus scrofa (pig) Camelus ferus (Wild Bactrian camel) Balaenoptera acutorostrata scammoni (minke whale) Lipotes vexillifer (Yangtze River dolphin) Equus caballus (horse) Myotis brandtii (Brandt's bat) Myotis davidii Pteropus alecto (black flying fox) Monodelphis domestica (opossum) Sarcophilus harrisii (Tasmanian devil) Ornithorhynchus anatinus (platypus) Gallus gallus (chicken) Meleagris gallopavo (turkey) Taeniopygia guttata (zebra finch) Ficedula albicollis (collared flycatcher) Pseudopodoces humilis (Tibetan ground-tit) Anas platyrhynchos (mallard) Falco peregrinus (peregrine falcon) Falco cherrug (Saker falcon) Columba livia (rock pigeon) Alligator sinensis (Chinese alligator) Alligator mississippiensis (American alligator) Pelodiscus sinensis (Chinese soft-shelled turtle) Chelonia mydas (green sea turtle) Anolis carolinensis (green anole) Python bivittatus (Burmese python) Xenopus laevis (African clawed frog) Xenopus tropicalis (western clawed frog) Danio rerio (zebrafish) Takifugu rubripes (torafugu) Maylandia zebra (zebra mbuna) Oryzias latipes (Japanese medaka) Xiphophorus maculatus (southern platyfish) Latimeria chalumnae (coelacanth) Callorhinchus milii (elephant shark) Branchiostoma floridae (Florida lancelet) Ciona intestinalis (sea squirt) Strongylocentrotus purpuratus (purple sea urchin) Drosophila melanogaster (fruit fly) Drosophila pseudoobscura pseudoobscura Drosophila ananassae Drosophila erecta Drosophila persimilis Drosophila sechellia Drosophila simulans Drosophila willistoni Drosophila yakuba Drosophila grimshawi Drosophila mojavensis Drosophila virilis Anopheles gambiae (mosquito) Aedes aegypti (yellow fever mosquito) Culex quinquefasciatus (southern house mosquito) Apis mellifera (honey bee) Nasonia vitripennis (jewel wasp) Tribolium castaneum (red flour beetle) Bombyx mori (domestic silkworm) Acyrthosiphon pisum (pea aphid) Pediculus humanus corporis (human body louse) Ixodes scapularis (black-legged tick) Caenorhabditis elegans (nematode) Caenorhabditis briggsae Brugia malayi (filaria) Loa loa (eye worm) Trichinella spiralis Schistosoma mansoni Nematostella vectensis (sea anemone) Hydra vulgaris Trichoplax adhaerens Amphimedon queenslandica (sponge) Arabidopsis thaliana (thale cress) Arabidopsis lyrata (lyrate rockcress) Capsella rubella Eutrema salsugineum Citrus sinensis (Valencia orange) Citrus clementina (mandarin orange) Theobroma cacao (cacao) Glycine max (soybean) Phaseolus vulgaris (common bean) Medicago truncatula (barrel medic) Cicer arietinum (chickpea) Fragaria vesca (woodland strawberry) Prunus persica (peach) Prunus mume (Japanese apricot) Malus domestica (apple) Cucumis sativus (cucumber) Cucumis melo (muskmelon) Ricinus communis (castor bean) Populus trichocarpa (black cottonwood) Vitis vinifera (wine grape) Solanum lycopersicum (tomato) Solanum tuberosum (potato) Oryza sativa japonica (Japanese rice) (RefSeq) Oryza sativa japonica (Japanese rice) (RAPDB) Oryza brachyantha (malo sina) Brachypodium distachyon Sorghum bicolor (sorghum) Zea mays (maize) Setaria italica (foxtail millet) Amborella trichopoda Selaginella moellendorffii Physcomitrella patens subsp. patens Chlamydomonas reinhardtii Volvox carteri f. nagariensis Ostreococcus lucimarinus Ostreococcus tauri Bathycoccus prasinos Micromonas sp. RCC299 Micromonas pusilla Coccomyxa subellipsoidea Chlorella variabilis Cyanidioschyzon merolae Galdieria sulphuraria Chondrus crispus (carragheen) Eremothecium cymbalariae Kluyveromyces lactis Lachancea thermotolerans Zygosaccharomyces rouxii Torulaspora delbrueckii Pichia pastoris Debaryomyces hansenii Scheffersomyces stipitis Spathaspora passalidarum Lodderomyces elongisporus Candida albicans Candida tropicalis Candida orthopsilosis Candida dubliniensis Candida tenuis Yarrowia lipolytica Clavispora lusitaniae Neurospora crassa Sordaria macrospora Podospora anserina Thielavia terrestris Myceliophthora thermophila Chaetomium thermophilum Magnaporthe oryzae Togninia minima Fusarium graminearum Nectria haematococca Trichoderma reesei Metarhizium acridum Metarhizium anisopliae Cordyceps militaris Verticillium alfalfae Eutypa lata Sclerotinia sclerotiorum Botrytis cinerea Marssonina brunnea Aspergillus nidulans Aspergillus fumigatus Aspergillus oryzae Aspergillus niger Aspergillus flavus Aspergillus clavatus Neosartorya fischeri Penicillium chrysogenum Coccidioides immitis Coccidioides posadasii Paracoccidioides brasiliensis Uncinocarpus reesii Arthroderma benhamiae Trichophyton verrucosum Ajellomyces capsulatus Phaeosphaeria nodorum Pyrenophora teres Bipolaris zeicola Bipolaris sorokiniana Bipolaris oryzae Zymoseptoria tritici Pseudocercospora fijiensis Baudoinia compniacensis Neofusicoccum parvum Tuber melanosporum Cryptococcus neoformans JEC21 Cryptococcus neoformans B-3501A Cryptococcus gattii Tremella mesenterica Dichomitus squalens Stereum hirsutum Phanerochaete carnosa Punctularia strigosozonata Auricularia delicata Fomitiporia mediterranea Gloeophyllum trabeum Laccaria bicolor Moniliophthora perniciosa Moniliophthora roreri Coprinopsis cinerea Schizophyllum commune Agaricus bisporus var. burnettii JB137-S8 Agaricus bisporus var. bisporus H97 Coniophora puteana Serpula lacrymans Ustilago maydis Pseudozyma flocculosa Malassezia globosa Puccinia graminis Melampsora larici-populina Wallemia sebi Monosiga brevicollis Dictyostelium discoideum (cellular slime mold) Dictyostelium purpureum (cellular slime mold) Dictyostelium fasciculatum (cellular slime mold) Acanthamoeba castellanii Toxoplasma gondii Tetrahymena thermophila Paramecium tetraurelia Phaeodactylum tricornutum Thalassiosira pseudonana Phytophthora infestans Nannochloropsis gaditana Emiliania huxleyi Guillardia theta Trypanosoma cruzi Leishmania major Leishmania infantum Leishmania donovani Leishmania mexicana Leishmania braziliensis Naegleria gruberi Trichomonas vaginalis Escherichia coli K-12 MG1655 Escherichia coli K-12 W3110 Escherichia coli K-12 DH10B Escherichia coli K-12 MC4100(MuLac) BW2952 Escherichia coli K-12 MDS42 Escherichia coli O157:H7 EDL933 (EHEC) Escherichia coli O157:H7 Sakai (EHEC) Escherichia coli O157:H7 EC4115 (EHEC) Escherichia coli O157:H7 TW14359 (EHEC) Escherichia coli O157:H7 Xuzhou21 (EHEC) Escherichia coli O26:H11 11368 (EHEC) Escherichia coli O111:H- 11128 (EHEC) Escherichia coli O103:H2 12009 (EHEC) Escherichia coli O127:H6 E2348/69 (EPEC) Escherichia coli O55:H7 CB9615 (EPEC) Escherichia coli O55:H7 RM12579 (EPEC) Escherichia coli O6:K2:H1 CFT073 (UPEC) Escherichia coli O6:K15:H31 536 (UPEC) Escherichia coli O18:K1:H7 UTI89 (UPEC) Escherichia coli APEC O1 (APEC) Escherichia coli O9 HS (commensal) Escherichia coli E24377A (ETEC) Escherichia coli SMS-3-5 (environmental) Escherichia coli O152:H28 SE11 (commensal) Escherichia coli O8 IAI1 (commensal) Escherichia coli O81 ED1a (commensal) Escherichia coli 55989 (EAEC) Escherichia coli O7:K1 IAI39 (ExPEC) Escherichia coli O7:K1 CE10 Escherichia coli O17:K52:H18 UMN026 (ExPEC) Escherichia coli O45:K1:H7 S88 (ExPEC) Escherichia coli O44:H18 042 (EAEC) Escherichia coli O83:H1 NRG 857C Escherichia coli O78:H11:K80 H10407 (ETEC) Escherichia coli O150:H5 SE15 (commensal) Escherichia coli O104:H4 2009EL-2071 Escherichia coli O104:H4 2009EL-2050 Escherichia coli O104:H4 2011C-3493 Escherichia coli ATCC 8739 Escherichia coli B REL606 Escherichia coli BL21-Gold(DE3)pLysS AG Escherichia coli KO11FL Escherichia coli KO11FL Escherichia coli ABU 83972 Escherichia coli DH1 Escherichia coli DH1 Escherichia coli IHE3034 Escherichia coli NA114 Escherichia coli UM146 Escherichia coli UMNK88 Escherichia coli W Escherichia coli W Escherichia coli clone D i14 Escherichia coli clone D i2 Escherichia coli P12b Escherichia coli BL21(DE3) Escherichia coli BL21(DE3) Escherichia coli LF82 Escherichia coli APEC O78 Escherichia coli LY180 Escherichia coli PMV-1 Escherichia coli JJ1886 Escherichia coli O145:H28 RM13514 (EHEC) Escherichia coli O145:H28 RM13516 (EHEC) Escherichia fergusonii Salmonella enterica subsp. enterica serovar Typhi CT18 Salmonella enterica subsp. enterica serovar Typhi Ty2 Salmonella enterica subsp. enterica serovar Typhi P-stx-12 Salmonella enterica subsp. enterica serovar Typhi Ty21a Salmonella enterica subsp. enterica serovar Typhimurium LT2 Salmonella enterica subsp. enterica serovar Typhimurium 14028S Salmonella enterica subsp. enterica serovar Typhimurium D23580 Salmonella enterica subsp. enterica serovar Typhimurium SL1344 Salmonella enterica subsp. enterica serovar Typhimurium T000240 Salmonella enterica subsp. enterica serovar Typhimurium UK-1 Salmonella enterica subsp. enterica serovar Typhimurium ST4/74 Salmonella enterica subsp. enterica serovar Typhimurium 798 Salmonella enterica subsp. enterica serovar Typhimurium U288 Salmonella enterica subsp. enterica serovar Typhimurium var. 5- CFSAN001921 Salmonella enterica subsp. enterica serovar Typhimurium 08-1736 Salmonella enterica subsp. enterica serovar Typhimurium DT2 Salmonella enterica subsp. enterica serovar Typhimurium DT104 Salmonella enterica subsp. enterica serovar Paratyphi A ATCC9150 Salmonella enterica subsp. enterica serovar Paratyphi A AKU12601 Salmonella enterica subsp. enterica serovar Paratyphi B Salmonella enterica subsp. enterica serovar Paratyphi C Salmonella enterica subsp. enterica serovar Choleraesuis Salmonella enterica subsp. enterica serovar Heidelberg SL476 Salmonella enterica subsp. enterica serovar Heidelberg B182 Salmonella enterica subsp. enterica Serovar Heidelberg CFSAN002069 Salmonella enterica subsp. enterica serovar Heidelberg 41578 Salmonella enterica subsp. enterica serovar Newport SL254 Salmonella enterica subsp. enterica serovar Newport USMARC-S3124.1 Salmonella enterica subsp. enterica serovar Schwarzengrund Salmonella enterica subsp. enterica serovar Agona SL483 Salmonella enterica subsp. enterica serovar Agona 24249 Salmonella enterica subsp. enterica serovar Dublin Salmonella enterica subsp. enterica serovar Gallinarum 287/91 Salmonella enterica subsp. enterica serovar Gallinarum/pullorum RKS5078 Salmonella enterica subsp. enterica serovar Gallinarum/pullorum CDC1983-67 Salmonella enterica subsp. enterica serovar Enteritidis Salmonella enterica subsp. enterica serovar Javiana Salmonella enterica subsp. enterica Serovar Cubana Salmonella enterica subsp. enterica serovar Bareilly Salmonella enterica subsp. enterica serovar Pullorum Salmonella enterica subsp. enterica serovar Bovismorbificans Salmonella enterica subsp. enterica serovar Thompson Salmonella enterica subsp. arizonae Salmonella bongori NCTC 12419 Salmonella bongori N268-08 Yersinia pestis CO92 (biovar Orientalis) Yersinia pestis KIM10+ (biovar Mediaevalis) Yersinia pestis Antiqua (biovar Antiqua) Yersinia pestis Nepal516 (biovar Antiqua) Yersinia pestis 91001 (biovar Microtus) Yersinia pestis Pestoides F Yersinia pestis Angola Yersinia pestis Z176003 Yersinia pestis A1122 Yersinia pestis D106004 Yersinia pestis D182038 Yersinia pestis biovar Medievalis Harbin 35 Yersinia pseudotuberculosis IP32953 (serotype I) Yersinia pseudotuberculosis IP31758 (serotype O:1b) Yersinia pseudotuberculosis YPIII Yersinia pseudotuberculosis PB1/+ Yersinia enterocolitica subsp. enterocolitica 8081 Yersinia enterocolitica subsp. palearctica 105.5R(r) Yersinia enterocolitica subsp. palearctica Y11 Yersinia enterocolitica LC20 Yersinia similis Shigella flexneri 301 (serotype 2a) Shigella flexneri 2457T (serotype 2a) Shigella flexneri 8401 (serotype 5b) Shigella flexneri 2002017 (serotype Fxv) Shigella sonnei Ss046 Shigella sonnei 53G Shigella boydii Sb227 Shigella boydii CDC 3083-94 Shigella dysenteriae Sd197 Shigella dysenteriae 1617 Pectobacterium atrosepticum SCRI1043 Pectobacterium atrosepticum JG10-08 Pectobacterium carotovorum subsp. carotovorum PC1 Pectobacterium carotovorum subsp. carotovorum PCC21 Pectobacterium wasabiae Pectobacterium sp. SCC3193 Erwinia tasmaniensis Erwinia pyrifoliae Ep1/96 Erwinia pyrifoliae DSM 12163 Erwinia amylovora CFBP1430 Erwinia billingiae Erwinia sp. Ejp617 Photorhabdus luminescens Photorhabdus asymbiotica Sodalis glossinidius (Glossina spp.) Sodalis sp. HS1 Candidatus Sodalis pierantonius Enterobacter sp. 638 Enterobacter cloacae subsp. cloacae ATCC 13047 Enterobacter cloacae subsp. cloacae ENHKU01 Enterobacter cloacae subsp. cloacae NCTC 9394 Enterobacter cloacae EcWSU1 Enterobacter cloacae subsp. dissolvens SDM Enterobacter lignolyticus Enterobacter asburiae LF7a Enterobacter asburiae L1 Enterobacter aerogenes KCTC 2190 Enterobacter aerogenes EA1509E Enterobacter sp. R4-368 Cronobacter turicensis Klebsiella pneumoniae subsp. pneumoniae MGH 78578 Klebsiella pneumoniae NTUH-K2044 Klebsiella pneumoniae subsp. pneumoniae HS11286 Klebsiella pneumoniae subsp. pneumoniae 1084 Klebsiella pneumoniae 342 Klebsiella pneumoniae KCTC 2242 Klebsiella pneumoniae Klebsiella pneumoniae JM45 Klebsiella pneumoniae CG43 Klebsiella pneumoniae 30660/NJST258\_1 Klebsiella pneumoniae 30684/NJST258\_2 Klebsiella variicola Klebsiella oxytoca KCTC 1686 Klebsiella oxytoca E718 Klebsiella oxytoca HKOPL1 Citrobacter koseri Citrobacter rodentium Citrobacter freundii Serratia proteamaculans Serratia plymuthica AS9 Serratia plymuthica 4Rx13 Serratia plymuthica S13 Serratia sp. AS12 Serratia sp. AS13 Serratia marcescens FGI94 Serratia marcescens WW4 Serratia liquefaciens Serratia sp. ATCC 39006 Serratia fonticola Proteus mirabilis HI4320 Proteus mirabilis BB2000 Edwardsiella ictaluri Edwardsiella tarda EIB202 Edwardsiella tarda FL6-60 Edwardsiella piscicida C07-087 Dickeya dadantii Ech703 Dickeya dadantii Ech586 Dickeya dadantii 3937 Dickeya zeae Xenorhabdus nematophila Pantoea ananatis LMG 20103 Pantoea ananatis LMG 5342 Pantoea ananatis AJ13355 Pantoea ananatis PA13 Pantoea vagans Pantoea sp. At-9b Rahnella sp. Y9602 Rahnella aquatilis CIP 78.65 = ATCC 33071 Rahnella aquatilis HX2 Providencia stuartii Shimwellia blattae Morganella morganii Raoultella ornithinolytica Enterobacteriaceae bacterium FGI 57 Plautia stali symbiont Haemophilus influenzae Rd KW20 (serotype d) Haemophilus influenzae PittGG (nontypeable) Haemophilus ducreyi Haemophilus parasuis SH0165 Haemophilus parasuis ZJ0906 Haemophilus somnus 129PT Haemophilus somnus 2336 Pasteurella multocida subsp. multocida Pm70 Pasteurella multocida subsp. multocida HN06 Pasteurella multocida subsp. multocida 3480 Pasteurella multocida 36950 Mannheimia succiniciproducens Mannheimia haemolytica USDA-ARS-SAM-185 Mannheimia haemolytica USDA-ARS-USMARC-183 Mannheimia haemolytica M42548 Mannheimia haemolytica D153 Mannheimia haemolytica D171 Mannheimia haemolytica D174 Mannheimia haemolytica USMARC\_2286 Mannheimia varigena USDA-ARS-USMARC-1261 Mannheimia varigena USDA-ARS-USMARC-1296 Mannheimia varigena USDA-ARS-USMARC-1312 Mannheimia varigena USDA-ARS-USMARC-1388 Actinobacillus pleuropneumoniae L20 (serotype 5b) Actinobacillus pleuropneumoniae JL03 (serotype 3) Actinobacillus pleuropneumoniae AP76 (serotype 7) Actinobacillus succinogenes Actinobacillus suis H91-0380 Aggregatibacter aphrophilus Aggregatibacter actinomycetemcomitans D11S-1 Aggregatibacter actinomycetemcomitans ANH9381 Aggregatibacter actinomycetemcomitans D7S-1 Aggregatibacter actinomycetemcomitans HK1651 Gallibacterium anatis Bibersteinia trehalosi USDA-ARS-USMARC-192 Bibersteinia trehalosi USDA-ARS-USMARC-188 Bibersteinia trehalosi USDA-ARS-USMARC-189 Bibersteinia trehalosi USDA-ARS-USMARC-190 Xylella fastidiosa 9a5c Xylella fastidiosa Temecula1 Xylella fastidiosa M12 Xylella fastidiosa M23 Xylella fastidiosa subsp. fastidiosa GB514 Xanthomonas campestris pv. campestris ATCC 33913 Xanthomonas campestris pv. campestris 8004 Xanthomonas campestris pv. campestris B100 Xanthomonas campestris pv. raphani Xanthomonas campestris pv. vesicatoria Xanthomonas citri pv. citri 306 Xanthomonas citri subsp. citri Aw12879 Xanthomonas alfalfae Xanthomonas axonopodis Xac29-1 Xanthomonas oryzae pv. oryzae KACC 10331 Xanthomonas oryzae pv. oryzae MAFF311018 Xanthomonas oryzae pv. oryzae PXO99A Xanthomonas oryzae pv. oryzicola Xanthomonas albilineans Xanthomonas fuscans Stenotrophomonas maltophilia K279a Stenotrophomonas maltophilia R551-3 Stenotrophomonas maltophilia JV3 Stenotrophomonas maltophilia D457 Pseudoxanthomonas suwonensis Pseudoxanthomonas spadix Frateuria aurantia Rhodanobacter denitrificans Dyella jiangningensis Dyella japonica Vibrio cholerae O1 biovar El Tor N16961 Vibrio cholerae O1 2010EL-1786 Vibrio cholerae MJ-1236 Vibrio cholerae O395 Vibrio cholerae O395 Vibrio cholerae M66-2 Vibrio cholerae IEC224 Vibrio cholerae LMA3984-4 Vibrio vulnificus CMCP6 Vibrio vulnificus YJ016 Vibrio vulnificus MO6-24/O Vibrio parahaemolyticus RIMD 2210633 Vibrio parahaemolyticus BB22OP Vibrio parahaemolyticus O1:K33 CDC\_K4557 Vibrio parahaemolyticus O1:Kuk FDA\_R31 Vibrio parahaemolyticus UCM-V493 Vibrio campbellii Vibrio campbellii Vibrio alginolyticus Vibrio splendidus Vibrio sp. Ex25 Vibrio sp. EJY3 Vibrio furnissii Vibrio nigripulchritudo Vibrio anguillarum 775 Vibrio anguillarum M3 Aiivibrio fischeri MJ11 Aliivibrio salmonicida Photobacterium profundum Pseudomonas aeruginosa PAO1 Pseudomonas aeruginosa PAO1-VE13 Pseudomonas aeruginosa PAO1-VE2 Pseudomonas aeruginosa UCBPP-PA14 Pseudomonas aeruginosa PA7 Pseudomonas aeruginosa LESB58 Pseudomonas aeruginosa M18 Pseudomonas aeruginosa NCGM2.S1 Pseudomonas aeruginosa DK2 Pseudomonas aeruginosa B136-33 Pseudomonas aeruginosa RP73 Pseudomonas aeruginosa PA1 Pseudomonas aeruginosa PA1R Pseudomonas aeruginosa MTB-1 Pseudomonas aeruginosa LES431 Pseudomonas aeruginosa SCV20265 Pseudomonas aeruginosa PA38182 Pseudomonas aeruginosa YL84 Pseudomonas aeruginosa c7447m Pseudomonas aeruginosa PAO581 Pseudomonas putida KT2440 Pseudomonas putida F1 Pseudomonas putida GB-1 Pseudomonas putida W619 Pseudomonas putida S16 Pseudomonas putida BIRD-1 Pseudomonas putida ND6 Pseudomonas putida DOT-T1E Pseudomonas putida HB3267 Pseudomonas putida H8234 Pseudomonas putida NBRC 14164 Pseudomonas sp. UW4 Pseudomonas syringae pv. tomato DC3000 Pseudomonas syringae pv. syringae B728a Pseudomonas syringae CC1557 Pseudomonas syringae pv. phaseolicola 1448A Pseudomonas cichorii Pseudomonas protegens Pf-5 Pseudomonas protegens CHA0 Pseudomonas fluorescens Pf0-1 Pseudomonas fluorescens SBW25 Pseudomonas fluorescens F113 Pseudomonas fluorescens A506 Pseudomonas poae Pseudomonas entomophila Pseudomonas mendocina ymp Pseudomonas mendocina NK-01 Pseudomonas stutzeri A1501 Pseudomonas stutzeri ATCC 17588 Pseudomonas stutzeri DSM 4166 Pseudomonas stutzeri CCUG 29243 Pseudomonas stutzeri DSM 10701 Pseudomonas stutzeri RCH2 Pseudomonas brassicacearum subsp. brassicacearum NFM421 Pseudomonas brassicacearum DF41 Pseudomonas fulva Pseudomonas denitrificans Pseudomonas resinovorans Pseudomonas sp. VLB120 Pseudomonas sp. TKP Pseudomonas monteilii SB3078 Pseudomonas monteilii SB3101 Pseudomonas knackmussii Pseudomonas chlororaphis Cellvibrio japonicus Azotobacter vinelandii DJ Azotobacter vinelandii CA Azotobacter vinelandii CA6 Psychrobacter arcticus Psychrobacter cryohalolentis Psychrobacter sp. PRwf-1 Psychrobacter sp. G Acinetobacter sp. ADP1 Acinetobacter oleivorans Acinetobacter baumannii ATCC 17978 Acinetobacter baumannii SDF Acinetobacter baumannii AYE Acinetobacter baumannii ACICU Acinetobacter baumannii AB0057 Acinetobacter baumannii AB307-0294 Acinetobacter baumannii 1656-2 Acinetobacter baumannii MDR-ZJ06 Acinetobacter baumannii MDR-TJ Acinetobacter baumannii TCDC-AB0715 Acinetobacter baumannii TYTH-1 Acinetobacter baumannii BJAB07104 Acinetobacter baumannii BJAB0715 Acinetobacter baumannii BJAB0868 Acinetobacter baumannii ZW85-1 Acinetobacter calcoaceticus Shewanella frigidimarina Idiomarina loihiensis L2TR Idiomarina loihiensis GSL 199 Colwellia psychrerythraea Pseudoalteromonas atlantica Marinobacter hydrocarbonoclasticus VT8 Marinobacter hydrocarbonoclasticus ATCC 49840 Marinobacter adhaerens Marinobacter sp. BSs20148 Alteromonas macleodii Deep ecotype Alteromonas macleodii ATCC 27126 Alteromonas macleodii Balearic Sea AD45 Alteromonas macleodii English Channel 673 Alteromonas macleodii English Channel 615 Alteromonas macleodii Black Sea 11 Alteromonas macleodii AltDE1 Alteromonas macleodii Ionian Sea U4 Alteromonas macleodii Ionian Sea U7 Alteromonas macleodii Ionian Sea U8 Alteromonas macleodii Ionian Sea UM4b Alteromonas macleodii Ionian Sea UM7 Alteromonas macleodii Aegean Sea MED64 Alteromonas sp. SN2 Alteromonas australica Glaciecola sp. 4H-3-7+YE-5 Glaciecola nitratireducens Glaciecola psychrophila Psychromonas ingrahamii Teredinibacter turnerae Ferrimonas balearica Legionella pneumophila subsp. pneumophila Philadelphia 1 Legionella pneumophila subsp. pneumophila HL06041035 Legionella pneumophila subsp. pneumophila Thunder Bay Legionella pneumophila Lens Legionella pneumophila Paris Legionella pneumophila Corby Legionella pneumophila 2300/99 Alcoy Legionella longbeachae Francisella cf. novicida Fx1 Francisella cf. novicida 3523 Francisella sp. TX077308 Methylophaga nitratireducenticrescens Methylophaga frappieri Nitrosococcus oceani Nitrosococcus halophilus Nitrosococcus watsonii Allochromatium vinosum Thiocystis violascens Thioflavicoccus mobilis Halorhodospira halophila Thioalkalivibrio sulfidiphilus Thioalkalivibrio sp. K90mix Thioalkalivibrio nitratireducens Spiribacter salinus Spiribacter sp. UAH-SP71 Halothiobacillus neapolitanus Hahella chejuensis Chromohalobacter salexigens Halomonas elongata Halomonas campaniensis Alcanivorax borkumensis Alcanivorax dieselolei Marinomonas sp. MWYL1 Marinomonas mediterranea Marinomonas posidonica Thalassolituus oleivorans MIL-1 Thalassolituus oleivorans R6-15 Aeromonas hydrophila subsp. hydrophila ATCC 7966 Aeromonas hydrophila ML09-119 Aeromonas hydrophila YL17 Aeromonas hydrophila AL09-71 Aeromonas hydrophila pc104A Aeromonas salmonicida Aeromonas veronii Aeromonas media Tolumonas auensis Acidithiobacillus ferrooxidans ATCC 53993 Acidithiobacillus ferrooxidans ATCC 23270 Acidithiobacillus caldus Acidithiobacillus ferrivorans Gilliamella apicola Simiduia agarivorans Gamma proteobacterium HdN1 Chromobacterium violaceum Pseudogulbenkiania sp. NH8B Ralstonia solanacearum GMI1000 Ralstonia solanacearum CFBP2957 Ralstonia solanacearum PSI07 Ralstonia solanacearum Po82 Ralstonia solanacearum CMR15 Ralstonia solanacearum FQY\_4 Ralstonia pickettii 12J Ralstonia pickettii 12D Ralstonia pickettii DTP0602 Ralstonia eutropha JMP134 Ralstonia eutropha H16 Cupriavidus necator N-1 Cupriavidus metallidurans Cupriavidus taiwanensis Burkholderia mallei ATCC 23344 Burkholderia mallei SAVP1 Burkholderia mallei NCTC 10229 Burkholderia mallei NCTC 10247 Burkholderia pseudomallei K96243 Burkholderia pseudomallei 1710b Burkholderia pseudomallei 1106a Burkholderia pseudomallei 668 Burkholderia pseudomallei MSHR346 Burkholderia pseudomallei MSHR305 Burkholderia pseudomallei MSHR511 Burkholderia pseudomallei MSHR146 Burkholderia pseudomallei MSHR520 Burkholderia pseudomallei 1026b Burkholderia pseudomallei BPC006 Burkholderia pseudomallei NCTC 13179 Burkholderia thailandensis E264 Burkholderia thailandensis 2002721723 Burkholderia thailandensis E444 Burkholderia thailandensis H0587 Burkholderia thailandensis MSMB121 Burkholderia vietnamiensis Burkholderia lata Burkholderia cenocepacia AU1054 Burkholderia cenocepacia HI2424 Burkholderia cenocepacia MC0-3 Burkholderia cenocepacia J2315 Burkholderia ambifaria AMMD Burkholderia ambifaria MC40-6 Burkholderia multivorans ATCC 17616 (JGI) Burkholderia multivorans ATCC 17616 (Tohoku) Burkholderia cepacia Burkholderia xenovorans Burkholderia phymatum Burkholderia phytofirmans Burkholderia glumae Burkholderia sp. CCGE1001 Burkholderia sp. CCGE1002 Burkholderia sp. CCGE1003 Burkholderia rhizoxinica Burkholderia gladioli Burkholderia sp. YI23 Burkholderia sp. KJ006 Burkholderia phenoliruptrix Burkholderia sp. RPE64 Polynucleobacter necessarius subsp. asymbioticus Polynucleobacter necessarius subsp. necessarius Pandoraea pnomenusa 3kgm Pandoraea pnomenusa RB38 Pandoraea sp. RB-44 Bordetella pertussis Tohama I Bordetella pertussis CS Bordetella pertussis 18323 Bordetella parapertussis 12822 Bordetella parapertussis Bpp5 Bordetella bronchiseptica RB50 Bordetella bronchiseptica MO149 Bordetella bronchiseptica 253 Bordetella petrii Bordetella avium Bordetella holmesii Achromobacter xylosoxidans A8 Achromobacter xylosoxidans NH44784-1996 Achromobacter xylosoxidans NBRC 15126 = ATCC 27061 Taylorella equigenitalis MCE9 Taylorella equigenitalis ATCC 35865 Taylorella equigenitalis 14/56 Taylorella asinigenitalis MCE3 Taylorella asinigenitalis 14/45 Pusillimonas sp. T7-7 Advenella kashmirensis Advenella mimigardefordensis Castellaniella defragrans Rhodoferax ferrireducens Polaromonas sp. JS666 Polaromonas naphthalenivorans Acidovorax citrulli Acidovorax sp. JS42 Acidovorax ebreus Acidovorax avenae Acidovorax sp. KKS102 Verminephrobacter eiseniae Delftia acidovorans Delftia sp. Cs1-4 Variovorax paradoxus S110 Variovorax paradoxus EPS Variovorax paradoxus B4 Comamonas testosteroni CNB-2 Comamonas testosteroni TK102 Alicycliphilus denitrificans BC Alicycliphilus denitrificans K601 Ramlibacter tataouinensis Symbiobacter mobilis Methylibium petroleiphilum Janthinobacterium agaricidamnosum Herbaspirillum seropedicae Collimonas fungivorans Leptothrix cholodnii Thiomonas intermedia Thiomonas arsenitoxydans Rubrivivax gelatinosus Nitrosomonas europaea Nitrosomonas eutropha Nitrosomonas sp. AL212 Nitrosomonas sp. Is79A3 Nitrosospira multiformis Aromatoleum aromaticum Azoarcus sp. BH72 Azoarcus sp. KH32C Dechloromonas aromatica Thauera sp. MZ1T Dechlorosoma suillum Thiobacillus denitrificans Sulfuricella denitrificans Methylotenera mobilis Methylovorus glucosetrophus Methylovorus sp. MP688 Accumulibacter phosphatis Sideroxydans lithotrophicus Gallionella capsiferriformans Beta proteobacterium CB Sulfuricurvum kujiense Campylobacter coli 76339 Campylobacter coli RM5611 Arcobacter nitrofigilis Sulfurospirillum multivorans Geobacter uraniireducens Pelobacter carbinolicus Desulfovibrio vulgaris Hildenborough Desulfovibrio vulgaris DP4 Desulfovibrio vulgaris Miyazaki F Desulfovibrio vulgaris RCH1 Desulfovibrio alaskensis Desulfovibrio desulfuricans ATCC 27774 Desulfovibrio aespoeensis Desulfovibrio africanus Desulfovibrio gigas Bdellovibrio bacteriovorus Tiberius Bdellovibrio exovorus Bacteriovorax marinus Desulfotalea psychrophila Desulfocapsa sulfexigens Desulfobacterium autotrophicum Desulfobacula toluolica Myxococcus xanthus Myxococcus fulvus Myxococcus stipitatus Corallococcus coralloides Stigmatella aurantiaca Sorangium cellulosum So ce 56 Sorangium cellulosum So0157-2 Haliangium ochraceum Syntrophus aciditrophicus Mesorhizobium loti Mesorhizobium ciceri Mesorhizobium opportunistum Mesorhizobium australicum Chelativorans sp. BNC1 Parvibaculum lavamentivorans Sinorhizobium meliloti 1021 Sinorhizobium meliloti AK83 Sinorhizobium meliloti BL225C Sinorhizobium meliloti SM11 Sinorhizobium meliloti Rm41 Sinorhizobium meliloti GR4 Sinorhizobium meliloti 2011 Sinorhizobium medicae Sinorhizobium fredii NGR234 Sinorhizobium fredii HH103 Sinorhizobium fredii USDA 257 Ensifer adhaerens Agrobacterium fabrum Agrobacterium radiobacter Agrobacterium vitis Agrobacterium sp. H13-3 Rhizobium etli CFN 42 Rhizobium etli CIAT 652 Rhizobium etli bv. mimosae Rhizobium leguminosarum bv. viciae 3841 Rhizobium leguminosarum bv. trifolii WSM2304 Rhizobium leguminosarum bv. trifolii WSM1325 Rhizobium leguminosarum bv. trifolii WSM1689 Rhizobium leguminosarum bv. trifolii CB782 Rhizobium tropici Rhizobium sp. IRBG74 Rhizobium sp. LPU83 Neorhizobium galegae bv. officinalis bv. officinalis HAMBI 1141 Liberibacter crescens Brucella melitensis bv. 1 16M Brucella melitensis ATCC 23457 Brucella melitensis M28 Brucella melitensis M5-90 Brucella melitensis NI Brucella abortus 2308 Brucella abortus 9-941 Brucella abortus S19 Brucella abortus A13334 Brucella suis 1330 Brucella suis 1330 Brucella suis ATCC 23445 Brucella suis VBI22 Brucella suis bv. 1 Brucella ovis Brucella canis ATCC 23365 Brucella canis HSK A52141 Brucella canis Oliveri Brucella microti Brucella pinnipedialis B2/94 Brucella ceti TE10759-12 Brucella ceti TE28753-12 Ochrobactrum anthropi ATCC 49188 Bradyrhizobium diazoefficiens USDA 110 Bradyrhizobium japonicum USDA 6 Bradyrhizobium sp. ORS 278 Bradyrhizobium sp. BTAi1 Bradyrhizobium sp. S23321 Bradyrhizobium oligotrophicum Rhodopseudomonas palustris CGA009 Rhodopseudomonas palustris HaA2 Rhodopseudomonas palustris BisB18 Rhodopseudomonas palustris BisB5 Rhodopseudomonas palustris BisA53 Rhodopseudomonas palustris TIE-1 Rhodopseudomonas palustris DX-1 Nitrobacter winogradskyi Nitrobacter hamburgensis Oligotropha carboxidovorans OM5 (Mississippi) Oligotropha carboxidovorans OM5 (Goettingen) Oligotropha carboxidovorans OM4 Xanthobacter autotrophicus Azorhizobium caulinodans Starkeya novella Methylobacterium extorquens PA1 Methylobacterium extorquens AM1 Methylobacterium extorquens DM4 Methylobacterium extorquens CM4 Methylobacterium radiotolerans Methylobacterium sp. 4-46 Methylobacterium populi Methylobacterium nodulans Beijerinckia indica Hyphomicrobium sp. MC1 Hyphomicrobium nitrativorans Rhodomicrobium vannielii Pelagibacterium halotolerans Methylocystis sp. SC2 Caulobacter crescentus CB15 Caulobacter crescentus NA1000 Caulobacter sp. K31 Caulobacter segnis Phenylobacterium zucineum Brevundimonas subvibrioides Asticcacaulis excentricus Ruegeria pomeroyi Ruegeria sp. TM1040 Rhodobacter sphaeroides 2.4.1 Rhodobacter sphaeroides ATCC 17029 Rhodobacter sphaeroides ATCC 17025 Rhodobacter sphaeroides KD131 Rhodobacter capsulatus Jannaschia sp. CCS1 Roseobacter denitrificans Roseobacter litoralis Paracoccus denitrificans Paracoccus aminophilus Dinoroseobacter shibae Pseudovibrio sp. FO-BEG1 Phaeobacter inhibens Phaeobacter gallaeciensis 2.10 Phaeobacter gallaeciensis DSM 26640 Octadecabacter antarcticus Octadecabacter arcticus Roseibacterium elongatum Planktomarina temperata Maricaulis maris Hyphomonas neptunium Hirschia baltica Zymomonas mobilis subsp. mobilis ZM4 Zymomonas mobilis subsp. mobilis NCIMB 11163 Zymomonas mobilis subsp. mobilis ATCC 29191 Zymomonas mobilis subsp. mobilis CP4 = NRRL B-14023 Zymomonas mobilis subsp. mobilis NRRL B-12526 Zymomonas mobilis subsp. pomaceae ATCC 29192 Novosphingobium aromaticivorans Novosphingobium sp. PP1Y Sphingopyxis alaskensis Sphingomonas wittichii Sphingomonas sp. MM-1 Sphingobium japonicum Sphingobium chlorophenolicum Sphingobium sp. SYK-6 Gluconobacter oxydans 621H Gluconobacter oxydans H24 Granulibacter bethesdensis CGDNIH1 Acidiphilium cryptum Acidiphilium multivorum Gluconacetobacter diazotrophicus PAl 5 (Brazil) Gluconacetobacter diazotrophicus PAl 5 (JGI) Gluconacetobacter medellinensis NBRC 3288 Gluconacetobacter xylinus E25 Acetobacter pasteurianus IFO 3283-01 Acetobacter pasteurianus IFO 3283-01-42C Acetobacter pasteurianus IFO 3283-03 Acetobacter pasteurianus IFO 3283-07 Acetobacter pasteurianus IFO 3283-12 Acetobacter pasteurianus IFO 3283-22 Acetobacter pasteurianus IFO 3283-26 Acetobacter pasteurianus IFO 3283-32 Acetobacter pasteurianus 386B Rhodospirillum rubrum ATCC 11170 Rhodospirillum rubrum F11 Rhodospirillum centenum Rhodospirillum photometricum Magnetospirillum magneticum Azospirillum sp. B510 Azospirillum lipoferum Azospirillum brasilense Tistrella mobilis Endolissoclinum patella Polymorphum gilvum Candidatus Pelagibacter ubique Alpha proteobacterium HIMB59 Alpha proteobacterium HIMB5 Candidatus Puniceispirillum marinum Bacillus subtilis subsp. subtilis 168 Bacillus subtilis subsp. subtilis RO-NN-1 Bacillus subtilis subsp. subtilis BSP1 Bacillus subtilis subsp. subtilis 6051-HGW Bacillus subtilis subsp. subtilis BAB-1 Bacillus subtilis subsp. spizizenii W23 Bacillus subtilis subsp. spizizenii TU-B-10 Bacillus subtilis subsp. natto BEST195 Bacillus subtilis BSn5 Bacillus subtilis QB928 Bacillus subtilis BEST7613 Bacillus subtilis XF-1 Bacillus subtilis PY79 Bacillus licheniformis ATCC 14580 Bacillus licheniformis DSM 13 = ATCC 14580 Bacillus licheniformis 9945A Bacillus amyloliquefaciens DSM 7 Bacillus amyloliquefaciens FZB42 Bacillus amyloliquefaciens subsp. plantarum CAU B946 Bacillus amyloliquefaciens subsp. plantarum YAU B9601-Y2 Bacillus amyloliquefaciens subsp. plantarum AS43.3 Bacillus amyloliquefaciens subsp. plantarum UCMB5036 Bacillus amyloliquefaciens subsp. plantarum UCMB5033 Bacillus amyloliquefaciens subsp. plantarum UCMB5113 Bacillus amyloliquefaciens subsp. plantarum NAU-B3 Bacillus amyloliquefaciens subsp. plantarum TrigoCor1448 Bacillus amyloliquefaciens TA208 Bacillus amyloliquefaciens LL3 Bacillus amyloliquefaciens XH7 Bacillus amyloliquefaciens Y2 Bacillus amyloliquefaciens IT-45 Bacillus amyloliquefaciens CC178 Bacillus amyloliquefaciens LFB112 Bacillus atrophaeus Bacillus halodurans Bacillus anthracis Ames Bacillus anthracis Ames 0581 Bacillus anthracis Sterne Bacillus anthracis CDC 684 Bacillus anthracis A0248 Bacillus anthracis H9401 Bacillus anthracis A16 Bacillus anthracis A16R Bacillus anthracis SVA11 Bacillus cereus biovar anthracis CI Bacillus cereus ATCC 14579 Bacillus cereus ATCC 10987 Bacillus cereus ZK Bacillus cereus AH187 Bacillus cereus B4264 Bacillus cereus AH820 Bacillus cereus G9842 Bacillus cereus Q1 Bacillus cereus 03BB102 Bacillus cereus NC7401 Bacillus cereus F837/76 Bacillus cereus FRI-35 Bacillus cytotoxicus Bacillus thuringiensis 97-27 Bacillus thuringiensis Al Hakam Bacillus thuringiensis BMB171 Bacillus thuringiensis serovar kurstaki HD73 Bacillus thuringiensis serovar chinensis CT-43 Bacillus thuringiensis serovar finitimus YBT-020 Bacillus thuringiensis MC28 Bacillus thuringiensis Bt407 Bacillus thuringiensis HD-771 Bacillus thuringiensis HD-789 Bacillus thuringiensis serovar thuringiensis IS5056 Bacillus thuringiensis YBT-1518 Bacillus weihenstephanensis Bacillus toyonensis Bacillus clausii Bacillus pumilus SAFR-032 Bacillus pumilus MTCC B6033 Bacillus pseudofirmus Bacillus megaterium QM B1551 Bacillus megaterium DSM 319 Bacillus megaterium WSH-002 Bacillus cellulosilyticus Bacillus coagulans 2-6 Bacillus coagulans 36D1 Bacillus sp. JS Bacillus sp. 1NLA3E Bacillus infantis Bacillus lehensis Bacillus methanolicus Oceanobacillus iheyensis Geobacillus kaustophilus Geobacillus thermodenitrificans Geobacillus thermoglucosidasius Geobacillus thermoleovorans Geobacillus sp. WCH70 Geobacillus sp. Y412MC61 Geobacillus sp. Y412MC52 Geobacillus sp. C56-T3 Geobacillus sp. Y4.1MC1 Geobacillus sp. GHH01 Geobacillus sp. JF8 Anoxybacillus flavithermus Amphibacillus xylanus Lysinibacillus sphaericus Lysinibacillus sp. GY32 Halobacillus halophilus Terribacillus aidingensis Virgibacillus sp. SK37 Bacillus selenitireducens Staphylococcus aureus subsp. aureus N315 (MRSA/VSSA) Staphylococcus aureus subsp. aureus Mu50 (MRSA/VISA) Staphylococcus aureus subsp. aureus Mu3 (MRSA/hetero-VISA) Staphylococcus aureus subsp. aureus JH1 (MRSA/VSSA) Staphylococcus aureus subsp. aureus JH9 (MRSA/VRSA) Staphylococcus aureus subsp. aureus MW2 (CA-MRSA) Staphylococcus aureus subsp. aureus MSSA476 (MSSA) Staphylococcus aureus subsp. aureus MRSA252 (MRSA) Staphylococcus aureus subsp. aureus COL (MRSA) Staphylococcus aureus subsp. aureus USA300\_TCH1516 (CA-MSSA) Staphylococcus aureus subsp. aureus USA300\_FPR3757 (CA-MRSA) Staphylococcus aureus subsp. aureus NCTC8325 Staphylococcus aureus subsp. aureus Newman Staphylococcus aureus subsp. aureus ED98 Staphylococcus aureus subsp. aureus M013 (CA-MRSA) Staphylococcus aureus subsp. aureus VC40 Staphylococcus aureus subsp. aureus MSHR1132 Staphylococcus aureus subsp. aureus ED133 Staphylococcus aureus subsp. aureus JKD6159 Staphylococcus aureus subsp. aureus JKD6008 (MRSA/VISA) Staphylococcus aureus subsp. aureus ECT-R 2 Staphylococcus aureus subsp. aureus T0131 (MRSA) Staphylococcus aureus subsp. aureus TCH60 Staphylococcus aureus subsp. aureus 11819-97 (CA-MRSA) Staphylococcus aureus subsp. aureus 71193 Staphylococcus aureus subsp. aureus HO 5096 0412 Staphylococcus aureus subsp. aureus TW20 (MRSA) Staphylococcus aureus subsp. aureus ST398 (MRSA) Staphylococcus aureus subsp. aureus LGA251 Staphylococcus aureus subsp. aureus 55/2053 Staphylococcus aureus subsp. aureus 6850 (MSSA) Staphylococcus aureus subsp. aureus CN1 (CA-MRSA) Staphylococcus aureus subsp. aureus SA40 (CA-MRSA) Staphylococcus aureus subsp. aureus SA957 (CA-MRSA) Staphylococcus aureus subsp. aureus Z172 (MRSA/VISA) Staphylococcus aureus RF122 Staphylococcus aureus 04-02981 Staphylococcus aureus 08BA02176 Staphylococcus aureus M1 Staphylococcus aureus CA-347 Staphylococcus aureus Bmb9393 Staphylococcus aureus USA300-ISMMS1 Staphylococcus aureus ST228/10388 Staphylococcus aureus ST228/10497 Staphylococcus aureus ST228/15532 Staphylococcus aureus ST228/16035 Staphylococcus aureus ST228/18412 Staphylococcus aureus ST228/16125 Staphylococcus aureus ST228/18341 Staphylococcus aureus ST228/18583 Staphylococcus saprophyticus Staphylococcus lugdunensis HKU09-01 Staphylococcus lugdunensis N920143 Staphylococcus pseudintermedius HKU10-03 Staphylococcus pseudintermedius ED99 Exiguobacterium sibiricum Exiguobacterium sp. AT1b Exiguobacterium antarcticum Exiguobacterium sp. MH3 Brevibacillus brevis Paenibacillus sp. JDR-2 Paenibacillus sp. Y412MC10 Paenibacillus polymyxa E681 Paenibacillus polymyxa SC2 Paenibacillus polymyxa M1 Paenibacillus polymyxa CR1 Paenibacillus polymyxa SQR-21 Paenibacillus mucilaginosus KNP414 Paenibacillus mucilaginosus 3016 Paenibacillus mucilaginosus K02 Paenibacillus terrae Paenibacillus larvae Paenibacillus sabinae Lactococcus lactis subsp. cremoris MG1363 Lactococcus lactis subsp. cremoris A76 Lactococcus lactis subsp. cremoris NZ9000 Lactococcus garvieae Lg2 Streptococcus pyogenes SF370 (serotype M1) Streptococcus pyogenes MGAS5005 (serotype M1) Streptococcus pyogenes M1 476 (serotype M1) Streptococcus pyogenes MGAS8232 (serotype M18) Streptococcus pyogenes MGAS315 (serotype M3) Streptococcus pyogenes SSI-1 (serotype M3) Streptococcus pyogenes MGAS10270 (serotype M2) Streptococcus pyogenes MGAS10750 (serotype M4) Streptococcus pyogenes MGAS2096 (serotype M12) Streptococcus pyogenes MGAS9429 (serotype M12) Streptococcus pyogenes Manfredo (serotype M5) Streptococcus pyogenes MGAS10394 (serotype M6) Streptococcus pyogenes MGAS6180 (serotype M28) Streptococcus pyogenes MGAS15252 (serotype M59) Streptococcus pyogenes NZ131 (serotype M49) Streptococcus pyogenes Alab49 (serotype M53) Streptococcus pyogenes MGAS1882 Streptococcus pyogenes A20 Streptococcus pyogenes HSC5 Streptococcus pneumoniae TIGR4 (virulent serotype 4) Streptococcus pneumoniae D39 (virulent serotype 2) Streptococcus pneumoniae R6 (avirulent) Streptococcus pneumoniae CGSP14 (serotype 14) Streptococcus pneumoniae G54 (serotype 19F) Streptococcus pneumoniae ATCC 700669 (serotype 23F ST81 lineage) Streptococcus pneumoniae Hungary19A 6 Streptococcus pneumoniae 70585 Streptococcus pneumoniae JJA Streptococcus pneumoniae P1031 Streptococcus pneumoniae Taiwan19F-14 Streptococcus pneumoniae TCH8431/19A Streptococcus pneumoniae 670-6B Streptococcus pneumoniae AP200 Streptococcus pneumoniae INV104 Streptococcus pneumoniae INV200 Streptococcus pneumoniae OXC141 Streptococcus pneumoniae ST556 Streptococcus pneumoniae SPNA45 Streptococcus pneumoniae gamPNI0373 Streptococcus pneumoniae SPN034156 Streptococcus pneumoniae SPN034183 Streptococcus pneumoniae SPN994038 Streptococcus pneumoniae SPN994039 Streptococcus pneumoniae A026 Streptococcus agalactiae 2603 (serotype V) Streptococcus agalactiae NEM316 (serotype III) Streptococcus agalactiae A909 (serotype Ia) Streptococcus agalactiae GD201008-001 (serotype Ia) Streptococcus agalactiae SA20-06 Streptococcus agalactiae 2-22 (serotype Ib) Streptococcus agalactiae 09mas018883 Streptococcus agalactiae ILRI005 Streptococcus agalactiae ILRI112 Streptococcus agalactiae 138P Streptococcus mutans UA159 Streptococcus mutans NN2025 Streptococcus mutans LJ23 Streptococcus mutans GS-5 Streptococcus sanguinis Streptococcus suis 05ZYH33 Streptococcus suis 98HAH33 Streptococcus suis BM407 Streptococcus suis P1/7 Streptococcus suis SC84 Streptococcus suis ST3 Streptococcus suis A7 Streptococcus suis D12 Streptococcus suis D9 Streptococcus suis GZ1 Streptococcus suis JS14 Streptococcus suis SS12 Streptococcus suis ST1 Streptococcus suis S735 Streptococcus suis SC070731 Streptococcus suis TL13 Streptococcus suis T15 Streptococcus suis YB51 Streptococcus equi subsp. zooepidemicus H70 Streptococcus equi subsp. zooepidemicus MGCS10565 Streptococcus equi subsp. zooepidemicus ATCC 35246 Streptococcus equi subsp. equi 4047 Streptococcus uberis Streptococcus dysgalactiae subsp. equisimilis GGS\_124 Streptococcus dysgalactiae subsp. equisimilis ATCC 12394 Streptococcus dysgalactiae subsp. equisimilis RE378 Streptococcus dysgalactiae subsp. equisimilis AC-2713 Streptococcus dysgalactiae subsp. equisimilis 167 Streptococcus gallolyticus UCN34 Streptococcus gallolyticus subsp. gallolyticus ATCC BAA-2069 Streptococcus gallolyticus subsp. gallolyticus ATCC 43143 Streptococcus mitis Streptococcus parauberis Streptococcus pasteurianus Streptococcus intermedius JTH08 Streptococcus intermedius B196 Streptococcus intermedius C270 Streptococcus anginosus C238 Streptococcus constellatus subsp. pharyngis C1050 Streptococcus constellatus subsp. pharyngis C232 Streptococcus constellatus subsp. pharyngis C818 Streptococcus iniae Lactobacillus plantarum ZJ316 Lactobacillus plantarum 16 Lactobacillus casei ATCC 334 Lactobacillus casei BL23 Lactobacillus casei Zhang Lactobacillus casei BD-II Lactobacillus casei LC2W Lactobacillus casei W56 Lactobacillus casei LOCK919 Lactobacillus rhamnosus GG Lactobacillus rhamnosus GG Lactobacillus rhamnosus Lc 705 Lactobacillus rhamnosus ATCC 8530 Lactobacillus rhamnosus LOCK900 Lactobacillus rhamnosus LOCK908 Lactobacillus buchneri CD034 Lactobacillus paracasei subsp. paracasei 8700:2 Enterococcus faecalis V583 Enterococcus faecalis 62 Enterococcus faecalis OG1RF Enterococcus faecalis D32 Enterococcus faecalis Symbioflor 1 Enterococcus faecalis DENG1 Enterococcus faecium Aus0004 Enterococcus faecium Aus0085 Enterococcus faecium DO Enterococcus faecium NRRL B-2354 Enterococcus hirae Enterococcus sp. 7L76 Enterococcus casseliflavus Enterococcus mundtii Tetragenococcus halophilus Oenococcus oeni Leuconostoc mesenteroides subsp. mesenteroides ATCC 8293 Leuconostoc mesenteroides subsp. mesenteroides J18 Leuconostoc mesenteroides KFRI-MG Leuconostoc gasicomitatum Leuconostoc carnosum Leuconostoc gelidum Aerococcus urinae Carnobacterium sp. 17-4 Carnobacterium maltaromaticum Carnobacterium sp. WN1359 Clostridium perfringens 13 Clostridium perfringens ATCC 13124 Clostridium perfringens SM101 Clostridium tetani 12124569 Clostridium botulinum A ATCC 3502 Clostridium botulinum A ATCC 19397 Clostridium botulinum A Hall Clostridium botulinum A2 Clostridium botulinum A3 Loch Maree Clostridium botulinum B Eklund 17B Clostridium botulinum B1 Okra Clostridium botulinum Ba4 Clostridium botulinum BKT015925 Clostridium botulinum E3 Clostridium botulinum F Langeland Clostridium botulinum F 230613 Clostridium botulinum H04402 065 Clostridium beijerinckii Clostridium kluyveri DSM 555 Clostridium kluyveri NBRC 12016 Clostridium ljungdahlii Clostridium saccharoperbutylacetonicum Clostridium pasteurianum Clostridium saccharobutylicum Clostridium autoethanogenum Clostridium sp. M2/40 Candidatus Arthromitus sp. SFB-mouse-Japan Candidatus Arthromitus sp. SFB-mouse-Yit Candidatus Arthromitus sp. SFB-rat-Yit Ruminiclostridium thermocellum ATCC 27405 Ruminiclostridium thermocellum DSM 1313 Clostridium clariflavum Ruminococcus sp. SR1/5 Butyrivibrio proteoclasticus Butyrivibrio fibrisolvens Cellulosilyticum lentocellum Coprococcus catus Ruminococcus obeum Ruminococcus torques Lachnoclostridium phytofermentans Clostridium saccharolyticum WM1 Clostridium cf. saccharolyticum K10 Peptoclostridium difficile 630 Peptoclostridium difficile CD196 Peptoclostridium difficile R20291 Peptoclostridium difficile BI1 Symbiobacterium thermophilum Desulfitobacterium hafniense Y51 Desulfitobacterium hafniense DCB-2 Desulfitobacterium dehalogenans Desulfosporosinus orientis Finegoldia magna Anaerococcus prevotii Eubacterium limosum Acetobacterium woodii Oscillibacter valericigenes Thermaerobacter marianensis Butyrate-producing bacterium SM4/1 Butyrate-producing bacterium SSC/2 Thermoanaerobacter sp. X514 Thermoanaerobacter sp. X513 Thermoanaerobacter mathranii Thermoanaerobacter wiegelii Tepidanaerobacter acetatoxydans Re1 Thermoanaerobacterium thermosaccharolyticum M0795 Thermoanaerobacterium xylanolyticum Coprothermobacter proteolyticus Halothermothrix orenii Acetohalobium arabaticum Halobacteroides halobius Veillonella parvula Pelosinus sp. UFO1 Acidaminococcus fermentans Acidaminococcus intestini Erysipelothrix rhusiopathiae Fujisawa Erysipelothrix rhusiopathiae SY1027 Mycoplasma pneumoniae M129 Mycoplasma pneumoniae 309 Mycoplasma pneumoniae FH Mycoplasma pneumoniae M129-B7 Mycoplasma pulmonis Mycoplasma penetrans Mycoplasma hyopneumoniae 232 Mycoplasma hyopneumoniae J Mycoplasma hyopneumoniae 7448 Mycoplasma hyopneumoniae 168 Mycoplasma hyopneumoniae 168-L Mycoplasma hyopneumoniae 7422 Mycoplasma synoviae Mycoplasma agalactiae PG2 Mycoplasma agalactiae 5632 Mycoplasma conjunctivae Mycoplasma crocodyli Mycoplasma hyorhinis HUB-1 Mycoplasma hyorhinis GDL-1 Mycoplasma hyorhinis MCLD Mycoplasma hyorhinis SK76 Mycoplasma hyorhinis DBS 1050 Mycoplasma fermentans JER Mycoplasma fermentans M64 Mycoplasma fermentans PG18 Mycoplasma bovis PG45 Mycoplasma bovis Hubei-1 Mycoplasma bovis HB0801 Mycoplasma cynos Acholeplasma palmae Mycobacterium tuberculosis H37Rv Mycobacterium tuberculosis H37Rv Mycobacterium tuberculosis CDC1551 Mycobacterium tuberculosis H37Ra Mycobacterium tuberculosis F11 Mycobacterium tuberculosis KZN 1435 Mycobacterium tuberculosis KZN 4207 Mycobacterium tuberculosis KZN 605 Mycobacterium tuberculosis RGTB327 Mycobacterium tuberculosis RGTB423 Mycobacterium tuberculosis CCDC5079 Mycobacterium tuberculosis CCDC5079 Mycobacterium tuberculosis CCDC5180 Mycobacterium tuberculosis CTRI-2 Mycobacterium tuberculosis UT205 Mycobacterium tuberculosis Erdman = ATCC 35801 Mycobacterium tuberculosis Beijing/NITR203 Mycobacterium tuberculosis 7199-99 Mycobacterium tuberculosis CAS/NITR204 Mycobacterium tuberculosis EAI5/NITR206 Mycobacterium tuberculosis EAI5 Mycobacterium tuberculosis Haarlem Mycobacterium tuberculosis BT1 Mycobacterium tuberculosis BT2 Mycobacterium tuberculosis HKBS1 Mycobacterium bovis AF2122/97 Mycobacterium bovis BCG Pasteur 1173P2 Mycobacterium bovis BCG Tokyo 172 Mycobacterium bovis BCG Mexico Mycobacterium bovis BCG Korea 1168P Mycobacterium africanum Mycobacterium canettii CIPT 140010059 Mycobacterium canettii CIPT 140060008 Mycobacterium canettii CIPT 140070008 Mycobacterium canettii CIPT 140070010 Mycobacterium canettii CIPT 140070017 Mycobacterium avium subsp. paratuberculosis K-10 Mycobacterium avium subsp. paratuberculosis MAP4 Mycobacterium avium 104 Mycobacterium intracellulare MOTT-02 Mycobacterium intracellulare MOTT-64 Mycobacterium intracellulare ATCC 13950 Mycobacterium indicus pranii Mycobacterium yongonense Mycobacterium smegmatis MC2 155 Mycobacterium smegmatis MC2 155 Mycobacterium smegmatis JS623 Mycobacterium ulcerans Mycobacterium vanbaalenii Mycobacterium gilvum PYR-GCK Mycobacterium gilvum Spyr1 Mycobacterium abscessus ATCC 19977 Mycobacterium abscessus subsp. bolletii 50594 Mycobacterium abscessus subsp. bolletii GO 06 Mycobacterium sp. MCS Mycobacterium sp. KMS Mycobacterium sp. JLS Mycobacterium marinum Mycobacterium rhodesiae Mycobacterium sp. MOTT36Y Mycobacterium chubuense Mycobacterium liflandii Mycobacterium kansasii Mycobacterium neoaurum Amycolicicoccus subflavus Corynebacterium glutamicum ATCC 13032 (Kyowa Hakko) Corynebacterium glutamicum ATCC 13032 (Bielefeld) Corynebacterium glutamicum K051 Corynebacterium glutamicum R Corynebacterium glutamicum SCgG1 Corynebacterium glutamicum SCgG2 Corynebacterium glutamicum MB001 Corynebacterium efficiens Corynebacterium diphtheriae NCTC 13129 Corynebacterium diphtheriae 241 Corynebacterium diphtheriae INCA 402 Corynebacterium diphtheriae HC01 Corynebacterium diphtheriae HC02 Corynebacterium diphtheriae HC03 Corynebacterium diphtheriae HC04 Corynebacterium diphtheriae 31A Corynebacterium diphtheriae BH8 Corynebacterium diphtheriae C7 Corynebacterium diphtheriae CDCE 8392 Corynebacterium diphtheriae PW8 Corynebacterium diphtheriae VA01 Corynebacterium pseudotuberculosis FRC41 Corynebacterium pseudotuberculosis 3/99-5 Corynebacterium pseudotuberculosis 316 Corynebacterium pseudotuberculosis P54B96 Corynebacterium pseudotuberculosis 1002 Corynebacterium pseudotuberculosis I19 Corynebacterium pseudotuberculosis 267 Corynebacterium pseudotuberculosis 31 Corynebacterium pseudotuberculosis 1/06-A Corynebacterium pseudotuberculosis 42/02-A Corynebacterium pseudotuberculosis CIP 52.97 Corynebacterium pseudotuberculosis 258 Corynebacterium pseudotuberculosis Cp162 Corynebacterium ulcerans BR-AD22 Corynebacterium ulcerans 809 Corynebacterium ulcerans 0102 Corynebacterium variabile Corynebacterium halotolerans Corynebacterium callunae Corynebacterium terpenotabidum Corynebacterium maris Corynebacterium argentoratense Corynebacterium falsenii Corynebacterium casei Corynebacterium vitaeruminis Corynebacterium atypicum Nocardia farcinica Nocardia cyriacigeorgica Nocardia brasiliensis Nocardia nova Rhodococcus jostii Rhodococcus erythropolis PR4 Rhodococcus erythropolis CCM2595 Rhodococcus opacus B4 Rhodococcus opacus PD630 Rhodococcus equi Rhodococcus pyridinivorans Gordonia bronchialis Gordonia polyisoprenivorans Gordonia sp. KTR9 Tsukamurella paurometabola Segniliparus rotundus Streptomyces coelicolor Streptomyces avermitilis Streptomyces griseus Streptomyces scabiei Streptomyces sp. SirexAA-E Streptomyces violaceusniger Streptomyces cattleya NRRL 8057 Streptomyces cattleya NRRL 8057 = DSM 46488 Streptomyces pratensis Streptomyces bingchenggensis Streptomyces hygroscopicus subsp. jinggangensis 5008 Streptomyces hygroscopicus subsp. jinggangensis TL01 Streptomyces venezuelae Streptomyces davawensis Streptomyces albus Streptomyces sp. PAMC26508 Streptomyces fulvissimus Streptomyces collinus Streptomyces rapamycinicus Streptomyces albulus Streptomyces lividans Kitasatospora setae Tropheryma whipplei Twist Tropheryma whipplei TW08/27 Leifsonia xyli subsp. xyli CTCB07 Leifsonia xyli subsp. cynodontis DSM 46306 Clavibacter michiganensis subsp. michiganensis Clavibacter michiganensis subsp. sepedonicus Clavibacter michiganensis subsp. nebraskensis Microbacterium testaceum Arthrobacter sp. FB24 Arthrobacter aurescens Arthrobacter chlorophenolicus Arthrobacter arilaitensis Arthrobacter phenanthrenivorans Arthrobacter sp. Rue61a Renibacterium salmoninarum Kocuria rhizophila Micrococcus luteus Beutenbergia cavernae Brachybacterium faecium Jonesia denitrificans Kytococcus sedentarius Isoptericola variabilis Sanguibacter keddieii Cellulomonas flavigena Cellulomonas fimi Cellulomonas gilvus Intrasporangium calvum Propionibacterium acnes KPA171202 Propionibacterium acnes SK137 Propionibacterium acnes TypeIA2 P.acn17 Propionibacterium acnes TypeIA2 P.acn31 Propionibacterium acnes TypeIA2 P.acn33 Propionibacterium acnes 266 Propionibacterium acnes ATCC 11828 Propionibacterium acnes 6609 Propionibacterium acnes C1 Propionibacterium acnes HL096PA1 Propionibacterium propionicum Propionibacterium avidum Microlunatus phosphovorus Nocardioides sp. JS614 Kribbella flavida Thermobifida fusca Nocardiopsis dassonvillei Nocardiopsis alba Thermomonospora curvata Streptosporangium roseum Frankia sp. CcI3 Frankia sp. EAN1pec Frankia sp. EuI1c Frankia alni Frankia symbiont Acidothermus cellulolyticus Geodermatophilus obscurus Blastococcus saxobsidens Modestobacter marinus Kineococcus radiotolerans Saccharopolyspora erythraea Saccharomonospora viridis Thermobispora bispora Amycolatopsis mediterranei U32 Amycolatopsis mediterranei S699 Amycolatopsis mediterranei S699 Amycolatopsis mediterranei RB Amycolatopsis orientalis Amycolatopsis japonica Amycolatopsis methanolica Pseudonocardia dioxanivorans Actinosynnema mirum Saccharothrix espanaensis Kutzneria albida Salinispora tropica Salinispora arenicola Micromonospora aurantiaca Micromonospora sp. L5 Verrucosispora maris Actinoplanes missouriensis Actinoplanes sp. SE50/110 Actinoplanes sp. N902-109 Actinoplanes friuliensis Catenulispora acidiphila Stackebrandtia nassauensis Actinobaculum schaalii Bifidobacterium longum subsp. infantis ATCC 15697 (JGI) Bifidobacterium longum subsp. infantis ATCC 15697 (Tokyo) Bifidobacterium longum subsp. longum JDM301 Bifidobacterium longum subsp. longum BBMN68 Bifidobacterium bifidum S17 Bifidobacterium bifidum PRL2010 Bifidobacterium bifidum BGN4 Bifidobacterium breve ACS-071-V-Sch8b Bifidobacterium breve UCC2003 Bifidobacterium breve 12L Bifidobacterium breve 689b Bifidobacterium breve JCM 7017 Bifidobacterium breve JCM 7019 Bifidobacterium breve NCFB 2258 Bifidobacterium breve S27 Rubrobacter xylanophilus Rubrobacter radiotolerans Conexibacter woesei Ilumatobacter coccineus Atopobium parvulum Eggerthella sp. YY7918 Coriobacterium glomerans Gordonibacter pamelaeae Parachlamydia acanthamoebae Opitutus terrae Coraliomargarita akajimensis Treponema brennaborense Treponema azotonutricium Spirochaeta smaragdinae Spirochaeta africana Spirochaeta sp. L21-RPul-D2 Sphaerochaeta globosa Sphaerochaeta pleomorpha Leptospira interrogans serovar Lai 56601 Leptospira interrogans serovar Lai IPAV Leptospira interrogans serovar Copenhageni Leptospira borgpetersenii JB197 Leptospira borgpetersenii L550 Leptospira biflexa serovar Patoc Patoc 1 (Paris) Leptospira biflexa serovar Patoc Patoc 1 (Ames) Turneriella parva Brachyspira hyodysenteriae Brachyspira murdochii Brachyspira pilosicoli 95/1000 Brachyspira pilosicoli B2904 Brachyspira pilosicoli P43/6/78 Brachyspira pilosicoli WesB Brachyspira intermedia Candidatus Koribacter versatilis Acidobacterium capsulatum Granulicella tundricola Granulicella mallensis Terriglobus saanensis Terriglobus roseus Candidatus Solibacter usitatus Candidatus Chloracidobacterium thermophilum B Fibrobacter succinogenes Fibrobacter succinogenes Sebaldella termitidis Streptobacillus moniliformis Gemmatimonas aurantiaca Gemmatimonadetes bacterium KBS708 Rhodopirellula baltica Pirellula staleyi Planctomyces limnophilus Planctomyces brasiliensis Isosphaera pallida Singulisphaera acidiphila Phycisphaera mikurensis Synechocystis sp. PCC 6803 Synechocystis sp. PCC 6803 Synechocystis sp. PCC 6803 GT-S Synechocystis sp. PCC 6803 GT-I Synechocystis sp. PCC 6803 PCC-N Synechocystis sp. PCC 6803 PCC-P Synechococcus sp. WH8102 Synechococcus elongatus PCC6301 Synechococcus elongatus PCC7942 Synechococcus sp. CC9605 Synechococcus sp. CC9902 Synechococcus sp. CC9311 Synechococcus sp. RCC307 Synechococcus sp. WH7803 Synechococcus sp. PCC 7502 Microcystis aeruginosa Cyanothece sp. ATCC 51142 Cyanothece sp. PCC 8801 Cyanothece sp. PCC 7424 Cyanothece sp. PCC 8802 Cyanothece sp. PCC 7822 Acaryochloris marina Cyanobium gracile Cyanobacterium aponinum Cyanobacterium stanieri Dactylococcopsis salina Halothece sp. PCC 7418 Gloeocapsa sp. PCC 7428 Chamaesiphon minutus Cyanobacterium UCYN-A Trichodesmium erythraeum Oscillatoria acuminata Oscillatoria nigro-viridis Pseudanabaena sp. PCC 7367 Crinalium epipsammum Microcoleus sp. PCC 7113 Arthrospira platensis Gloeobacter violaceus Gloeobacter kilaueensis Nostoc sp. PCC 7120 Nostoc punctiforme Nostoc sp. PCC 7107 Nostoc sp. PCC 7524 Anabaena variabilis Anabaena sp. 90 Anabaena cylindrica Anabaena azollae 0708 Cylindrospermum stagnale Calothrix sp. PCC 7507 Calothrix sp. PCC 6303 Rivularia sp. PCC 7116 Prochlorococcus marinus SS120 Prochlorococcus marinus MED4 Prochlorococcus marinus MIT 9313 Prochlorococcus marinus NATL2A Prochlorococcus marinus MIT9312 Prochlorococcus marinus MIT 9303 Prochlorococcus marinus MIT 9301 Prochlorococcus marinus MIT 9215 Prochlorococcus marinus MIT 9211 Prochlorococcus marinus NATL1A Chroococcidiopsis thermalis Pleurocapsa sp. PCC 7327 Stanieria cyanosphaera Bacteroides thetaiotaomicron Bacteroides salanitronis Bacteroides xylanisolvens Bacteroides sp. CF50 Paludibacter propionicigenes Odoribacter splanchnicus Barnesiella viscericola Prevotella dentalis Alistipes shahii Draconibacterium orientale Salinibacter ruber DSM 13855 Salinibacter ruber M8 Rhodothermus marinus DSM 4252 Rhodothermus marinus SG0.5JP17-172 Chitinophaga pinensis Niastella koreensis Pedobacter heparinus Pedobacter saltans Sphingobacterium sp. 21 Solitalea canadensis Haliscomenobacter hydrossis Saprospira grandis Cyclobacterium marinum Belliella baltica Echinicola vietnamensis Cytophaga hutchinsonii Dyadobacter fermentans Spirosoma linguale Leadbetterella byssophila Runella slithyformis Flexibacter litoralis Emticicia oligotrophica Fibrella aestuarina Hymenobacter swuensis Hymenobacter sp. APR13 Marivirga tractuosa Gramella forsetii Flavobacterium johnsoniae Flavobacterium psychrophilum JIP02/86 Flavobacterium branchiophilum Flavobacterium columnare Flavobacterium indicum Robiginitalea biformata Zunongwangia profunda Croceibacter atlanticus Riemerella anatipestifer ATCC 11845 = DSM 15868 Riemerella anatipestifer ATCC 11845 = DSM 15868 Riemerella anatipestifer RA-GD Riemerella anatipestifer RA-CH-1 Riemerella anatipestifer RA-CH-2 Maribacter sp. HTCC2170 Cellulophaga algicola Cellulophaga lytica Weeksella virosa Krokinobacter sp. 4H-3-7-5 Lacinutrix sp. 5H-3-7-4 Zobellia galactanivorans Muricauda ruestringensis Aequorivita sublithincola Psychroflexus torquis Nonlabens dokdonensis Polaribacter sp. MED152 Flavobacteriaceae bacterium Fluviicola taffensis Owenweeksia hongkongensis Chlorobium tepidum Chlorobaculum parvum Chlorobium phaeobacteroides BS1 Chlorobium limicola Prosthecochloris aestuarii Ignavibacterium album Melioribacter roseus Roseiflexus sp. RS-1 Roseiflexus castenholzii Chloroflexus aurantiacus Chloroflexus aggregans Chloroflexus sp. Y-400-fl Herpetosiphon aurantiacus Thermomicrobium roseum Sphaerobacter thermophilus Caldilinea aerophila Deinococcus radiodurans Deinococcus deserti Deinococcus maricopensis Deinococcus proteolyticus Deinococcus peraridilitoris Truepera radiovictrix Thermus thermophilus SG0.5JP17-16 Meiothermus silvanus Marinithermus hydrothermalis Chthonomonas calidirosea Fimbriimonas ginsengisoli Dictyoglomus thermophilum Candidatus Nitrospira defluvii Thermobaculum terrenum Candidatus Methylomirabilis oxyfera Methanosarcina acetivorans Methanosarcina barkeri Methanosarcina mazei Go1 Methanosarcina mazei Tuc01 Methanococcoides burtonii Methanosalsum zhilinae Methanomethylovorans hollandica Halobacterium sp. NRC-1 Halobacterium salinarum Haloarcula marismortui Haloarcula hispanica ATCC 33960 Haloarcula hispanica N601 Haloquadratum walsbyi DSM 16790 Haloquadratum walsbyi C23 Natronomonas pharaonis Natronomonas moolapensis Halorhabdus utahensis Halorhabdus tiamatea Halomicrobium mukohataei Haloterrigena turkmenica Natrialba magadii Haloferax volcanii Haloferax mediterranei Halalkalicoccus jeotgali Halogeometricum borinquense Halopiger xanaduensis Natrinema sp. J7-2 Natrinema pellirubrum Natronobacterium gregoryi Halovivax ruber Natronococcus occultus Salinarchaeum sp. Harcht-Bsk1 Halostagnicola larsenii Ignisphaera aggregans Sulfolobus solfataricus P2 Sulfolobus islandicus L.S.2.15 Sulfolobus islandicus M.14.25 Sulfolobus islandicus M.16.27 Sulfolobus islandicus M.16.4 Sulfolobus islandicus Y.N.15.51 Sulfolobus islandicus L.D.8.5 Sulfolobus islandicus HVE10/4 Pyrobaculum calidifontis Thermofilum pendens Thermofilum sp. 1910b Candidatus Caldiarchaeum subterraneum Halophilic archaeon | 184% 150% 122% 100% 82% 67% 55% |
