# Supplementary material for: Global analysis of lysine acetylation in strawberry leaves
Source: Front Plant Sci. 2015 Sep 15;6:739. doi: 10.3389/fpls.2015.00739 (PMC4569977; doi:10.3389/fpls.2015.00739)
Supplement: Supplementary file 5 [file DataSheet1.ZIP › Butanoate metabolism - Fragaria vesca (woodland strawberry).html]

KEGG PATHWAY: Butanoate metabolism - Fragaria vesca (woodland strawberry)


|  |  |
| --- | --- |
| **Butanoate metabolism - Fragaria vesca (woodland strawberry)** |  |

[
Pathway menu
| Organism menu
| Pathway entry
| Download KGML
| User data mapping
]

|  |  |
| --- | --- |
| Reference pathway Reference pathway (KO) Reference pathway (EC) Reference pathway (Reaction) -----< Set personalized menu >----- -----< Sort below by alphabet >----- Homo sapiens (human) Homo sapiens (human) + Disease/drug Pan troglodytes (chimpanzee) Pan paniscus (bonobo) Gorilla gorilla gorilla (western lowland gorilla) Pongo abelii (Sumatran orangutan) Macaca mulatta (rhesus monkey) Macaca fascicularis (crab-eating macaque) Mus musculus (mouse) Rattus norvegicus (rat) Cricetulus griseus (Chinese hamster) Heterocephalus glaber (naked mole rat) Tupaia chinensis (Chinese tree shrew) Canis familiaris (dog) Ailuropoda melanoleuca (giant panda) Felis catus (domestic cat) Panthera tigris altaica (Amur tiger) Bos taurus (cow) Bos mutus (wild yak) Pantholops hodgsonii (chiru) Capra hircus (goat) Ovis aries (sheep) Sus scrofa (pig) Camelus ferus (Wild Bactrian camel) Balaenoptera acutorostrata scammoni (minke whale) Lipotes vexillifer (Yangtze River dolphin) Equus caballus (horse) Myotis brandtii (Brandt's bat) Myotis davidii Pteropus alecto (black flying fox) Monodelphis domestica (opossum) Sarcophilus harrisii (Tasmanian devil) Ornithorhynchus anatinus (platypus) Gallus gallus (chicken) Meleagris gallopavo (turkey) Taeniopygia guttata (zebra finch) Ficedula albicollis (collared flycatcher) Pseudopodoces humilis (Tibetan ground-tit) Anas platyrhynchos (mallard) Falco peregrinus (peregrine falcon) Falco cherrug (Saker falcon) Columba livia (rock pigeon) Alligator sinensis (Chinese alligator) Alligator mississippiensis (American alligator) Pelodiscus sinensis (Chinese soft-shelled turtle) Chelonia mydas (green sea turtle) Anolis carolinensis (green anole) Python bivittatus (Burmese python) Xenopus laevis (African clawed frog) Xenopus tropicalis (western clawed frog) Danio rerio (zebrafish) Takifugu rubripes (torafugu) Maylandia zebra (zebra mbuna) Oryzias latipes (Japanese medaka) Xiphophorus maculatus (southern platyfish) Latimeria chalumnae (coelacanth) Callorhinchus milii (elephant shark) Branchiostoma floridae (Florida lancelet) Ciona intestinalis (sea squirt) Strongylocentrotus purpuratus (purple sea urchin) Drosophila melanogaster (fruit fly) Drosophila pseudoobscura pseudoobscura Drosophila ananassae Drosophila erecta Drosophila persimilis Drosophila sechellia Drosophila simulans Drosophila willistoni Drosophila yakuba Drosophila grimshawi Drosophila mojavensis Drosophila virilis Anopheles gambiae (mosquito) Aedes aegypti (yellow fever mosquito) Culex quinquefasciatus (southern house mosquito) Apis mellifera (honey bee) Nasonia vitripennis (jewel wasp) Tribolium castaneum (red flour beetle) Bombyx mori (domestic silkworm) Acyrthosiphon pisum (pea aphid) Pediculus humanus corporis (human body louse) Ixodes scapularis (black-legged tick) Caenorhabditis elegans (nematode) Caenorhabditis briggsae Brugia malayi (filaria) Loa loa (eye worm) Trichinella spiralis Schistosoma mansoni Nematostella vectensis (sea anemone) Hydra vulgaris Trichoplax adhaerens Amphimedon queenslandica (sponge) Arabidopsis thaliana (thale cress) Arabidopsis lyrata (lyrate rockcress) Capsella rubella Eutrema salsugineum Citrus sinensis (Valencia orange) Citrus clementina (mandarin orange) Theobroma cacao (cacao) Glycine max (soybean) Phaseolus vulgaris (common bean) Medicago truncatula (barrel medic) Cicer arietinum (chickpea) Fragaria vesca (woodland strawberry) Prunus persica (peach) Prunus mume (Japanese apricot) Malus domestica (apple) Cucumis sativus (cucumber) Cucumis melo (muskmelon) Ricinus communis (castor bean) Populus trichocarpa (black cottonwood) Vitis vinifera (wine grape) Solanum lycopersicum (tomato) Solanum tuberosum (potato) Oryza sativa japonica (Japanese rice) (RefSeq) Oryza sativa japonica (Japanese rice) (RAPDB) Oryza brachyantha (malo sina) Brachypodium distachyon Sorghum bicolor (sorghum) Zea mays (maize) Setaria italica (foxtail millet) Amborella trichopoda Selaginella moellendorffii Physcomitrella patens subsp. patens Chlamydomonas reinhardtii Volvox carteri f. nagariensis Ostreococcus lucimarinus Ostreococcus tauri Bathycoccus prasinos Micromonas sp. RCC299 Micromonas pusilla Coccomyxa subellipsoidea Chlorella variabilis Cyanidioschyzon merolae Galdieria sulphuraria Chondrus crispus (carragheen) Saccharomyces cerevisiae (budding yeast) Ashbya gossypii (Eremothecium gossypii) Eremothecium cymbalariae Kluyveromyces lactis Lachancea thermotolerans Vanderwaltozyma polyspora Zygosaccharomyces rouxii Candida glabrata Naumovozyma castellii Naumovozyma dairenensis Tetrapisispora phaffii Tetrapisispora blattae Torulaspora delbrueckii Kazachstania africana Pichia pastoris Debaryomyces hansenii Scheffersomyces stipitis Meyerozyma guilliermondii Spathaspora passalidarum Lodderomyces elongisporus Candida albicans Candida tropicalis Candida orthopsilosis Candida dubliniensis Candida tenuis Yarrowia lipolytica Clavispora lusitaniae Neurospora crassa Sordaria macrospora Podospora anserina Thielavia terrestris Myceliophthora thermophila Chaetomium thermophilum Magnaporthe oryzae Togninia minima Fusarium graminearum Nectria haematococca Trichoderma reesei Metarhizium acridum Metarhizium anisopliae Cordyceps militaris Verticillium alfalfae Eutypa lata Sclerotinia sclerotiorum Botrytis cinerea Marssonina brunnea Aspergillus nidulans Aspergillus fumigatus Aspergillus oryzae Aspergillus niger Aspergillus flavus Aspergillus clavatus Neosartorya fischeri Penicillium chrysogenum Coccidioides immitis Coccidioides posadasii Paracoccidioides brasiliensis Uncinocarpus reesii Arthroderma benhamiae Trichophyton verrucosum Ajellomyces capsulatus Phaeosphaeria nodorum Pyrenophora teres Bipolaris zeicola Bipolaris sorokiniana Bipolaris oryzae Zymoseptoria tritici Pseudocercospora fijiensis Baudoinia compniacensis Neofusicoccum parvum Tuber melanosporum Schizosaccharomyces pombe (fission yeast) Cryptococcus neoformans JEC21 Cryptococcus neoformans B-3501A Cryptococcus gattii Tremella mesenterica Postia placenta Dichomitus squalens Stereum hirsutum Phanerochaete carnosa Punctularia strigosozonata Auricularia delicata Fomitiporia mediterranea Gloeophyllum trabeum Laccaria bicolor Moniliophthora perniciosa Moniliophthora roreri Coprinopsis cinerea Schizophyllum commune Agaricus bisporus var. burnettii JB137-S8 Agaricus bisporus var. bisporus H97 Coniophora puteana Serpula lacrymans Ustilago maydis Pseudozyma flocculosa Malassezia globosa Puccinia graminis Melampsora larici-populina Wallemia sebi Monosiga brevicollis Dictyostelium discoideum (cellular slime mold) Dictyostelium purpureum (cellular slime mold) Dictyostelium fasciculatum (cellular slime mold) Acanthamoeba castellanii Cryptosporidium hominis Toxoplasma gondii Tetrahymena thermophila Paramecium tetraurelia Phaeodactylum tricornutum Thalassiosira pseudonana Phytophthora infestans Nannochloropsis gaditana Emiliania huxleyi Guillardia theta Trypanosoma brucei Trypanosoma cruzi Leishmania major Leishmania infantum Leishmania donovani Leishmania mexicana Leishmania braziliensis Naegleria gruberi Trichomonas vaginalis Giardia lamblia Escherichia coli K-12 MG1655 Escherichia coli K-12 W3110 Escherichia coli K-12 DH10B Escherichia coli K-12 MC4100(MuLac) BW2952 Escherichia coli K-12 MDS42 Escherichia coli O157:H7 EDL933 (EHEC) Escherichia coli O157:H7 Sakai (EHEC) Escherichia coli O157:H7 EC4115 (EHEC) Escherichia coli O157:H7 TW14359 (EHEC) Escherichia coli O157:H7 Xuzhou21 (EHEC) Escherichia coli O26:H11 11368 (EHEC) Escherichia coli O111:H- 11128 (EHEC) Escherichia coli O103:H2 12009 (EHEC) Escherichia coli O127:H6 E2348/69 (EPEC) Escherichia coli O55:H7 CB9615 (EPEC) Escherichia coli O55:H7 RM12579 (EPEC) Escherichia coli O6:K2:H1 CFT073 (UPEC) Escherichia coli O6:K15:H31 536 (UPEC) Escherichia coli O18:K1:H7 UTI89 (UPEC) Escherichia coli APEC O1 (APEC) Escherichia coli O9 HS (commensal) Escherichia coli E24377A (ETEC) Escherichia coli SMS-3-5 (environmental) Escherichia coli O152:H28 SE11 (commensal) Escherichia coli O8 IAI1 (commensal) Escherichia coli O81 ED1a (commensal) Escherichia coli 55989 (EAEC) Escherichia coli O7:K1 IAI39 (ExPEC) Escherichia coli O7:K1 CE10 Escherichia coli O17:K52:H18 UMN026 (ExPEC) Escherichia coli O45:K1:H7 S88 (ExPEC) Escherichia coli O44:H18 042 (EAEC) Escherichia coli O83:H1 NRG 857C Escherichia coli O78:H11:K80 H10407 (ETEC) Escherichia coli O150:H5 SE15 (commensal) Escherichia coli O104:H4 2009EL-2071 Escherichia coli O104:H4 2009EL-2050 Escherichia coli O104:H4 2011C-3493 Escherichia coli ATCC 8739 Escherichia coli B REL606 Escherichia coli BL21-Gold(DE3)pLysS AG Escherichia coli KO11FL Escherichia coli KO11FL Escherichia coli ABU 83972 Escherichia coli DH1 Escherichia coli DH1 Escherichia coli IHE3034 Escherichia coli NA114 Escherichia coli UM146 Escherichia coli UMNK88 Escherichia coli W Escherichia coli W Escherichia coli clone D i14 Escherichia coli clone D i2 Escherichia coli P12b Escherichia coli BL21(DE3) Escherichia coli BL21(DE3) Escherichia coli LF82 Escherichia coli APEC O78 Escherichia coli LY180 Escherichia coli PMV-1 Escherichia coli JJ1886 Escherichia coli O145:H28 RM13514 (EHEC) Escherichia coli O145:H28 RM13516 (EHEC) Escherichia fergusonii Salmonella enterica subsp. enterica serovar Typhi CT18 Salmonella enterica subsp. enterica serovar Typhi Ty2 Salmonella enterica subsp. enterica serovar Typhi P-stx-12 Salmonella enterica subsp. enterica serovar Typhi Ty21a Salmonella enterica subsp. enterica serovar Typhimurium LT2 Salmonella enterica subsp. enterica serovar Typhimurium 14028S Salmonella enterica subsp. enterica serovar Typhimurium D23580 Salmonella enterica subsp. enterica serovar Typhimurium SL1344 Salmonella enterica subsp. enterica serovar Typhimurium T000240 Salmonella enterica subsp. enterica serovar Typhimurium UK-1 Salmonella enterica subsp. enterica serovar Typhimurium ST4/74 Salmonella enterica subsp. enterica serovar Typhimurium 798 Salmonella enterica subsp. enterica serovar Typhimurium U288 Salmonella enterica subsp. enterica serovar Typhimurium var. 5- CFSAN001921 Salmonella enterica subsp. enterica serovar Typhimurium 08-1736 Salmonella enterica subsp. enterica serovar Typhimurium DT2 Salmonella enterica subsp. enterica serovar Typhimurium DT104 Salmonella enterica subsp. enterica serovar Paratyphi A ATCC9150 Salmonella enterica subsp. enterica serovar Paratyphi A AKU12601 Salmonella enterica subsp. enterica serovar Paratyphi B Salmonella enterica subsp. enterica serovar Paratyphi C Salmonella enterica subsp. enterica serovar Choleraesuis Salmonella enterica subsp. enterica serovar Heidelberg SL476 Salmonella enterica subsp. enterica serovar Heidelberg B182 Salmonella enterica subsp. enterica Serovar Heidelberg CFSAN002069 Salmonella enterica subsp. enterica serovar Heidelberg 41578 Salmonella enterica subsp. enterica serovar Newport SL254 Salmonella enterica subsp. enterica serovar Newport USMARC-S3124.1 Salmonella enterica subsp. enterica serovar Schwarzengrund Salmonella enterica subsp. enterica serovar Agona SL483 Salmonella enterica subsp. enterica serovar Agona 24249 Salmonella enterica subsp. enterica serovar Dublin Salmonella enterica subsp. enterica serovar Gallinarum 287/91 Salmonella enterica subsp. enterica serovar Gallinarum/pullorum RKS5078 Salmonella enterica subsp. enterica serovar Gallinarum/pullorum CDC1983-67 Salmonella enterica subsp. enterica serovar Enteritidis Salmonella enterica subsp. enterica serovar Javiana Salmonella enterica subsp. enterica Serovar Cubana Salmonella enterica subsp. enterica serovar Bareilly Salmonella enterica subsp. enterica serovar Pullorum Salmonella enterica subsp. enterica serovar Bovismorbificans Salmonella enterica subsp. enterica serovar Thompson Salmonella enterica subsp. arizonae Salmonella bongori NCTC 12419 Salmonella bongori N268-08 Yersinia pestis CO92 (biovar Orientalis) Yersinia pestis KIM10+ (biovar Mediaevalis) Yersinia pestis Antiqua (biovar Antiqua) Yersinia pestis Nepal516 (biovar Antiqua) Yersinia pestis 91001 (biovar Microtus) Yersinia pestis Pestoides F Yersinia pestis Angola Yersinia pestis Z176003 Yersinia pestis A1122 Yersinia pestis D106004 Yersinia pestis D182038 Yersinia pestis biovar Medievalis Harbin 35 Yersinia pseudotuberculosis IP32953 (serotype I) Yersinia pseudotuberculosis IP31758 (serotype O:1b) Yersinia pseudotuberculosis YPIII Yersinia pseudotuberculosis PB1/+ Yersinia enterocolitica subsp. enterocolitica 8081 Yersinia enterocolitica subsp. palearctica 105.5R(r) Yersinia enterocolitica subsp. palearctica Y11 Yersinia enterocolitica LC20 Yersinia similis Shigella flexneri 301 (serotype 2a) Shigella flexneri 2457T (serotype 2a) Shigella flexneri 8401 (serotype 5b) Shigella flexneri 2002017 (serotype Fxv) Shigella sonnei Ss046 Shigella sonnei 53G Shigella boydii Sb227 Shigella boydii CDC 3083-94 Shigella dysenteriae Sd197 Shigella dysenteriae 1617 Pectobacterium atrosepticum SCRI1043 Pectobacterium atrosepticum JG10-08 Pectobacterium carotovorum subsp. carotovorum PC1 Pectobacterium carotovorum subsp. carotovorum PCC21 Pectobacterium wasabiae Pectobacterium sp. SCC3193 Erwinia tasmaniensis Erwinia pyrifoliae Ep1/96 Erwinia pyrifoliae DSM 12163 Erwinia amylovora CFBP1430 Erwinia amylovora ATCC 49946 Erwinia billingiae Erwinia sp. Ejp617 Photorhabdus luminescens Photorhabdus asymbiotica Sodalis glossinidius (Glossina spp.) Sodalis sp. HS1 Candidatus Sodalis pierantonius Enterobacter sp. 638 Enterobacter cloacae subsp. cloacae ATCC 13047 Enterobacter cloacae subsp. cloacae ENHKU01 Enterobacter cloacae subsp. cloacae NCTC 9394 Enterobacter cloacae EcWSU1 Enterobacter cloacae subsp. dissolvens SDM Enterobacter lignolyticus Enterobacter asburiae LF7a Enterobacter asburiae L1 Enterobacter aerogenes KCTC 2190 Enterobacter aerogenes EA1509E Enterobacter sp. R4-368 Cronobacter sakazakii ATCC BAA-894 Cronobacter sakazakii ES15 Cronobacter sakazakii Sp291 Cronobacter sakazakii CMCC 45402 Cronobacter turicensis Klebsiella pneumoniae subsp. pneumoniae MGH 78578 Klebsiella pneumoniae NTUH-K2044 Klebsiella pneumoniae subsp. pneumoniae HS11286 Klebsiella pneumoniae subsp. pneumoniae 1084 Klebsiella pneumoniae 342 Klebsiella pneumoniae KCTC 2242 Klebsiella pneumoniae Klebsiella pneumoniae JM45 Klebsiella pneumoniae CG43 Klebsiella pneumoniae 30660/NJST258\_1 Klebsiella pneumoniae 30684/NJST258\_2 Klebsiella variicola Klebsiella oxytoca KCTC 1686 Klebsiella oxytoca E718 Klebsiella oxytoca HKOPL1 Citrobacter koseri Citrobacter rodentium Citrobacter freundii Serratia proteamaculans Serratia plymuthica AS9 Serratia plymuthica 4Rx13 Serratia plymuthica S13 Serratia sp. AS12 Serratia sp. AS13 Serratia marcescens FGI94 Serratia marcescens WW4 Serratia liquefaciens Serratia sp. ATCC 39006 Serratia fonticola Proteus mirabilis HI4320 Proteus mirabilis BB2000 Edwardsiella ictaluri Edwardsiella tarda EIB202 Edwardsiella tarda FL6-60 Edwardsiella piscicida C07-087 Candidatus Blochmannia floridanus (Camponotus floridanus) Candidatus Blochmannia pennsylvanicus (Camponotus pennsylvanicus) Candidatus Blochmannia vafer (Camponotus vafer) Candidatus Blochmannia chromaiodes (Camponotus chromaiodes) Dickeya dadantii Ech703 Dickeya dadantii Ech586 Dickeya dadantii 3937 Dickeya zeae Xenorhabdus bovienii Xenorhabdus nematophila Pantoea ananatis LMG 20103 Pantoea ananatis LMG 5342 Pantoea ananatis AJ13355 Pantoea ananatis PA13 Pantoea vagans Pantoea sp. At-9b Rahnella sp. Y9602 Rahnella aquatilis CIP 78.65 = ATCC 33071 Rahnella aquatilis HX2 Providencia stuartii Shimwellia blattae Morganella morganii Raoultella ornithinolytica Enterobacteriaceae bacterium FGI 57 Plautia stali symbiont Haemophilus influenzae Rd KW20 (serotype d) Haemophilus influenzae 86-028NP (nontypeable) Haemophilus influenzae PittEE (nontypeable) Haemophilus influenzae PittGG (nontypeable) Haemophilus influenzae F3031 (nontypeable) Haemophilus influenzae F3047 (nontypeable) Haemophilus influenzae 10810 (serotype b) Haemophilus influenzae R2846 (nontypeable) Haemophilus influenzae R2866 (nontypeable) Haemophilus influenzae KR494 (serotype f) Haemophilus parasuis SH0165 Haemophilus parasuis ZJ0906 Haemophilus parainfluenzae Haemophilus somnus 129PT Haemophilus somnus 2336 Pasteurella multocida subsp. multocida Pm70 Pasteurella multocida subsp. multocida HN06 Pasteurella multocida subsp. multocida 3480 Pasteurella multocida 36950 Mannheimia succiniciproducens Mannheimia haemolytica USDA-ARS-SAM-185 Mannheimia haemolytica USDA-ARS-USMARC-183 Mannheimia haemolytica M42548 Mannheimia haemolytica D153 Mannheimia haemolytica D171 Mannheimia haemolytica D174 Mannheimia haemolytica USMARC\_2286 Mannheimia varigena USDA-ARS-USMARC-1261 Mannheimia varigena USDA-ARS-USMARC-1296 Mannheimia varigena USDA-ARS-USMARC-1312 Mannheimia varigena USDA-ARS-USMARC-1388 Actinobacillus pleuropneumoniae L20 (serotype 5b) Actinobacillus pleuropneumoniae JL03 (serotype 3) Actinobacillus pleuropneumoniae AP76 (serotype 7) Actinobacillus succinogenes Actinobacillus suis H91-0380 Aggregatibacter aphrophilus Aggregatibacter actinomycetemcomitans D11S-1 Aggregatibacter actinomycetemcomitans ANH9381 Aggregatibacter actinomycetemcomitans D7S-1 Aggregatibacter actinomycetemcomitans HK1651 Gallibacterium anatis Bibersteinia trehalosi USDA-ARS-USMARC-192 Bibersteinia trehalosi USDA-ARS-USMARC-188 Bibersteinia trehalosi USDA-ARS-USMARC-189 Bibersteinia trehalosi USDA-ARS-USMARC-190 Xylella fastidiosa 9a5c Xylella fastidiosa Temecula1 Xylella fastidiosa M12 Xylella fastidiosa M23 Xylella fastidiosa subsp. fastidiosa GB514 Xanthomonas campestris pv. campestris ATCC 33913 Xanthomonas campestris pv. campestris 8004 Xanthomonas campestris pv. campestris B100 Xanthomonas campestris pv. raphani Xanthomonas campestris pv. vesicatoria Xanthomonas citri pv. citri 306 Xanthomonas citri subsp. citri Aw12879 Xanthomonas alfalfae Xanthomonas axonopodis Xac29-1 Xanthomonas oryzae pv. oryzae KACC 10331 Xanthomonas oryzae pv. oryzae MAFF311018 Xanthomonas oryzae pv. oryzae PXO99A Xanthomonas oryzae pv. oryzicola Xanthomonas albilineans Xanthomonas fuscans Stenotrophomonas maltophilia K279a Stenotrophomonas maltophilia R551-3 Stenotrophomonas maltophilia JV3 Stenotrophomonas maltophilia D457 Pseudoxanthomonas suwonensis Pseudoxanthomonas spadix Frateuria aurantia Rhodanobacter denitrificans Dyella jiangningensis Dyella japonica Vibrio cholerae O1 biovar El Tor N16961 Vibrio cholerae O1 2010EL-1786 Vibrio cholerae MJ-1236 Vibrio cholerae O395 Vibrio cholerae O395 Vibrio cholerae M66-2 Vibrio cholerae IEC224 Vibrio cholerae LMA3984-4 Vibrio vulnificus CMCP6 Vibrio vulnificus YJ016 Vibrio vulnificus MO6-24/O Vibrio parahaemolyticus RIMD 2210633 Vibrio parahaemolyticus BB22OP Vibrio parahaemolyticus O1:K33 CDC\_K4557 Vibrio parahaemolyticus O1:Kuk FDA\_R31 Vibrio parahaemolyticus UCM-V493 Vibrio campbellii Vibrio campbellii Vibrio alginolyticus Vibrio splendidus Vibrio sp. Ex25 Vibrio sp. EJY3 Vibrio furnissii Vibrio nigripulchritudo Vibrio anguillarum 775 Vibrio anguillarum M3 Aliivibrio fischeri ES114 Aiivibrio fischeri MJ11 Aliivibrio salmonicida Photobacterium profundum Pseudomonas aeruginosa PAO1 Pseudomonas aeruginosa PAO1-VE13 Pseudomonas aeruginosa PAO1-VE2 Pseudomonas aeruginosa UCBPP-PA14 Pseudomonas aeruginosa PA7 Pseudomonas aeruginosa LESB58 Pseudomonas aeruginosa M18 Pseudomonas aeruginosa NCGM2.S1 Pseudomonas aeruginosa DK2 Pseudomonas aeruginosa B136-33 Pseudomonas aeruginosa RP73 Pseudomonas aeruginosa PA1 Pseudomonas aeruginosa PA1R Pseudomonas aeruginosa MTB-1 Pseudomonas aeruginosa LES431 Pseudomonas aeruginosa SCV20265 Pseudomonas aeruginosa PA38182 Pseudomonas aeruginosa YL84 Pseudomonas aeruginosa c7447m Pseudomonas aeruginosa PAO581 Pseudomonas putida KT2440 Pseudomonas putida F1 Pseudomonas putida GB-1 Pseudomonas putida W619 Pseudomonas putida S16 Pseudomonas putida BIRD-1 Pseudomonas putida ND6 Pseudomonas putida DOT-T1E Pseudomonas putida HB3267 Pseudomonas putida H8234 Pseudomonas putida NBRC 14164 Pseudomonas sp. UW4 Pseudomonas syringae pv. tomato DC3000 Pseudomonas syringae pv. syringae B728a Pseudomonas syringae CC1557 Pseudomonas syringae pv. phaseolicola 1448A Pseudomonas cichorii Pseudomonas protegens Pf-5 Pseudomonas protegens CHA0 Pseudomonas fluorescens Pf0-1 Pseudomonas fluorescens SBW25 Pseudomonas fluorescens F113 Pseudomonas fluorescens A506 Pseudomonas poae Pseudomonas entomophila Pseudomonas mendocina ymp Pseudomonas mendocina NK-01 Pseudomonas stutzeri A1501 Pseudomonas stutzeri ATCC 17588 Pseudomonas stutzeri DSM 4166 Pseudomonas stutzeri CCUG 29243 Pseudomonas stutzeri DSM 10701 Pseudomonas stutzeri RCH2 Pseudomonas brassicacearum subsp. brassicacearum NFM421 Pseudomonas brassicacearum DF41 Pseudomonas fulva Pseudomonas denitrificans Pseudomonas resinovorans Pseudomonas sp. VLB120 Pseudomonas sp. TKP Pseudomonas monteilii SB3078 Pseudomonas monteilii SB3101 Pseudomonas knackmussii Pseudomonas chlororaphis Cellvibrio japonicus Azotobacter vinelandii DJ Azotobacter vinelandii CA Azotobacter vinelandii CA6 Psychrobacter arcticus Psychrobacter cryohalolentis Psychrobacter sp. PRwf-1 Psychrobacter sp. G Acinetobacter sp. ADP1 Acinetobacter oleivorans Acinetobacter baumannii ATCC 17978 Acinetobacter baumannii SDF Acinetobacter baumannii AYE Acinetobacter baumannii ACICU Acinetobacter baumannii AB0057 Acinetobacter baumannii AB307-0294 Acinetobacter baumannii 1656-2 Acinetobacter baumannii MDR-ZJ06 Acinetobacter baumannii MDR-TJ Acinetobacter baumannii TCDC-AB0715 Acinetobacter baumannii TYTH-1 Acinetobacter baumannii D1279779 Acinetobacter baumannii BJAB07104 Acinetobacter baumannii BJAB0715 Acinetobacter baumannii BJAB0868 Acinetobacter baumannii ZW85-1 Acinetobacter calcoaceticus Moraxella catarrhalis RH4 Moraxella catarrhalis 25240 Shewanella oneidensis Shewanella denitrificans Shewanella frigidimarina Shewanella amazonensis Shewanella baltica OS155 Shewanella baltica OS185 Shewanella baltica OS195 Shewanella baltica OS223 Shewanella baltica OS678 Shewanella baltica OS117 Shewanella baltica BA175 Shewanella loihica Shewanella putrefaciens CN-32 Shewanella putrefaciens 200 Shewanella sediminis Shewanella pealeana Shewanella sp. MR-4 Shewanella sp. MR-7 Shewanella sp. ANA-3 Shewanella sp. W3-18-1 Shewanella halifaxensis Shewanella woodyi Shewanella piezotolerans Shewanella violacea Idiomarina loihiensis L2TR Idiomarina loihiensis GSL 199 Colwellia psychrerythraea Pseudoalteromonas haloplanktis Pseudoalteromonas atlantica Pseudoalteromonas sp. SM9913 Saccharophagus degradans Marinobacter hydrocarbonoclasticus VT8 Marinobacter hydrocarbonoclasticus ATCC 49840 Marinobacter adhaerens Marinobacter sp. BSs20148 Alteromonas macleodii Deep ecotype Alteromonas macleodii ATCC 27126 Alteromonas macleodii Balearic Sea AD45 Alteromonas macleodii English Channel 673 Alteromonas macleodii English Channel 615 Alteromonas macleodii Black Sea 11 Alteromonas macleodii AltDE1 Alteromonas macleodii Ionian Sea U4 Alteromonas macleodii Ionian Sea U7 Alteromonas macleodii Ionian Sea U8 Alteromonas macleodii Ionian Sea UM4b Alteromonas macleodii Ionian Sea UM7 Alteromonas macleodii Aegean Sea MED64 Alteromonas sp. SN2 Alteromonas australica Glaciecola sp. 4H-3-7+YE-5 Glaciecola nitratireducens Glaciecola psychrophila Psychromonas ingrahamii Psychromonas sp. CNPT3 Teredinibacter turnerae Ferrimonas balearica Coxiella burnetii RSA 493 Coxiella burnetii RSA 331 Coxiella burnetii Dugway 5J108-111 Coxiella burnetii CbuG\_Q212 Coxiella burnetii CbuK\_Q154 Legionella pneumophila subsp. pneumophila Philadelphia 1 Legionella pneumophila subsp. pneumophila HL06041035 Legionella pneumophila subsp. pneumophila Lorraine Legionella pneumophila subsp. pneumophila LPE509 Legionella pneumophila subsp. pneumophila Thunder Bay Legionella pneumophila Lens Legionella pneumophila Paris Legionella pneumophila Corby Legionella pneumophila 2300/99 Alcoy Legionella pneumophila subsp. pneumophila ATCC 43290 Legionella longbeachae Methylococcus capsulatus Methylomonas methanica Methylomicrobium alcaliphilum Francisella tularensis subsp. tularensis SCHU S4 Francisella tularensis subsp. tularensis FSC198 Francisella tularensis subsp. tularensis WY96-3418 Francisella tularensis subsp. tularensis NE061598 Francisella tularensis subsp. tularensis TI0902 Francisella tularensis TIGB03 Francisella tularensis subsp. holarctica LVS Francisella tularensis subsp. holarctica OSU18 Francisella tularensis subsp. holarctica FTNF002-00 Francisella tularensis subsp. holarctica F92 Francisella tularensis subsp. holarctica FSC200 Francisella tularensis subsp. holarctica PHIT-FT049 Francisella tularensis subsp. mediasiatica FSC147 Francisella tularensis subsp. novicida Francisella cf. novicida Fx1 Francisella cf. novicida 3523 Francisella philomiragia Francisella sp. TX077308 Francisella noatunensis subsp. orientalis Toba 04 Francisella noatunensis subsp. orientalis LADL--07-285A Thiomicrospira crunogena Thioalkalimicrobium cyclicum Methylophaga nitratireducenticrescens Methylophaga frappieri Cycloclasticus sp. P1 Cycloclasticus zancles Nitrosococcus oceani Nitrosococcus halophilus Nitrosococcus watsonii Allochromatium vinosum Thiocystis violascens Thioflavicoccus mobilis Alkalilimnicola ehrlichii Halorhodospira halophila Halorhodospira halochloris Thioalkalivibrio sulfidiphilus Thioalkalivibrio sp. K90mix Thioalkalivibrio nitratireducens Spiribacter salinus Spiribacter sp. UAH-SP71 Halothiobacillus neapolitanus Hahella chejuensis Chromohalobacter salexigens Halomonas elongata Halomonas campaniensis Alcanivorax borkumensis Alcanivorax dieselolei Kangiella koreensis Marinomonas sp. MWYL1 Marinomonas mediterranea Marinomonas posidonica Thalassolituus oleivorans MIL-1 Thalassolituus oleivorans R6-15 Aeromonas hydrophila subsp. hydrophila ATCC 7966 Aeromonas hydrophila ML09-119 Aeromonas hydrophila YL17 Aeromonas hydrophila AL09-71 Aeromonas hydrophila pc104A Aeromonas salmonicida Aeromonas veronii Aeromonas media Tolumonas auensis Oceanimonas sp. GK1 Dichelobacter nodosus Acidithiobacillus ferrooxidans ATCC 53993 Acidithiobacillus ferrooxidans ATCC 23270 Acidithiobacillus caldus Acidithiobacillus ferrivorans Gilliamella apicola Simiduia agarivorans Gamma proteobacterium HdN1 Neisseria meningitidis Z2491 (serogroup A) Neisseria meningitidis MC58 (serogroup B) Neisseria meningitidis alpha710 (serogroup B) Neisseria meningitidis H44/76 (serogroup B) Neisseria meningitidis FAM18 (serogroup C) Neisseria meningitidis 053442 (serogroup C) Neisseria meningitidis 8013 (serogroup C) Neisseria meningitidis alpha14 (cnl strain) Neisseria meningitidis G2136 (serogroup B) Neisseria meningitidis M01-240149 (serogroup B) Neisseria meningitidis M01-240355 (serogroup B) Neisseria meningitidis M04-240196 (serogroup B) Neisseria meningitidis WUE 2594 (serogroup A) Neisseria meningitidis NZ-05/33 (serogroup B) Neisseria gonorrhoeae FA 1090 Neisseria gonorrhoeae NCCP11945 Neisseria gonorrhoeae TCDC-NG08107 Neisseria lactamica Snodgrassella alvi Chromobacterium violaceum Laribacter hongkongensis Pseudogulbenkiania sp. NH8B Ralstonia solanacearum GMI1000 Ralstonia solanacearum CFBP2957 Ralstonia solanacearum PSI07 Ralstonia solanacearum Po82 Ralstonia solanacearum CMR15 Ralstonia solanacearum FQY\_4 Ralstonia pickettii 12J Ralstonia pickettii 12D Ralstonia pickettii DTP0602 Ralstonia eutropha JMP134 Ralstonia eutropha H16 Cupriavidus necator N-1 Cupriavidus metallidurans Cupriavidus taiwanensis Burkholderia mallei ATCC 23344 Burkholderia mallei SAVP1 Burkholderia mallei NCTC 10229 Burkholderia mallei NCTC 10247 Burkholderia pseudomallei K96243 Burkholderia pseudomallei 1710b Burkholderia pseudomallei 1106a Burkholderia pseudomallei 668 Burkholderia pseudomallei MSHR346 Burkholderia pseudomallei MSHR305 Burkholderia pseudomallei MSHR511 Burkholderia pseudomallei MSHR146 Burkholderia pseudomallei MSHR520 Burkholderia pseudomallei 1026b Burkholderia pseudomallei BPC006 Burkholderia pseudomallei NCTC 13179 Burkholderia thailandensis E264 Burkholderia thailandensis 2002721723 Burkholderia thailandensis E444 Burkholderia thailandensis H0587 Burkholderia thailandensis MSMB121 Burkholderia vietnamiensis Burkholderia lata Burkholderia cenocepacia AU1054 Burkholderia cenocepacia HI2424 Burkholderia cenocepacia MC0-3 Burkholderia cenocepacia J2315 Burkholderia ambifaria AMMD Burkholderia ambifaria MC40-6 Burkholderia multivorans ATCC 17616 (JGI) Burkholderia multivorans ATCC 17616 (Tohoku) Burkholderia cepacia Burkholderia xenovorans Burkholderia phymatum Burkholderia phytofirmans Burkholderia glumae Burkholderia sp. CCGE1001 Burkholderia sp. CCGE1002 Burkholderia sp. CCGE1003 Burkholderia rhizoxinica Burkholderia gladioli Burkholderia sp. YI23 Burkholderia sp. KJ006 Burkholderia phenoliruptrix Burkholderia sp. RPE64 Polynucleobacter necessarius subsp. asymbioticus Polynucleobacter necessarius subsp. necessarius Pandoraea pnomenusa 3kgm Pandoraea pnomenusa RB38 Pandoraea sp. RB-44 Bordetella pertussis Tohama I Bordetella pertussis CS Bordetella pertussis 18323 Bordetella parapertussis 12822 Bordetella parapertussis Bpp5 Bordetella bronchiseptica RB50 Bordetella bronchiseptica MO149 Bordetella bronchiseptica 253 Bordetella petrii Bordetella avium Bordetella holmesii Achromobacter xylosoxidans A8 Achromobacter xylosoxidans NH44784-1996 Achromobacter xylosoxidans NBRC 15126 = ATCC 27061 Taylorella equigenitalis MCE9 Taylorella equigenitalis ATCC 35865 Taylorella equigenitalis 14/56 Taylorella asinigenitalis MCE3 Taylorella asinigenitalis 14/45 Pusillimonas sp. T7-7 Advenella kashmirensis Advenella mimigardefordensis Castellaniella defragrans Basilea psittacipulmonis Rhodoferax ferrireducens Polaromonas sp. JS666 Polaromonas naphthalenivorans Acidovorax citrulli Acidovorax sp. JS42 Acidovorax ebreus Acidovorax avenae Acidovorax sp. KKS102 Verminephrobacter eiseniae Delftia acidovorans Delftia sp. Cs1-4 Variovorax paradoxus S110 Variovorax paradoxus EPS Variovorax paradoxus B4 Comamonas testosteroni CNB-2 Comamonas testosteroni TK102 Alicycliphilus denitrificans BC Alicycliphilus denitrificans K601 Ramlibacter tataouinensis Symbiobacter mobilis Methylibium petroleiphilum Herminiimonas arsenicoxydans Janthinobacterium sp. Marseille Janthinobacterium agaricidamnosum Herbaspirillum seropedicae Collimonas fungivorans Leptothrix cholodnii Thiomonas intermedia Thiomonas arsenitoxydans Rubrivivax gelatinosus Nitrosomonas europaea Nitrosomonas eutropha Nitrosomonas sp. AL212 Nitrosomonas sp. Is79A3 Nitrosospira multiformis Aromatoleum aromaticum Azoarcus sp. BH72 Azoarcus sp. KH32C Dechloromonas aromatica Thauera sp. MZ1T Dechlorosoma suillum Thiobacillus denitrificans Sulfuricella denitrificans Methylobacillus flagellatus Methylotenera mobilis Methylotenera versatilis Accumulibacter phosphatis Sideroxydans lithotrophicus Gallionella capsiferriformans Beta proteobacterium CB Helicobacter pylori 26695 Helicobacter pylori 26695 Helicobacter pylori J99 Helicobacter pylori HPAG1 Helicobacter pylori Shi470 Helicobacter pylori Shi112 Helicobacter pylori Shi169 Helicobacter pylori Shi417 Helicobacter pylori G27 Helicobacter pylori P12 Helicobacter pylori B38 Helicobacter pylori B8 Helicobacter pylori PeCan4 Helicobacter pylori PeCan18 Helicobacter pylori SJM180 Helicobacter pylori ELS37 Helicobacter pylori 35A Helicobacter pylori 908 Helicobacter pylori 2017 Helicobacter pylori 2018 Helicobacter pylori Cuz20 Helicobacter pylori F16 Helicobacter pylori F30 Helicobacter pylori F32 Helicobacter pylori F57 Helicobacter pylori Sat464 Helicobacter pylori 52 Helicobacter pylori v225d Helicobacter pylori 83 Helicobacter pylori SNT49 Helicobacter pylori Lithuania75 Helicobacter pylori Gambia94/24 Helicobacter pylori India7 Helicobacter pylori Puno120 Helicobacter pylori Puno135 Helicobacter pylori SouthAfrica7 Helicobacter pylori SouthAfrica20 Helicobacter pylori HUP-B14 Helicobacter pylori 51 Helicobacter pylori XZ274 Helicobacter pylori Rif1 Helicobacter pylori Rif2 Helicobacter pylori Aklavik117 Helicobacter pylori Aklavik86 Helicobacter pylori OK113 Helicobacter pylori OK310 Helicobacter pylori oki102 Helicobacter pylori UM032 Helicobacter pylori UM037 Helicobacter pylori UM066 Helicobacter pylori UM299 Helicobacter pylori UM298 Helicobacter pylori BM012A Helicobacter pylori BM012S Helicobacter hepaticus Helicobacter acinonychis Helicobacter mustelae Helicobacter felis Helicobacter bizzozeronii Helicobacter cinaedi PAGU611 Helicobacter cinaedi ATCC BAA-847 Helicobacter heilmannii Wolinella succinogenes Sulfurimonas denitrificans Sulfurimonas autotrophica Sulfuricurvum kujiense Uncultured Sulfuricurvum sp. RIFRC-1 Campylobacter jejuni subsp. jejuni NCTC 11168 = ATCC 700819 Campylobacter jejuni subsp. jejuni NCTC 11168-BN148 Campylobacter jejuni subsp. jejuni 81-176 Campylobacter jejuni subsp. jejuni 81116 Campylobacter jejuni subsp. jejuni ICDCCJ07001 Campylobacter jejuni subsp. jejuni IA3902 Campylobacter jejuni subsp. jejuni M1 Campylobacter jejuni subsp. jejuni S3 Campylobacter jejuni subsp. jejuni PT14 Campylobacter jejuni subsp. jejuni 00-2426 Campylobacter jejuni subsp. jejuni 00-2538 Campylobacter jejuni subsp. jejuni 00-2544 Campylobacter jejuni subsp. jejuni 00-2425 Campylobacter jejuni subsp. jejuni R14 Campylobacter jejuni RM1221 Campylobacter jejuni 32488 Campylobacter jejuni 4031 Campylobacter fetus subsp. fetus 82-40 Campylobacter fetus subsp. venerealis cfvi03/293 Campylobacter fetus subsp. testudinum 03-427 Campylobacter curvus Campylobacter hominis Campylobacter concisus Arcobacter butzleri RM4018 Arcobacter nitrofigilis Arcobacter sp. L Sulfurospirillum deleyianum Sulfurospirillum barnesii Sulfurospirillum multivorans Nitratifractor salsuginis Nitratiruptor sp. SB155-2 Sulfurovum sp. NBC37-1 Nautilia profundicola Geobacter sulfurreducens PCA Geobacter sulfurreducens KN400 Geobacter metallireducens Geobacter uraniireducens Geobacter lovleyi Geobacter bemidjiensis Geobacter sp. FRC-32 Geobacter sp. M21 Geobacter sp. M18 Pelobacter carbinolicus Pelobacter propionicus Desulfovibrio vulgaris Hildenborough Desulfovibrio vulgaris DP4 Desulfovibrio vulgaris Miyazaki F Desulfovibrio vulgaris RCH1 Desulfovibrio alaskensis Desulfovibrio desulfuricans ATCC 27774 Desulfovibrio desulfuricans ND132 Desulfovibrio magneticus Desulfovibrio salexigens Desulfovibrio aespoeensis Desulfovibrio hydrothermalis Desulfovibrio piezophilus Desulfovibrio gigas Lawsonia intracellularis PHE/MN1-00 Lawsonia intracellularis N343 Desulfomicrobium baculatum Desulfohalobium retbaense Bdellovibrio bacteriovorus HD100 Bdellovibrio bacteriovorus Tiberius Bdellovibrio bacteriovorus W Bdellovibrio bacteriovorus 109J Bdellovibrio exovorus Bacteriovorax marinus Desulfotalea psychrophila Desulfurivibrio alkaliphilus Desulfobulbus propionicus Desulfocapsa sulfexigens Candidatus Desulfococcus oleovorans Desulfatibacillum alkenivorans Desulfobacterium autotrophicum Desulfobacula toluolica Anaeromyxobacter dehalogenans 2CP-C Anaeromyxobacter dehalogenans 2CP-1 Anaeromyxobacter sp. Fw109-5 Anaeromyxobacter sp. K Myxococcus xanthus Myxococcus fulvus Myxococcus stipitatus Corallococcus coralloides Stigmatella aurantiaca Sorangium cellulosum So ce 56 Sorangium cellulosum So0157-2 Haliangium ochraceum Syntrophus aciditrophicus Desulfobacca acetoxidans Desulfomonile tiedjei Syntrophobacter fumaroxidans Desulfarculus baarsii Hippea maritima Desulfurella acetivorans Rickettsia prowazekii Madrid E Rickettsia prowazekii BuV67-CWPP Rickettsia prowazekii Chernikova Rickettsia prowazekii Dachau Rickettsia prowazekii GvV257 Rickettsia prowazekii Katsinyian Rickettsia prowazekii RpGvF24 Rickettsia prowazekii Rp22 Rickettsia prowazekii Breinl Rickettsia prowazekii NMRC Madrid E Rickettsia typhi Wilmington Rickettsia typhi TH1527 Rickettsia typhi B9991CWPP Rickettsia canadensis McKiel Rickettsia canadensis CA410 Rickettsia bellii RML369-C Rickettsia bellii OSU 85-389 Rickettsia conorii Rickettsia felis Rickettsia akari Rickettsia rickettsii Sheila Smith Rickettsia rickettsii Iowa Rickettsia rickettsii Arizona Rickettsia rickettsii Colombia Rickettsia rickettsii Hauke Rickettsia rickettsii Brazil Rickettsia rickettsii Hino Rickettsia rickettsii Hlp#2 Rickettsia massiliae MTU5 Rickettsia massiliae AZT80 Rickettsia peacockii Rickettsia africae Rickettsia heilongjiangensis Rickettsia japonica Rickettsia slovaca 13-B Rickettsia slovaca D-CWPP Rickettsia philipii Rickettsia australis Rickettsia montanensis Rickettsia parkeri Rickettsia rhipicephali Candidatus Rickettsia amblyommii Ehrlichia ruminantium Welgevonden (South Africa) Candidatus Midichloria mitochondrii Candidatus Paracaedibacter acanthamoebae Mesorhizobium loti Mesorhizobium ciceri Mesorhizobium opportunistum Mesorhizobium australicum Chelativorans sp. BNC1 Parvibaculum lavamentivorans Sinorhizobium meliloti 1021 Sinorhizobium meliloti AK83 Sinorhizobium meliloti BL225C Sinorhizobium meliloti SM11 Sinorhizobium meliloti Rm41 Sinorhizobium meliloti GR4 Sinorhizobium meliloti 2011 Sinorhizobium medicae Sinorhizobium fredii NGR234 Sinorhizobium fredii HH103 Sinorhizobium fredii USDA 257 Ensifer adhaerens Agrobacterium fabrum Agrobacterium radiobacter Agrobacterium vitis Agrobacterium sp. H13-3 Rhizobium etli CFN 42 Rhizobium etli CIAT 652 Rhizobium etli bv. mimosae Rhizobium leguminosarum bv. viciae 3841 Rhizobium leguminosarum bv. trifolii WSM2304 Rhizobium leguminosarum bv. trifolii WSM1325 Rhizobium leguminosarum bv. trifolii WSM1689 Rhizobium leguminosarum bv. trifolii CB782 Rhizobium tropici Rhizobium sp. IRBG74 Rhizobium sp. LPU83 Neorhizobium galegae bv. officinalis bv. officinalis HAMBI 1141 Candidatus Liberibacter asiaticus psy62 Candidatus Liberibacter asiaticus gxpsy Candidatus Liberibacter solanacearum Liberibacter crescens Candidatus Liberibacter americanus Brucella melitensis bv. 1 16M Brucella melitensis ATCC 23457 Brucella melitensis M28 Brucella melitensis M5-90 Brucella melitensis NI Brucella abortus 2308 Brucella abortus 9-941 Brucella abortus S19 Brucella abortus A13334 Brucella suis 1330 Brucella suis 1330 Brucella suis ATCC 23445 Brucella suis VBI22 Brucella suis bv. 1 Brucella ovis Brucella canis ATCC 23365 Brucella canis HSK A52141 Brucella canis Oliveri Brucella microti Brucella pinnipedialis B2/94 Brucella ceti TE10759-12 Brucella ceti TE28753-12 Ochrobactrum anthropi ATCC 49188 Bradyrhizobium diazoefficiens USDA 110 Bradyrhizobium japonicum USDA 6 Bradyrhizobium sp. ORS 278 Bradyrhizobium sp. BTAi1 Bradyrhizobium sp. S23321 Bradyrhizobium oligotrophicum Rhodopseudomonas palustris CGA009 Rhodopseudomonas palustris HaA2 Rhodopseudomonas palustris BisB18 Rhodopseudomonas palustris BisB5 Rhodopseudomonas palustris BisA53 Rhodopseudomonas palustris TIE-1 Rhodopseudomonas palustris DX-1 Nitrobacter winogradskyi Nitrobacter hamburgensis Oligotropha carboxidovorans OM5 (Mississippi) Oligotropha carboxidovorans OM5 (Goettingen) Oligotropha carboxidovorans OM4 Bartonella henselae Houston-1 Bartonella henselae BM1374163 Bartonella quintana RM-11 Bartonella tribocorum CIP 105476 Bartonella grahamii Bartonella australis Bartonella vinsonii Xanthobacter autotrophicus Azorhizobium caulinodans Starkeya novella Methylobacterium extorquens PA1 Methylobacterium extorquens AM1 Methylobacterium extorquens DM4 Methylobacterium extorquens CM4 Methylobacterium radiotolerans Methylobacterium sp. 4-46 Methylobacterium populi Methylobacterium nodulans Beijerinckia indica Methylocella silvestris Hyphomicrobium denitrificans ATCC 51888 Hyphomicrobium denitrificans 1NES1 Hyphomicrobium sp. MC1 Hyphomicrobium nitrativorans Rhodomicrobium vannielii Pelagibacterium halotolerans Methylocystis sp. SC2 Caulobacter crescentus CB15 Caulobacter crescentus NA1000 Caulobacter sp. K31 Caulobacter segnis Phenylobacterium zucineum Brevundimonas subvibrioides Asticcacaulis excentricus Ruegeria pomeroyi Ruegeria sp. TM1040 Rhodobacter sphaeroides 2.4.1 Rhodobacter sphaeroides ATCC 17029 Rhodobacter sphaeroides ATCC 17025 Rhodobacter sphaeroides KD131 Rhodobacter capsulatus Jannaschia sp. CCS1 Roseobacter denitrificans Roseobacter litoralis Paracoccus denitrificans Paracoccus aminophilus Dinoroseobacter shibae Ketogulonicigenium vulgare Y25 Ketogulonicigenium vulgare WSH-001 Pseudovibrio sp. FO-BEG1 Phaeobacter inhibens Phaeobacter gallaeciensis 2.10 Phaeobacter gallaeciensis DSM 26640 Octadecabacter antarcticus Octadecabacter arcticus Leisingera methylohalidivorans Roseibacterium elongatum Planktomarina temperata Maricaulis maris Hyphomonas neptunium Hirschia baltica Zymomonas mobilis subsp. mobilis ZM4 Zymomonas mobilis subsp. mobilis NCIMB 11163 Zymomonas mobilis subsp. mobilis ATCC 10988 Zymomonas mobilis subsp. mobilis ATCC 29191 Zymomonas mobilis subsp. mobilis CP4 = NRRL B-14023 Zymomonas mobilis subsp. mobilis NRRL B-12526 Zymomonas mobilis subsp. pomaceae ATCC 29192 Novosphingobium aromaticivorans Novosphingobium sp. PP1Y Sphingopyxis alaskensis Sphingomonas wittichii Sphingomonas sp. MM-1 Sphingobium japonicum Sphingobium chlorophenolicum Sphingobium sp. SYK-6 Erythrobacter litoralis Gluconobacter oxydans 621H Gluconobacter oxydans H24 Granulibacter bethesdensis CGDNIH1 Granulibacter bethesdensis CGDNIH2 Granulibacter bethesdensis CGDNIH3 Granulibacter bethesdensis CGDNIH4 Acidiphilium cryptum Acidiphilium multivorum Gluconacetobacter diazotrophicus PAl 5 (Brazil) Gluconacetobacter diazotrophicus PAl 5 (JGI) Gluconacetobacter medellinensis NBRC 3288 Gluconacetobacter xylinus E25 Acetobacter pasteurianus IFO 3283-01 Acetobacter pasteurianus IFO 3283-01-42C Acetobacter pasteurianus IFO 3283-03 Acetobacter pasteurianus IFO 3283-07 Acetobacter pasteurianus IFO 3283-12 Acetobacter pasteurianus IFO 3283-22 Acetobacter pasteurianus IFO 3283-26 Acetobacter pasteurianus IFO 3283-32 Acetobacter pasteurianus 386B Rhodospirillum rubrum ATCC 11170 Rhodospirillum rubrum F11 Rhodospirillum centenum Rhodospirillum photometricum Magnetospirillum magneticum Magnetospirillum gryphiswaldense Azospirillum sp. B510 Azospirillum lipoferum Azospirillum brasilense Tistrella mobilis Parvularcula bermudensis Magnetococcus marinus Micavibrio aeruginosavorus ARL-13 Micavibrio aeruginosavorus EPB Polymorphum gilvum Candidatus Pelagibacter ubique Candidatus Pelagibacter sp. IMCC9063 Alpha proteobacterium HIMB59 Alpha proteobacterium HIMB5 Candidatus Puniceispirillum marinum Bacillus subtilis subsp. subtilis 168 Bacillus subtilis subsp. subtilis RO-NN-1 Bacillus subtilis subsp. subtilis BSP1 Bacillus subtilis subsp. subtilis 6051-HGW Bacillus subtilis subsp. subtilis BAB-1 Bacillus subtilis subsp. spizizenii W23 Bacillus subtilis subsp. spizizenii TU-B-10 Bacillus subtilis subsp. natto BEST195 Bacillus subtilis BSn5 Bacillus subtilis QB928 Bacillus subtilis BEST7613 Bacillus subtilis XF-1 Bacillus subtilis PY79 Bacillus licheniformis ATCC 14580 Bacillus licheniformis DSM 13 = ATCC 14580 Bacillus licheniformis 9945A Bacillus amyloliquefaciens DSM 7 Bacillus amyloliquefaciens FZB42 Bacillus amyloliquefaciens subsp. plantarum CAU B946 Bacillus amyloliquefaciens subsp. plantarum YAU B9601-Y2 Bacillus amyloliquefaciens subsp. plantarum AS43.3 Bacillus amyloliquefaciens subsp. plantarum UCMB5036 Bacillus amyloliquefaciens subsp. plantarum UCMB5033 Bacillus amyloliquefaciens subsp. plantarum UCMB5113 Bacillus amyloliquefaciens subsp. plantarum NAU-B3 Bacillus amyloliquefaciens subsp. plantarum TrigoCor1448 Bacillus amyloliquefaciens TA208 Bacillus amyloliquefaciens LL3 Bacillus amyloliquefaciens XH7 Bacillus amyloliquefaciens Y2 Bacillus amyloliquefaciens IT-45 Bacillus amyloliquefaciens CC178 Bacillus amyloliquefaciens LFB112 Bacillus atrophaeus Bacillus halodurans Bacillus anthracis Ames Bacillus anthracis Ames 0581 Bacillus anthracis Sterne Bacillus anthracis CDC 684 Bacillus anthracis A0248 Bacillus anthracis H9401 Bacillus anthracis A16 Bacillus anthracis A16R Bacillus anthracis SVA11 Bacillus cereus biovar anthracis CI Bacillus cereus ATCC 14579 Bacillus cereus ATCC 10987 Bacillus cereus ZK Bacillus cereus AH187 Bacillus cereus B4264 Bacillus cereus AH820 Bacillus cereus G9842 Bacillus cereus Q1 Bacillus cereus 03BB102 Bacillus cereus NC7401 Bacillus cereus F837/76 Bacillus cereus FRI-35 Bacillus cytotoxicus Bacillus thuringiensis 97-27 Bacillus thuringiensis Al Hakam Bacillus thuringiensis BMB171 Bacillus thuringiensis serovar kurstaki HD73 Bacillus thuringiensis serovar chinensis CT-43 Bacillus thuringiensis serovar finitimus YBT-020 Bacillus thuringiensis MC28 Bacillus thuringiensis Bt407 Bacillus thuringiensis HD-771 Bacillus thuringiensis HD-789 Bacillus thuringiensis serovar thuringiensis IS5056 Bacillus thuringiensis YBT-1518 Bacillus weihenstephanensis Bacillus toyonensis Bacillus clausii Bacillus pumilus SAFR-032 Bacillus pumilus MTCC B6033 Bacillus pseudofirmus Bacillus megaterium QM B1551 Bacillus megaterium DSM 319 Bacillus megaterium WSH-002 Bacillus cellulosilyticus Bacillus coagulans 2-6 Bacillus coagulans 36D1 Bacillus sp. JS Bacillus sp. 1NLA3E Bacillus infantis Bacillus lehensis Bacillus methanolicus Oceanobacillus iheyensis Geobacillus kaustophilus Geobacillus thermodenitrificans Geobacillus thermoglucosidasius Geobacillus thermoleovorans Geobacillus sp. WCH70 Geobacillus sp. Y412MC61 Geobacillus sp. Y412MC52 Geobacillus sp. C56-T3 Geobacillus sp. Y4.1MC1 Geobacillus sp. GHH01 Geobacillus sp. JF8 Anoxybacillus flavithermus Amphibacillus xylanus Lysinibacillus sphaericus Lysinibacillus sp. GY32 Halobacillus halophilus Terribacillus aidingensis Virgibacillus sp. SK37 Bacillus selenitireducens Staphylococcus aureus subsp. aureus N315 (MRSA/VSSA) Staphylococcus aureus subsp. aureus Mu50 (MRSA/VISA) Staphylococcus aureus subsp. aureus Mu3 (MRSA/hetero-VISA) Staphylococcus aureus subsp. aureus JH1 (MRSA/VSSA) Staphylococcus aureus subsp. aureus JH9 (MRSA/VRSA) Staphylococcus aureus subsp. aureus MW2 (CA-MRSA) Staphylococcus aureus subsp. aureus MSSA476 (MSSA) Staphylococcus aureus subsp. aureus MRSA252 (MRSA) Staphylococcus aureus subsp. aureus COL (MRSA) Staphylococcus aureus subsp. aureus USA300\_TCH1516 (CA-MSSA) Staphylococcus aureus subsp. aureus USA300\_FPR3757 (CA-MRSA) Staphylococcus aureus subsp. aureus NCTC8325 Staphylococcus aureus subsp. aureus Newman Staphylococcus aureus subsp. aureus ED98 Staphylococcus aureus subsp. aureus M013 (CA-MRSA) Staphylococcus aureus subsp. aureus VC40 Staphylococcus aureus subsp. aureus MSHR1132 Staphylococcus aureus subsp. aureus ED133 Staphylococcus aureus subsp. aureus JKD6159 Staphylococcus aureus subsp. aureus JKD6008 (MRSA/VISA) Staphylococcus aureus subsp. aureus ECT-R 2 Staphylococcus aureus subsp. aureus T0131 (MRSA) Staphylococcus aureus subsp. aureus TCH60 Staphylococcus aureus subsp. aureus 11819-97 (CA-MRSA) Staphylococcus aureus subsp. aureus 71193 Staphylococcus aureus subsp. aureus HO 5096 0412 Staphylococcus aureus subsp. aureus TW20 (MRSA) Staphylococcus aureus subsp. aureus ST398 (MRSA) Staphylococcus aureus subsp. aureus LGA251 Staphylococcus aureus subsp. aureus 55/2053 Staphylococcus aureus subsp. aureus 6850 (MSSA) Staphylococcus aureus subsp. aureus CN1 (CA-MRSA) Staphylococcus aureus subsp. aureus SA40 (CA-MRSA) Staphylococcus aureus subsp. aureus SA957 (CA-MRSA) Staphylococcus aureus subsp. aureus Z172 (MRSA/VISA) Staphylococcus aureus RF122 Staphylococcus aureus 04-02981 Staphylococcus aureus 08BA02176 Staphylococcus aureus M1 Staphylococcus aureus CA-347 Staphylococcus aureus Bmb9393 Staphylococcus aureus USA300-ISMMS1 Staphylococcus aureus ST228/10388 Staphylococcus aureus ST228/10497 Staphylococcus aureus ST228/15532 Staphylococcus aureus ST228/16035 Staphylococcus aureus ST228/18412 Staphylococcus aureus ST228/16125 Staphylococcus aureus ST228/18341 Staphylococcus aureus ST228/18583 Staphylococcus epidermidis ATCC 12228 Staphylococcus epidermidis RP62A Staphylococcus haemolyticus Staphylococcus saprophyticus Staphylococcus carnosus Staphylococcus lugdunensis HKU09-01 Staphylococcus lugdunensis N920143 Staphylococcus pseudintermedius HKU10-03 Staphylococcus pseudintermedius ED99 Staphylococcus warneri Staphylococcus pasteuri Macrococcus caseolyticus Listeria monocytogenes EGD-e Listeria monocytogenes F2365 Listeria monocytogenes HCC23 Listeria monocytogenes Clip81459 Listeria monocytogenes 08-5578 Listeria monocytogenes 08-5923 Listeria monocytogenes 10403S Listeria monocytogenes FSL R2-561 Listeria monocytogenes Finland 1998 Listeria monocytogenes J0161 Listeria monocytogenes M7 Listeria monocytogenes L99 Listeria monocytogenes 07PF0776 Listeria monocytogenes SLCC2755 Listeria monocytogenes SLCC2372 Listeria monocytogenes serotype 7 SLCC2482 Listeria monocytogenes SLCC2376 Listeria monocytogenes SLCC5850 Listeria monocytogenes SLCC7179 Listeria monocytogenes SLCC2378 Listeria monocytogenes SLCC2479 Listeria monocytogenes SLCC2540 Listeria monocytogenes ATCC 19117 Listeria monocytogenes L312 Listeria monocytogenes serotype 4b LL195 Listeria monocytogenes La111 Listeria monocytogenes N53-1 Listeria monocytogenes J1-220 Listeria monocytogenes J1816 Listeria monocytogenes EGD Listeria monocytogenes WSLC1001 Listeria monocytogenes WSLC1042 Listeria monocytogenes 6179 Listeria monocytogenes R479a Listeria innocua Listeria welshimeri Listeria seeligeri Listeria ivanovii subsp. ivanovii PAM 55 Listeria ivanovii WSLC3009 Exiguobacterium sibiricum Exiguobacterium sp. AT1b Exiguobacterium antarcticum Exiguobacterium sp. MH3 Brevibacillus brevis Paenibacillus sp. JDR-2 Paenibacillus sp. Y412MC10 Paenibacillus polymyxa E681 Paenibacillus polymyxa SC2 Paenibacillus polymyxa M1 Paenibacillus polymyxa CR1 Paenibacillus polymyxa SQR-21 Paenibacillus mucilaginosus KNP414 Paenibacillus mucilaginosus 3016 Paenibacillus mucilaginosus K02 Paenibacillus terrae Paenibacillus larvae Paenibacillus sabinae Thermobacillus composti Alicyclobacillus acidocaldarius subsp. acidocaldarius DSM 446 Alicyclobacillus acidocaldarius subsp. acidocaldarius Tc-4-1 Kyrpidia tusciae Solibacillus silvestris Lactococcus lactis subsp. lactis Il1403 Lactococcus lactis subsp. lactis KF147 Lactococcus lactis subsp. lactis CV56 Lactococcus lactis subsp. lactis IO-1 Lactococcus lactis subsp. lactis KLDS 4.0325 Lactococcus lactis subsp. cremoris SK11 Lactococcus lactis subsp. cremoris MG1363 Lactococcus lactis subsp. cremoris A76 Lactococcus lactis subsp. cremoris NZ9000 Lactococcus lactis subsp. cremoris UC509.9 Lactococcus lactis subsp. cremoris KW2 Lactococcus garvieae ATCC 49156 Lactococcus garvieae Lg2 Streptococcus pyogenes SF370 (serotype M1) Streptococcus pyogenes MGAS5005 (serotype M1) Streptococcus pyogenes M1 476 (serotype M1) Streptococcus pyogenes MGAS8232 (serotype M18) Streptococcus pyogenes MGAS315 (serotype M3) Streptococcus pyogenes SSI-1 (serotype M3) Streptococcus pyogenes MGAS10270 (serotype M2) Streptococcus pyogenes MGAS10750 (serotype M4) Streptococcus pyogenes MGAS2096 (serotype M12) Streptococcus pyogenes MGAS9429 (serotype M12) Streptococcus pyogenes Manfredo (serotype M5) Streptococcus pyogenes MGAS10394 (serotype M6) Streptococcus pyogenes MGAS6180 (serotype M28) Streptococcus pyogenes MGAS15252 (serotype M59) Streptococcus pyogenes NZ131 (serotype M49) Streptococcus pyogenes Alab49 (serotype M53) Streptococcus pyogenes MGAS1882 Streptococcus pyogenes A20 Streptococcus pyogenes HSC5 Streptococcus pneumoniae TIGR4 (virulent serotype 4) Streptococcus pneumoniae D39 (virulent serotype 2) Streptococcus pneumoniae R6 (avirulent) Streptococcus pneumoniae CGSP14 (serotype 14) Streptococcus pneumoniae G54 (serotype 19F) Streptococcus pneumoniae ATCC 700669 (serotype 23F ST81 lineage) Streptococcus pneumoniae Hungary19A 6 Streptococcus pneumoniae 70585 Streptococcus pneumoniae JJA Streptococcus pneumoniae P1031 Streptococcus pneumoniae Taiwan19F-14 Streptococcus pneumoniae TCH8431/19A Streptococcus pneumoniae 670-6B Streptococcus pneumoniae AP200 Streptococcus pneumoniae INV104 Streptococcus pneumoniae INV200 Streptococcus pneumoniae OXC141 Streptococcus pneumoniae ST556 Streptococcus pneumoniae SPNA45 Streptococcus pneumoniae gamPNI0373 Streptococcus pneumoniae SPN034156 Streptococcus pneumoniae SPN034183 Streptococcus pneumoniae SPN994038 Streptococcus pneumoniae SPN994039 Streptococcus pneumoniae A026 Streptococcus agalactiae 2603 (serotype V) Streptococcus agalactiae NEM316 (serotype III) Streptococcus agalactiae A909 (serotype Ia) Streptococcus agalactiae GD201008-001 (serotype Ia) Streptococcus agalactiae SA20-06 Streptococcus agalactiae 2-22 (serotype Ib) Streptococcus agalactiae 09mas018883 Streptococcus agalactiae ILRI005 Streptococcus agalactiae ILRI112 Streptococcus agalactiae 138P Streptococcus mutans UA159 Streptococcus mutans NN2025 Streptococcus mutans LJ23 Streptococcus mutans GS-5 Streptococcus thermophilus CNRZ1066 Streptococcus thermophilus LMG18311 Streptococcus thermophilus LMD-9 Streptococcus thermophilus ND03 Streptococcus thermophilus JIM 8232 Streptococcus thermophilus MN-ZLW-002 Streptococcus sanguinis Streptococcus suis 05ZYH33 Streptococcus suis 98HAH33 Streptococcus suis BM407 Streptococcus suis P1/7 Streptococcus suis SC84 Streptococcus suis ST3 Streptococcus suis A7 Streptococcus suis D12 Streptococcus suis D9 Streptococcus suis GZ1 Streptococcus suis JS14 Streptococcus suis SS12 Streptococcus suis ST1 Streptococcus suis S735 Streptococcus suis SC070731 Streptococcus suis TL13 Streptococcus suis T15 Streptococcus suis YB51 Streptococcus gordonii Streptococcus equi subsp. zooepidemicus H70 Streptococcus equi subsp. zooepidemicus MGCS10565 Streptococcus equi subsp. zooepidemicus ATCC 35246 Streptococcus equi subsp. equi 4047 Streptococcus uberis Streptococcus dysgalactiae subsp. equisimilis GGS\_124 Streptococcus dysgalactiae subsp. equisimilis ATCC 12394 Streptococcus dysgalactiae subsp. equisimilis RE378 Streptococcus dysgalactiae subsp. equisimilis AC-2713 Streptococcus dysgalactiae subsp. equisimilis 167 Streptococcus gallolyticus UCN34 Streptococcus gallolyticus subsp. gallolyticus ATCC BAA-2069 Streptococcus gallolyticus subsp. gallolyticus ATCC 43143 Streptococcus mitis Streptococcus oralis Streptococcus parauberis Streptococcus pasteurianus Streptococcus parasanguinis ATCC 15912 Streptococcus parasanguinis FW213 Streptococcus salivarius CCHSS3 Streptococcus salivarius 57.I Streptococcus salivarius JIM8777 Streptococcus pseudopneumoniae Streptococcus macedonicus Streptococcus infantarius Streptococcus intermedius JTH08 Streptococcus intermedius B196 Streptococcus intermedius C270 Streptococcus anginosus C1051 Streptococcus anginosus C238 Streptococcus constellatus subsp. pharyngis C1050 Streptococcus constellatus subsp. pharyngis C232 Streptococcus constellatus subsp. pharyngis C818 Streptococcus oligofermentans Streptococcus iniae Streptococcus lutetiensis Streptococcus sp. I-G2 Streptococcus sp. I-P16 Lactobacillus plantarum WCFS1 Lactobacillus plantarum JDM1 Lactobacillus plantarum ZJ316 Lactobacillus plantarum subsp. plantarum ST-III Lactobacillus plantarum subsp. plantarum P-8 Lactobacillus plantarum 16 Lactobacillus johnsonii NCC 533 Lactobacillus johnsonii FI9785 Lactobacillus johnsonii DPC 6026 Lactobacillus johnsonii N6.2 Lactobacillus acidophilus NCFM Lactobacillus acidophilus 30SC Lactobacillus acidophilus La-14 Lactobacillus sakei Lactobacillus salivarius UCC118 Lactobacillus salivarius CECT 5713 Lactobacillus delbrueckii subsp. bulgaricus ATCC 11842 Lactobacillus delbrueckii subsp. bulgaricus ATCC BAA-365 Lactobacillus delbrueckii subsp. bulgaricus ND02 Lactobacillus delbrueckii subsp. bulgaricus 2038 Lactobacillus brevis ATCC 367 Lactobacillus brevis KB290 Lactobacillus casei ATCC 334 Lactobacillus casei BL23 Lactobacillus casei Zhang Lactobacillus casei BD-II Lactobacillus casei LC2W Lactobacillus casei W56 Lactobacillus casei LOCK919 Lactobacillus gasseri Lactobacillus reuteri DSM 20016 Lactobacillus reuteri JCM 1112 Lactobacillus reuteri SD2112 Lactobacillus reuteri I5007 Lactobacillus reuteri TD1 Lactobacillus helveticus DPC 4571 Lactobacillus helveticus H10 Lactobacillus helveticus R0052 Lactobacillus helveticus CNRZ32 Lactobacillus helveticus H9 Lactobacillus fermentum IFO 3956 Lactobacillus fermentum CECT 5716 Lactobacillus fermentum F-6 Lactobacillus rhamnosus GG Lactobacillus rhamnosus GG Lactobacillus rhamnosus Lc 705 Lactobacillus rhamnosus ATCC 8530 Lactobacillus rhamnosus LOCK900 Lactobacillus rhamnosus LOCK908 Lactobacillus crispatus Lactobacillus amylovorus GRL 1112 Lactobacillus amylovorus GRL1118 Lactobacillus buchneri NRRL B-30929 Lactobacillus buchneri CD034 Lactobacillus kefiranofaciens Lactobacillus ruminis Lactobacillus sanfranciscensis Lactobacillus paracasei subsp. paracasei 8700:2 Lactobacillus paracasei N1115 Pediococcus pentosaceus ATCC 25745 Pediococcus pentosaceus SL4 Pediococcus claussenii Enterococcus faecalis V583 Enterococcus faecalis 62 Enterococcus faecalis OG1RF Enterococcus faecalis D32 Enterococcus faecalis Symbioflor 1 Enterococcus faecalis DENG1 Enterococcus faecium Aus0004 Enterococcus faecium Aus0085 Enterococcus faecium DO Enterococcus faecium NRRL B-2354 Enterococcus hirae Enterococcus sp. 7L76 Enterococcus casseliflavus Enterococcus mundtii Melissococcus plutonius ATCC 35311 Melissococcus plutonius DAT561 Tetragenococcus halophilus Oenococcus oeni Leuconostoc mesenteroides subsp. mesenteroides ATCC 8293 Leuconostoc mesenteroides subsp. mesenteroides J18 Leuconostoc mesenteroides KFRI-MG Leuconostoc citreum Leuconostoc kimchii Leuconostoc gasicomitatum Leuconostoc sp. C2 Leuconostoc carnosum Leuconostoc gelidum Weissella koreensis Weissella ceti Aerococcus urinae Carnobacterium sp. 17-4 Carnobacterium maltaromaticum Carnobacterium sp. WN1359 Clostridium acetobutylicum ATCC 824 Clostridium acetobutylicum DSM 1731 Clostridium acetobutylicum EA 2018 Clostridium perfringens 13 Clostridium perfringens ATCC 13124 Clostridium perfringens SM101 Clostridium tetani E88 Clostridium tetani 12124569 Clostridium novyi Clostridium botulinum A ATCC 3502 Clostridium botulinum A ATCC 19397 Clostridium botulinum A Hall Clostridium botulinum A2 Clostridium botulinum A3 Loch Maree Clostridium botulinum B Eklund 17B Clostridium botulinum B1 Okra Clostridium botulinum Ba4 Clostridium botulinum BKT015925 Clostridium botulinum E3 Clostridium botulinum F Langeland Clostridium botulinum F 230613 Clostridium botulinum H04402 065 Clostridium beijerinckii Clostridium kluyveri DSM 555 Clostridium kluyveri NBRC 12016 Clostridium cellulolyticum Clostridium ljungdahlii Clostridium cellulovorans Clostridium sp. SY8519 Clostridium sp. BNL1100 Clostridium saccharoperbutylacetonicum Clostridium pasteurianum Clostridium saccharobutylicum Clostridium autoethanogenum Clostridium sp. M2/40 Alkaliphilus metalliredigens Alkaliphilus oremlandii Candidatus Arthromitus sp. SFB-mouse-Japan Ruminiclostridium thermocellum ATCC 27405 Ruminiclostridium thermocellum DSM 1313 Clostridium clariflavum Eubacterium siraeum V10Sc8a Eubacterium siraeum 70/3 Clostridium stercorarium subsp. stercorarium DSM 8532 Clostridium stercorarium subsp. stercorarium DSM 8532 Ethanoligenens harbinense Ruminococcus albus Ruminococcus bromii Ruminococcus champanellensis Ruminococcus sp. SR1/5 Ruminococcus sp. 80/3 Faecalibacterium prausnitzii L2-6 Faecalibacterium prausnitzii SL3/3 Butyrivibrio proteoclasticus Butyrivibrio fibrisolvens Cellulosilyticum lentocellum Roseburia hominis Roseburia intestinalis XB6B4 Roseburia intestinalis M50/1 Coprococcus sp. ART55/1 Coprococcus catus Ruminococcus obeum Ruminococcus torques Lachnoclostridium phytofermentans Clostridium saccharolyticum WM1 Clostridium cf. saccharolyticum K10 Peptoclostridium difficile 630 Peptoclostridium difficile CD196 Peptoclostridium difficile R20291 Peptoclostridium difficile BI1 Clostridium sticklandii Filifactor alocis Symbiobacterium thermophilum Syntrophomonas wolfei Syntrophothermus lipocalidus Desulfitobacterium hafniense Y51 Desulfitobacterium hafniense DCB-2 Desulfitobacterium dehalogenans Desulfitobacterium dichloroeliminans Desulfotomaculum reducens Desulfotomaculum acetoxidans Desulfotomaculum carboxydivorans Desulfotomaculum kuznetsovii Desulfotomaculum ruminis Desulfotomaculum gibsoniae Pelotomaculum thermopropionicum Candidatus Desulforudis audaxviator Thermincola potens Syntrophobotulus glycolicus Desulfosporosinus orientis Desulfosporosinus acidiphilus Desulfosporosinus meridiei Dehalobacter sp. DCA Dehalobacter sp. CF Dehalobacter restrictus Heliobacterium modesticaldum Finegoldia magna Anaerococcus prevotii Eubacterium eligens Eubacterium rectale ATCC 33656 Eubacterium rectale DSM 17629 Eubacterium rectale M104/1 Eubacterium limosum Eubacterium acidaminophilum Acetobacterium woodii Oscillibacter valericigenes Thermaerobacter marianensis Sulfobacillus acidophilus TPY Sulfobacillus acidophilus DSM 10332 Clostridiales genomosp. BVAB3 Butyrate-producing bacterium SM4/1 Butyrate-producing bacterium SS3/4 Butyrate-producing bacterium SSC/2 Thermoanaerobacter tengcongensis Thermoanaerobacter sp. X514 Thermoanaerobacter sp. X513 Thermoanaerobacter pseudethanolicus Thermoanaerobacter italicus Thermoanaerobacter mathranii Thermoanaerobacter brockii Thermoanaerobacter wiegelii Carboxydothermus hydrogenoformans Tepidanaerobacter acetatoxydans Re1 Tepidanaerobacter acetatoxydans Re1 Moorella thermoacetica Thermacetogenium phaeum Caldicellulosiruptor saccharolyticus Caldicellulosiruptor bescii Caldicellulosiruptor obsidiansis Caldicellulosiruptor hydrothermalis Caldicellulosiruptor owensensis Caldicellulosiruptor kristjanssonii Caldicellulosiruptor kronotskyensis Caldicellulosiruptor lactoaceticus Thermosediminibacter oceani Thermoanaerobacterium thermosaccharolyticum DSM 571 Thermoanaerobacterium thermosaccharolyticum M0795 Thermoanaerobacterium xylanolyticum Thermoanaerobacterium saccharolyticum Coprothermobacter proteolyticus Thermodesulfobium narugense Mahella australiensis Natranaerobius thermophilus Halothermothrix orenii Halanaerobium hydrogeniformans Halanaerobium praevalens Acetohalobium arabaticum Halobacteroides halobius Veillonella parvula Selenomonas sputigena Selenomonas ruminantium Megasphaera elsdenii Megamonas hypermegale Pelosinus sp. UFO1 Acidaminococcus fermentans Acidaminococcus intestini Erysipelothrix rhusiopathiae Fujisawa Erysipelothrix rhusiopathiae SY1027 Eubacterium cylindroides Mycoplasma penetrans Acholeplasma laidlawii Acholeplasma brassicae Acholeplasma palmae Spiroplasma chrysopicola Spiroplasma apis Spiroplasma mirum Mycobacterium tuberculosis H37Rv Mycobacterium tuberculosis H37Rv Mycobacterium tuberculosis CDC1551 Mycobacterium tuberculosis H37Ra Mycobacterium tuberculosis F11 Mycobacterium tuberculosis KZN 1435 Mycobacterium tuberculosis KZN 4207 Mycobacterium tuberculosis KZN 605 Mycobacterium tuberculosis RGTB327 Mycobacterium tuberculosis RGTB423 Mycobacterium tuberculosis CCDC5079 Mycobacterium tuberculosis CCDC5079 Mycobacterium tuberculosis CCDC5180 Mycobacterium tuberculosis CTRI-2 Mycobacterium tuberculosis UT205 Mycobacterium tuberculosis Erdman = ATCC 35801 Mycobacterium tuberculosis Beijing/NITR203 Mycobacterium tuberculosis 7199-99 Mycobacterium tuberculosis CAS/NITR204 Mycobacterium tuberculosis EAI5/NITR206 Mycobacterium tuberculosis EAI5 Mycobacterium tuberculosis Haarlem/NITR202 Mycobacterium tuberculosis Haarlem Mycobacterium tuberculosis BT1 Mycobacterium tuberculosis BT2 Mycobacterium tuberculosis HKBS1 Mycobacterium bovis AF2122/97 Mycobacterium bovis BCG Pasteur 1173P2 Mycobacterium bovis BCG Tokyo 172 Mycobacterium bovis BCG Mexico Mycobacterium bovis BCG Korea 1168P Mycobacterium africanum Mycobacterium canettii CIPT 140010059 Mycobacterium canettii CIPT 140060008 Mycobacterium canettii CIPT 140070008 Mycobacterium canettii CIPT 140070010 Mycobacterium canettii CIPT 140070017 Mycobacterium leprae TN Mycobacterium leprae Br4923 Mycobacterium avium subsp. paratuberculosis K-10 Mycobacterium avium subsp. paratuberculosis MAP4 Mycobacterium avium 104 Mycobacterium intracellulare MOTT-02 Mycobacterium intracellulare MOTT-64 Mycobacterium intracellulare ATCC 13950 Mycobacterium indicus pranii Mycobacterium yongonense Mycobacterium smegmatis MC2 155 Mycobacterium smegmatis MC2 155 Mycobacterium smegmatis JS623 Mycobacterium ulcerans Mycobacterium vanbaalenii Mycobacterium gilvum PYR-GCK Mycobacterium gilvum Spyr1 Mycobacterium abscessus ATCC 19977 Mycobacterium abscessus subsp. bolletii 50594 Mycobacterium abscessus subsp. bolletii GO 06 Mycobacterium sp. MCS Mycobacterium sp. KMS Mycobacterium sp. JLS Mycobacterium sp. JDM601 Mycobacterium marinum Mycobacterium rhodesiae Mycobacterium sp. MOTT36Y Mycobacterium chubuense Mycobacterium liflandii Mycobacterium kansasii Mycobacterium neoaurum Amycolicicoccus subflavus Corynebacterium glutamicum ATCC 13032 (Kyowa Hakko) Corynebacterium glutamicum ATCC 13032 (Bielefeld) Corynebacterium glutamicum K051 Corynebacterium glutamicum R Corynebacterium glutamicum SCgG1 Corynebacterium glutamicum SCgG2 Corynebacterium glutamicum MB001 Corynebacterium efficiens Corynebacterium diphtheriae NCTC 13129 Corynebacterium diphtheriae 241 Corynebacterium diphtheriae INCA 402 Corynebacterium diphtheriae HC01 Corynebacterium diphtheriae HC02 Corynebacterium diphtheriae HC03 Corynebacterium diphtheriae HC04 Corynebacterium diphtheriae 31A Corynebacterium diphtheriae BH8 Corynebacterium diphtheriae C7 Corynebacterium diphtheriae CDCE 8392 Corynebacterium diphtheriae PW8 Corynebacterium diphtheriae VA01 Corynebacterium jeikeium Corynebacterium urealyticum DSM 7109 Corynebacterium urealyticum DSM 7111 Corynebacterium aurimucosum Corynebacterium kroppenstedtii Corynebacterium pseudotuberculosis FRC41 Corynebacterium pseudotuberculosis 3/99-5 Corynebacterium pseudotuberculosis 316 Corynebacterium pseudotuberculosis P54B96 Corynebacterium pseudotuberculosis 1002 Corynebacterium pseudotuberculosis C231 Corynebacterium pseudotuberculosis I19 Corynebacterium pseudotuberculosis PAT10 Corynebacterium pseudotuberculosis 267 Corynebacterium pseudotuberculosis 31 Corynebacterium pseudotuberculosis 1/06-A Corynebacterium pseudotuberculosis 42/02-A Corynebacterium pseudotuberculosis CIP 52.97 Corynebacterium pseudotuberculosis 258 Corynebacterium pseudotuberculosis Cp162 Corynebacterium resistens Corynebacterium ulcerans BR-AD22 Corynebacterium ulcerans 809 Corynebacterium ulcerans 0102 Corynebacterium variabile Corynebacterium halotolerans Corynebacterium callunae Corynebacterium terpenotabidum Corynebacterium maris Corynebacterium argentoratense Corynebacterium falsenii Corynebacterium casei Corynebacterium vitaeruminis Corynebacterium glycinophilum Corynebacterium atypicum Nocardia farcinica Nocardia cyriacigeorgica Nocardia brasiliensis Nocardia nova Rhodococcus jostii Rhodococcus erythropolis PR4 Rhodococcus erythropolis CCM2595 Rhodococcus opacus B4 Rhodococcus opacus PD630 Rhodococcus equi Rhodococcus pyridinivorans Gordonia bronchialis Gordonia polyisoprenivorans Gordonia sp. KTR9 Tsukamurella paurometabola Segniliparus rotundus Streptomyces coelicolor Streptomyces avermitilis Streptomyces griseus Streptomyces scabiei Streptomyces sp. SirexAA-E Streptomyces violaceusniger Streptomyces cattleya NRRL 8057 Streptomyces cattleya NRRL 8057 = DSM 46488 Streptomyces pratensis Streptomyces bingchenggensis Streptomyces hygroscopicus subsp. jinggangensis 5008 Streptomyces hygroscopicus subsp. jinggangensis TL01 Streptomyces venezuelae Streptomyces davawensis Streptomyces albus Streptomyces sp. PAMC26508 Streptomyces fulvissimus Streptomyces collinus Streptomyces rapamycinicus Streptomyces albulus Streptomyces lividans Kitasatospora setae Tropheryma whipplei Twist Tropheryma whipplei TW08/27 Leifsonia xyli subsp. xyli CTCB07 Leifsonia xyli subsp. cynodontis DSM 46306 Clavibacter michiganensis subsp. michiganensis Clavibacter michiganensis subsp. sepedonicus Clavibacter michiganensis subsp. nebraskensis Microbacterium testaceum Candidatus Rhodoluna lacicola Arthrobacter sp. FB24 Arthrobacter aurescens Arthrobacter chlorophenolicus Arthrobacter arilaitensis Arthrobacter phenanthrenivorans Arthrobacter sp. Rue61a Renibacterium salmoninarum Kocuria rhizophila Micrococcus luteus Rothia mucilaginosa Rothia dentocariosa Beutenbergia cavernae Brachybacterium faecium Jonesia denitrificans Kytococcus sedentarius Dermacoccus nishinomiyaensis Xylanimonas cellulosilytica Isoptericola variabilis Sanguibacter keddieii Cellulomonas flavigena Cellulomonas fimi Cellulomonas gilvus Intrasporangium calvum Propionibacterium acnes KPA171202 Propionibacterium acnes SK137 Propionibacterium acnes TypeIA2 P.acn17 Propionibacterium acnes TypeIA2 P.acn31 Propionibacterium acnes TypeIA2 P.acn33 Propionibacterium acnes 266 Propionibacterium acnes ATCC 11828 Propionibacterium acnes 6609 Propionibacterium acnes C1 Propionibacterium acnes HL096PA1 Propionibacterium freudenreichii Propionibacterium propionicum Propionibacterium acidipropionici Propionibacterium avidum Microlunatus phosphovorus Nocardioides sp. JS614 Kribbella flavida Thermobifida fusca Nocardiopsis dassonvillei Nocardiopsis alba Thermomonospora curvata Streptosporangium roseum Frankia sp. CcI3 Frankia sp. EAN1pec Frankia sp. EuI1c Frankia alni Frankia symbiont Acidothermus cellulolyticus Nakamurella multipartita Geodermatophilus obscurus Blastococcus saxobsidens Modestobacter marinus Kineococcus radiotolerans Saccharopolyspora erythraea Saccharomonospora viridis Thermobispora bispora Amycolatopsis mediterranei U32 Amycolatopsis mediterranei S699 Amycolatopsis mediterranei S699 Amycolatopsis mediterranei RB Amycolatopsis orientalis Amycolatopsis japonica Amycolatopsis methanolica Pseudonocardia dioxanivorans Actinosynnema mirum Saccharothrix espanaensis Kutzneria albida Salinispora tropica Salinispora arenicola Micromonospora aurantiaca Micromonospora sp. L5 Verrucosispora maris Actinoplanes missouriensis Actinoplanes sp. SE50/110 Actinoplanes sp. N902-109 Actinoplanes friuliensis Catenulispora acidiphila Stackebrandtia nassauensis Arcanobacterium haemolyticum Mobiluncus curtisii Trueperella pyogenes Actinobaculum schaalii Bifidobacterium longum NCC2705 Bifidobacterium longum DJO10A Bifidobacterium longum subsp. infantis ATCC 15697 (JGI) Bifidobacterium longum subsp. infantis ATCC 15697 (Tokyo) Bifidobacterium longum subsp. infantis 157F Bifidobacterium longum subsp. longum JDM301 Bifidobacterium longum subsp. longum BBMN68 Bifidobacterium longum subsp. longum JCM 1217 Bifidobacterium longum subsp. longum KACC 91563 Bifidobacterium longum subsp. longum F8 Bifidobacterium adolescentis ATCC 15703 Bifidobacterium animalis subsp. lactis AD011 Bifidobacterium animalis subsp. lactis Bl-04 Bifidobacterium animalis subsp. lactis DSM 10140 Bifidobacterium animalis subsp. lactis BB-12 Bifidobacterium animalis subsp. lactis BLC1 Bifidobacterium animalis subsp. lactis CNCM I-2494 Bifidobacterium animalis subsp. lactis V9 Bifidobacterium animalis subsp. lactis B420 Bifidobacterium animalis subsp. lactis Bi-07 Bifidobacterium animalis subsp. lactis Bl12 Bifidobacterium animalis subsp. lactis ATCC 27673 Bifidobacterium animalis subsp. animalis ATCC 25527 Bifidobacterium dentium Bifidobacterium bifidum S17 Bifidobacterium bifidum PRL2010 Bifidobacterium bifidum BGN4 Bifidobacterium breve ACS-071-V-Sch8b Bifidobacterium breve UCC2003 Bifidobacterium breve 12L Bifidobacterium breve 689b Bifidobacterium breve JCM 7017 Bifidobacterium breve JCM 7019 Bifidobacterium breve NCFB 2258 Bifidobacterium breve S27 Bifidobacterium asteroides Bifidobacterium thermophilum Gardnerella vaginalis 409-05 Gardnerella vaginalis ATCC 14019 Gardnerella vaginalis HMP9231 Rubrobacter xylanophilus Rubrobacter radiotolerans Conexibacter woesei Acidimicrobium ferrooxidans Ilumatobacter coccineus Cryptobacterium curtum Slackia heliotrinireducens Atopobium parvulum Eggerthella lenta Eggerthella sp. YY7918 Olsenella uli Coriobacterium glomerans Gordonibacter pamelaeae Adlercreutzia equolifaciens Parachlamydia acanthamoebae Waddlia chondrophila Simkania negevensis Opitutus terrae Coraliomargarita akajimensis Methylacidiphilum infernorum Akkermansia muciniphila Borrelia burgdorferi ZS7 Borrelia burgdorferi N40 Borrelia burgdorferi JD1 Borrelia burgdorferi CA382 Borrelia garinii PBi Borrelia garinii BgVir Borrelia garinii NMJW1 Borrelia afzelii (FLI) Borrelia afzelii (Maryland) Borrelia afzelii HLJ01 Borrelia bissettii Borrelia valaisiana Borrelia turicatae Borrelia hermsii Borrelia duttonii Borrelia recurrentis Borrelia crocidurae Borrelia miyamotoi Treponema denticola Treponema succinifaciens Treponema brennaborense Treponema azotonutricium Treponema primitia Treponema pedis Treponema caldaria Spirochaeta smaragdinae Spirochaeta thermophila DSM 6192 Spirochaeta thermophila DSM 6578 Spirochaeta africana Spirochaeta sp. L21-RPul-D2 Sphaerochaeta globosa Sphaerochaeta coccoides Sphaerochaeta pleomorpha Leptospira interrogans serovar Lai 56601 Leptospira interrogans serovar Lai IPAV Leptospira interrogans serovar Copenhageni Leptospira borgpetersenii JB197 Leptospira borgpetersenii L550 Leptospira biflexa serovar Patoc Patoc 1 (Paris) Leptospira biflexa serovar Patoc Patoc 1 (Ames) Turneriella parva Brachyspira hyodysenteriae Brachyspira murdochii Brachyspira pilosicoli 95/1000 Brachyspira pilosicoli B2904 Brachyspira pilosicoli P43/6/78 Brachyspira pilosicoli WesB Brachyspira intermedia Candidatus Koribacter versatilis Acidobacterium capsulatum Granulicella tundricola Granulicella mallensis Terriglobus saanensis Terriglobus roseus Candidatus Solibacter usitatus Candidatus Chloracidobacterium thermophilum B Fibrobacter succinogenes Fibrobacter succinogenes Elusimicrobium minutum Uncultured Termite group 1 bacterium phylotype Rs-D17 Fusobacterium nucleatum subsp. nucleatum ATCC 25586 Fusobacterium nucleatum subsp. vincentii 3\_1\_36A2 Fusobacterium nucleatum subsp. animalis 4\_8 Ilyobacter polytropus Leptotrichia buccalis Sebaldella termitidis Streptobacillus moniliformis Gemmatimonas aurantiaca Gemmatimonadetes bacterium KBS708 Thermanaerovibrio acidaminovorans Aminobacterium colombiense Thermovirga lienii Anaerobaculum mobile Rhodopirellula baltica Pirellula staleyi Planctomyces limnophilus Planctomyces brasiliensis Isosphaera pallida Singulisphaera acidiphila Phycisphaera mikurensis Synechocystis sp. PCC 6803 Synechocystis sp. PCC 6803 Synechocystis sp. PCC 6803 GT-S Synechocystis sp. PCC 6803 GT-I Synechocystis sp. PCC 6803 PCC-N Synechocystis sp. PCC 6803 PCC-P Synechococcus sp. WH8102 Synechococcus elongatus PCC6301 Synechococcus elongatus PCC7942 Synechococcus sp. CC9605 Synechococcus sp. CC9902 Synechococcus sp. CC9311 Synechococcus sp. RCC307 Synechococcus sp. PCC7002 Synechococcus sp. JA-3-3Ab Synechococcus sp. JA-2-3B'a(2-13) Synechococcus sp. PCC 6312 Synechococcus sp. PCC 7502 Thermosynechococcus elongatus Thermosynechococcus sp. NK55 Microcystis aeruginosa Cyanothece sp. ATCC 51142 Cyanothece sp. PCC 8801 Cyanothece sp. PCC 7424 Cyanothece sp. PCC 7425 Cyanothece sp. PCC 8802 Cyanothece sp. PCC 7822 Acaryochloris marina Cyanobium gracile Cyanobacterium aponinum Cyanobacterium stanieri Dactylococcopsis salina Halothece sp. PCC 7418 Gloeocapsa sp. PCC 7428 Chamaesiphon minutus Trichodesmium erythraeum Leptolyngbya sp. PCC 7376 Geitlerinema sp. PCC 7407 Oscillatoria acuminata Oscillatoria nigro-viridis Pseudanabaena sp. PCC 7367 Crinalium epipsammum Microcoleus sp. PCC 7113 Arthrospira platensis Gloeobacter violaceus Gloeobacter kilaueensis Nostoc sp. PCC 7120 Nostoc punctiforme Nostoc sp. PCC 7107 Nostoc sp. PCC 7524 Anabaena variabilis Anabaena sp. 90 Anabaena cylindrica Anabaena azollae 0708 Cylindrospermum stagnale Calothrix sp. PCC 7507 Calothrix sp. PCC 6303 Rivularia sp. PCC 7116 Prochlorococcus marinus SS120 Prochlorococcus marinus MIT 9313 Prochlorococcus marinus MIT9312 Prochlorococcus marinus AS9601 Prochlorococcus marinus MIT 9303 Prochlorococcus marinus MIT 9301 Prochlorococcus marinus MIT 9215 Chroococcidiopsis thermalis Pleurocapsa sp. PCC 7327 Stanieria cyanosphaera Bacteroides thetaiotaomicron Bacteroides fragilis YCH46 Bacteroides fragilis NCTC9343 Bacteroides fragilis 638R Bacteroides vulgatus Bacteroides helcogenes Bacteroides salanitronis Bacteroides xylanisolvens Bacteroides sp. CF50 Porphyromonas gingivalis W83 Porphyromonas gingivalis ATCC 33277 Porphyromonas gingivalis TDC60 Porphyromonas asaccharolytica Parabacteroides distasonis Paludibacter propionicigenes Odoribacter splanchnicus Tannerella forsythia Barnesiella viscericola Candidatus Azobacteroides pseudotrichonymphae (Coptotermes formosanus) Prevotella ruminicola Prevotella melaninogenica Prevotella denticola Prevotella intermedia Prevotella dentalis Prevotella sp. oral taxon 299 Alistipes finegoldii Alistipes shahii Rikenellaceae bacterium Draconibacterium orientale Salinibacter ruber DSM 13855 Salinibacter ruber M8 Rhodothermus marinus DSM 4252 Rhodothermus marinus SG0.5JP17-172 Chitinophaga pinensis Niastella koreensis Pedobacter heparinus Pedobacter saltans Sphingobacterium sp. 21 Solitalea canadensis Haliscomenobacter hydrossis Saprospira grandis Cyclobacterium marinum Belliella baltica Echinicola vietnamensis Cytophaga hutchinsonii Dyadobacter fermentans Spirosoma linguale Leadbetterella byssophila Runella slithyformis Flexibacter litoralis Emticicia oligotrophica Fibrella aestuarina Hymenobacter swuensis Hymenobacter sp. APR13 Marivirga tractuosa Candidatus Amoebophilus asiaticus Cardinium endosymbiont cEper1 (Encarsia pergandiella) Gramella forsetii Flavobacterium johnsoniae Flavobacterium psychrophilum JIP02/86 Flavobacterium branchiophilum Flavobacterium columnare Flavobacterium indicum Capnocytophaga canimorsus Robiginitalea biformata Zunongwangia profunda Croceibacter atlanticus Riemerella anatipestifer ATCC 11845 = DSM 15868 Riemerella anatipestifer ATCC 11845 = DSM 15868 Riemerella anatipestifer RA-GD Riemerella anatipestifer RA-CH-1 Riemerella anatipestifer RA-CH-2 Maribacter sp. HTCC2170 Cellulophaga algicola Cellulophaga lytica Weeksella virosa Krokinobacter sp. 4H-3-7-5 Lacinutrix sp. 5H-3-7-4 Zobellia galactanivorans Muricauda ruestringensis Aequorivita sublithincola Ornithobacterium rhinotracheale Psychroflexus torquis Nonlabens dokdonensis Polaribacter sp. MED152 Flavobacteriaceae bacterium Blattabacterium Bge (Blattella germanica) Blattabacterium BPLAN (Periplaneta americana) Blattabacterium MADAR (Mastotermes darwiniensis) Blattabacterium Cpu (Cryptocercus punctulatus) Blattabacterium BGIGA (Blaberus giganteus) Blattabacterium sp. (Blatta orientalis) Blattabacterium sp. (Panesthia angustipennis spadica) Blattabacterium sp. (Nauphoeta cinerea) Fluviicola taffensis Owenweeksia hongkongensis Chlorobium tepidum Chlorobaculum parvum Chlorobium chlorochromatii Chlorobium phaeobacteroides DSM 266 Chlorobium phaeobacteroides BS1 Chlorobium limicola Chlorobium phaeovibrioides Pelodictyon luteolum Pelodictyon phaeoclathratiforme Prosthecochloris aestuarii Chloroherpeton thalassium Ignavibacterium album Melioribacter roseus Dehalococcoides mccartyi 195 Dehalococcoides mccartyi CBDB1 Dehalococcoides mccartyi BAV1 Dehalococcoides mccartyi VS Dehalococcoides mccartyi GT Dehalococcoides mccartyi BTF08 Dehalococcoides mccartyi DCMB5 Dehalococcoides mccartyi GY50 Dehalogenimonas lykanthroporepellens Roseiflexus sp. RS-1 Roseiflexus castenholzii Chloroflexus aurantiacus Chloroflexus aggregans Chloroflexus sp. Y-400-fl Herpetosiphon aurantiacus Thermomicrobium roseum Sphaerobacter thermophilus Anaerolinea thermophila Caldilinea aerophila Deinococcus radiodurans Deinococcus geothermalis Deinococcus deserti Deinococcus maricopensis Deinococcus proteolyticus Deinococcus gobiensis Deinococcus peraridilitoris Truepera radiovictrix Thermus thermophilus HB27 Thermus thermophilus HB8 Thermus thermophilus SG0.5JP17-16 Thermus thermophilus JL-18 Thermus scotoductus Thermus sp. CCB\_US3\_UF1 Thermus oshimai Meiothermus ruber DSM 1279 Meiothermus ruber DSM 1279 Meiothermus silvanus Oceanithermus profundus Marinithermus hydrothermalis Aquifex aeolicus Hydrogenobaculum sp. Y04AAS1 Hydrogenobaculum sp. HO Hydrogenobaculum sp. SN Hydrogenobacter thermophilus Hydrogenobacter thermophilus Thermocrinis albus Thermocrinis ruber Sulfurihydrogenibium sp. YO3AOP1 Sulfurihydrogenibium azorense Persephonella marina Thermovibrio ammonificans Desulfurobacterium thermolithotrophum Thermotoga maritima Thermotoga maritima Thermotoga maritima Thermotoga petrophila Thermotoga lettingae Thermotoga sp. RQ2 Thermotoga neapolitana Thermotoga naphthophila Thermotoga thermarum Thermosipho melanesiensis Thermosipho africanus Fervidobacterium nodosum Fervidobacterium pennivorans Petrotoga mobilis Kosmotoga olearia Marinitoga piezophila Mesotoga prima Chthonomonas calidirosea Fimbriimonas ginsengisoli Caldisericum exile Desulfurispirillum indicum Deferribacter desulfuricans Denitrovibrio acetiphilus Calditerrivibrio nitroreducens Flexistipes sinusarabici Dictyoglomus thermophilum Dictyoglomus turgidum Thermodesulfovibrio yellowstonii Candidatus Nitrospira defluvii Leptospirillum ferrooxidans Leptospirillum ferriphilum ML-04 Leptospirillum ferriphilum YSK Thermodesulfatator indicus Thermodesulfobacterium geofontis Thermodesulfobacterium commune Thermobaculum terrenum Candidatus Methylomirabilis oxyfera Halyomorpha halys symbiont Candidate division SR1 bacterium RAAC1\_SR1\_1 Methanocaldococcus jannaschii Methanocaldococcus fervens Methanocaldococcus vulcanius Methanocaldococcus sp. FS406-22 Methanocaldococcus infernus Methanotorris igneus Methanococcus maripaludis S2 Methanococcus maripaludis C5 Methanococcus maripaludis C6 Methanococcus maripaludis C7 Methanococcus maripaludis X1 Methanococcus aeolicus Methanococcus vannielii Methanococcus voltae Methanothermococcus okinawensis Methanosarcina acetivorans Methanosarcina barkeri Methanosarcina mazei Go1 Methanosarcina mazei Tuc01 Methanococcoides burtonii Methanohalophilus mahii Methanohalobium evestigatum Methanosalsum zhilinae Methanolobus psychrophilus Methanomethylovorans hollandica Methanosaeta thermophila Methanosaeta concilii Methanosaeta harundinacea Methanospirillum hungatei Methanocorpusculum labreanum Methanoculleus marisnigri Methanoculleus bourgensis Methanoplanus petrolearius Methanoregula boonei Methanoregula formicica Candidatus Methanosphaerula palustris Methanocella paludicola Methanocella conradii Methanocella arvoryzae Methanomassiliicoccus sp. Mx1-Issoire Methanothermobacter thermautotrophicus Methanothermobacter marburgensis Methanosphaera stadtmanae Methanobrevibacter smithii Methanobrevibacter ruminantium Methanobrevibacter sp. AbM4 Methanobacterium lacus Methanobacterium sp. SWAN-1 Methanobacterium sp. MB1 Methanothermus fervidus Methanopyrus kandleri Candidatus Methanomethylophilus alvus Archaeoglobus fulgidus DSM 4304 Archaeoglobus fulgidus DSM 8774 Archaeoglobus profundus Archaeoglobus veneficus Archaeoglobus sulfaticallidus Ferroglobus placidus Halobacterium sp. NRC-1 Halobacterium salinarum Haloarcula marismortui Haloarcula hispanica ATCC 33960 Haloarcula hispanica N601 Haloquadratum walsbyi DSM 16790 Haloquadratum walsbyi C23 Natronomonas pharaonis Natronomonas moolapensis Halorubrum lacusprofundi Halorhabdus utahensis Halorhabdus tiamatea Halomicrobium mukohataei Haloterrigena turkmenica Natrialba magadii Haloferax volcanii Haloferax mediterranei Halalkalicoccus jeotgali Halogeometricum borinquense Halopiger xanaduensis Natrinema sp. J7-2 Natrinema pellirubrum Natronobacterium gregoryi Halovivax ruber Natronococcus occultus Salinarchaeum sp. Harcht-Bsk1 Halostagnicola larsenii Thermoplasma acidophilum Thermoplasma volcanium Picrophilus torridus Ferroplasma acidarmanus Thermoplasmatales archaeon BRNA1 Pyrococcus horikoshii Pyrococcus abyssi Pyrococcus furiosus DSM 3638 Pyrococcus furiosus COM1 Pyrococcus sp. NA2 Pyrococcus yayanosii Pyrococcus sp. ST04 Thermococcus kodakarensis Thermococcus onnurineus Thermococcus gammatolerans Thermococcus sibiricus Thermococcus barophilus Thermococcus sp. 4557 Thermococcus sp. AM4 Thermococcus sp. CL1 Thermococcus litoralis Thermococcus sp. ES1 Thermococcus nautili Palaeococcus pacificus Aciduliprofundum boonei Aciduliprofundum sp. MAR08-339 Aeropyrum pernix Aeropyrum camini Staphylothermus marinus Staphylothermus hellenicus Ignicoccus hospitalis Desulfurococcus kamchatkensis Desulfurococcus mucosus Desulfurococcus fermentans Thermosphaera aggregans Ignisphaera aggregans Thermogladius cellulolyticus Hyperthermus butylicus Pyrolobus fumarii Sulfolobus solfataricus P2 Sulfolobus solfataricus 98/2 Sulfolobus tokodaii Sulfolobus acidocaldarius DSM 639 Sulfolobus acidocaldarius N8 Sulfolobus acidocaldarius Ron12/I Sulfolobus acidocaldarius SUSAZ Sulfolobus islandicus L.S.2.15 Sulfolobus islandicus M.14.25 Sulfolobus islandicus M.16.27 Sulfolobus islandicus M.16.4 Sulfolobus islandicus Y.G.57.14 Sulfolobus islandicus Y.N.15.51 Sulfolobus islandicus L.D.8.5 Sulfolobus islandicus HVE10/4 Sulfolobus islandicus REY15A Sulfolobus islandicus LAL14/1 Metallosphaera sedula Metallosphaera cuprina Acidianus hospitalis Pyrobaculum aerophilum Pyrobaculum islandicum Pyrobaculum calidifontis Pyrobaculum arsenaticum Pyrobaculum sp. 1860 Pyrobaculum oguniense Pyrobaculum neutrophilum Caldivirga maquilingensis Thermoproteus uzoniensis Thermoproteus tenax Vulcanisaeta distributa Vulcanisaeta moutnovskia Thermofilum pendens Thermofilum sp. 1910b Acidilobus saccharovorans Caldisphaera lagunensis Fervidicoccus fontis Nitrosopumilus maritimus Candidatus Nitrosopumilus sp. AR2 Candidatus Nitrosopumilus koreensis Cenarchaeum symbiosum Candidatus Nitrososphaera gargensis Nitrososphaera viennensis Candidatus Nitrososphaera evergladensis Candidatus Caldiarchaeum subterraneum Candidatus Korarchaeum cryptofilum Halophilic archaeon | 184% 150% 122% 100% 82% 67% 55% |
