# Supplementary material for: Global analysis of lysine acetylation in strawberry leaves
Source: Front Plant Sci. 2015 Sep 15;6:739. doi: 10.3389/fpls.2015.00739 (PMC4569977; doi:10.3389/fpls.2015.00739)
Supplement: Supplementary file 5 [file DataSheet1.ZIP › Photosynthesis - Fragaria vesca (woodland strawberry).html]

KEGG PATHWAY: Photosynthesis - Fragaria vesca (woodland strawberry)


|  |  |
| --- | --- |
| **Photosynthesis - Fragaria vesca (woodland strawberry)** |  |

[
Pathway menu
| Organism menu
| Pathway entry
| Download KGML
| Show description
| User data mapping
]

|  |
| --- |
| Photosynthesis in green plants and specialized bacteria is the process of utilizing light energy to synthesize organic compounds from carbon dioxide and water. It consists of the light dependent part (light reaction) and the light independent part (dark reaction, carbon fixation). The light reaction takes place in thylakoid, a membrane-bound compartment inside chloroplasts and cyanobacteria. The light energy is used by photosystems I and II to generate proton motive force and reducing power (NADPH or NADH). The proton motive force is used by ATP synthase to generate ATP, essentially in the same way as the mitochondrial respiratory chain. The supplies of ATP and NAD(P)H are then used to fix carbon dioxide. |

|  |  |
| --- | --- |
| Reference pathway Reference pathway (KO) Reference pathway (EC) -----< Set personalized menu >----- -----< Sort below by alphabet >----- Arabidopsis thaliana (thale cress) Arabidopsis lyrata (lyrate rockcress) Capsella rubella Eutrema salsugineum Citrus sinensis (Valencia orange) Citrus clementina (mandarin orange) Theobroma cacao (cacao) Glycine max (soybean) Phaseolus vulgaris (common bean) Medicago truncatula (barrel medic) Cicer arietinum (chickpea) Fragaria vesca (woodland strawberry) Prunus persica (peach) Prunus mume (Japanese apricot) Malus domestica (apple) Cucumis sativus (cucumber) Cucumis melo (muskmelon) Ricinus communis (castor bean) Populus trichocarpa (black cottonwood) Vitis vinifera (wine grape) Solanum lycopersicum (tomato) Solanum tuberosum (potato) Oryza sativa japonica (Japanese rice) (RefSeq) Oryza sativa japonica (Japanese rice) (RAPDB) Oryza brachyantha (malo sina) Brachypodium distachyon Sorghum bicolor (sorghum) Zea mays (maize) Setaria italica (foxtail millet) Amborella trichopoda Selaginella moellendorffii Physcomitrella patens subsp. patens Chlamydomonas reinhardtii Volvox carteri f. nagariensis Ostreococcus lucimarinus Ostreococcus tauri Bathycoccus prasinos Micromonas sp. RCC299 Micromonas pusilla Coccomyxa subellipsoidea Chlorella variabilis Cyanidioschyzon merolae Galdieria sulphuraria Chondrus crispus (carragheen) Phaeodactylum tricornutum Thalassiosira pseudonana Synechocystis sp. PCC 6803 Synechocystis sp. PCC 6803 Synechocystis sp. PCC 6803 GT-S Synechocystis sp. PCC 6803 GT-I Synechocystis sp. PCC 6803 PCC-N Synechocystis sp. PCC 6803 PCC-P Synechococcus sp. WH8102 Synechococcus elongatus PCC6301 Synechococcus elongatus PCC7942 Synechococcus sp. CC9605 Synechococcus sp. CC9902 Synechococcus sp. CC9311 Synechococcus sp. RCC307 Synechococcus sp. WH7803 Synechococcus sp. PCC7002 Synechococcus sp. JA-3-3Ab Synechococcus sp. JA-2-3B'a(2-13) Synechococcus sp. PCC 6312 Synechococcus sp. PCC 7502 Thermosynechococcus elongatus Thermosynechococcus sp. NK55 Microcystis aeruginosa Cyanothece sp. ATCC 51142 Cyanothece sp. PCC 8801 Cyanothece sp. PCC 7424 Cyanothece sp. PCC 7425 Cyanothece sp. PCC 8802 Cyanothece sp. PCC 7822 Acaryochloris marina Cyanobium gracile Cyanobacterium aponinum Cyanobacterium stanieri Dactylococcopsis salina Halothece sp. PCC 7418 Gloeocapsa sp. PCC 7428 Chamaesiphon minutus Cyanobacterium UCYN-A Trichodesmium erythraeum Leptolyngbya sp. PCC 7376 Geitlerinema sp. PCC 7407 Oscillatoria acuminata Oscillatoria nigro-viridis Pseudanabaena sp. PCC 7367 Crinalium epipsammum Microcoleus sp. PCC 7113 Arthrospira platensis Gloeobacter violaceus Gloeobacter kilaueensis Nostoc sp. PCC 7120 Nostoc punctiforme Nostoc sp. PCC 7107 Nostoc sp. PCC 7524 Anabaena variabilis Anabaena sp. 90 Anabaena cylindrica Anabaena azollae 0708 Cylindrospermum stagnale Calothrix sp. PCC 7507 Calothrix sp. PCC 6303 Rivularia sp. PCC 7116 Prochlorococcus marinus SS120 Prochlorococcus marinus MED4 Prochlorococcus marinus MIT 9313 Prochlorococcus marinus NATL2A Prochlorococcus marinus MIT9312 Prochlorococcus marinus AS9601 Prochlorococcus marinus MIT 9515 Prochlorococcus marinus MIT 9303 Prochlorococcus marinus MIT 9301 Prochlorococcus marinus MIT 9215 Prochlorococcus marinus MIT 9211 Prochlorococcus marinus NATL1A Chroococcidiopsis thermalis Pleurocapsa sp. PCC 7327 Stanieria cyanosphaera | 184% 150% 122% 100% 82% 67% 55% |
