# Supplementary figures and images for: Global analysis of lysine acetylation in strawberry leaves
Source: Front Plant Sci. 2015 Sep 15;6:739. doi: 10.3389/fpls.2015.00739 (PMC4569977; doi:10.3389/fpls.2015.00739)

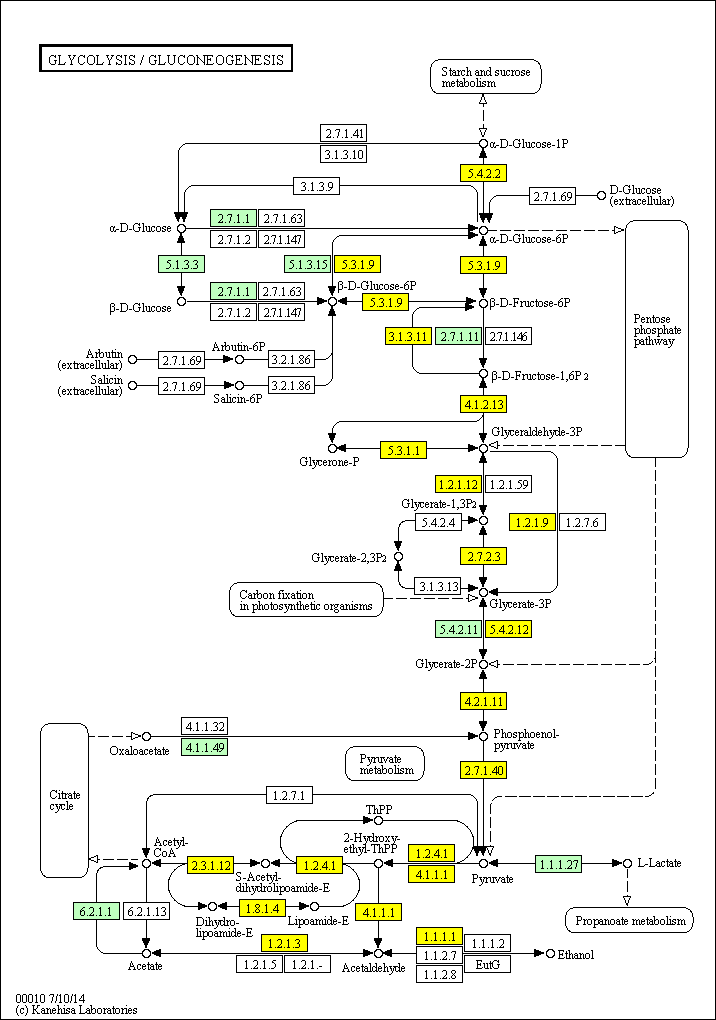

Supplement: Supplementary file 5 [file DataSheet1.ZIP › images/fve00010.png]

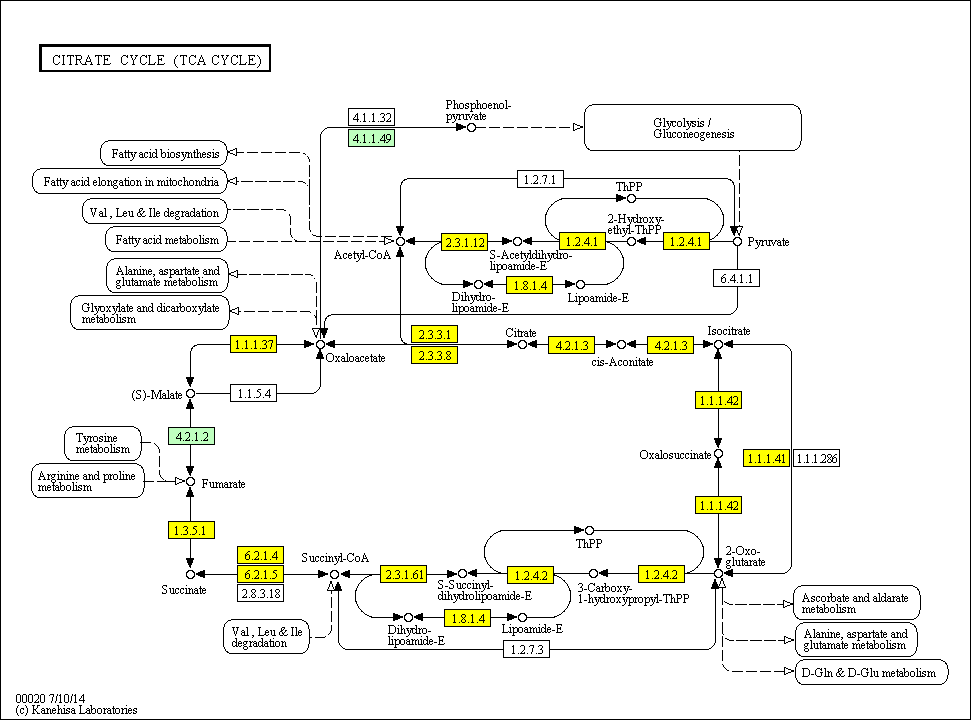

Supplement: Supplementary file 5 [file DataSheet1.ZIP › images/fve00020.png]

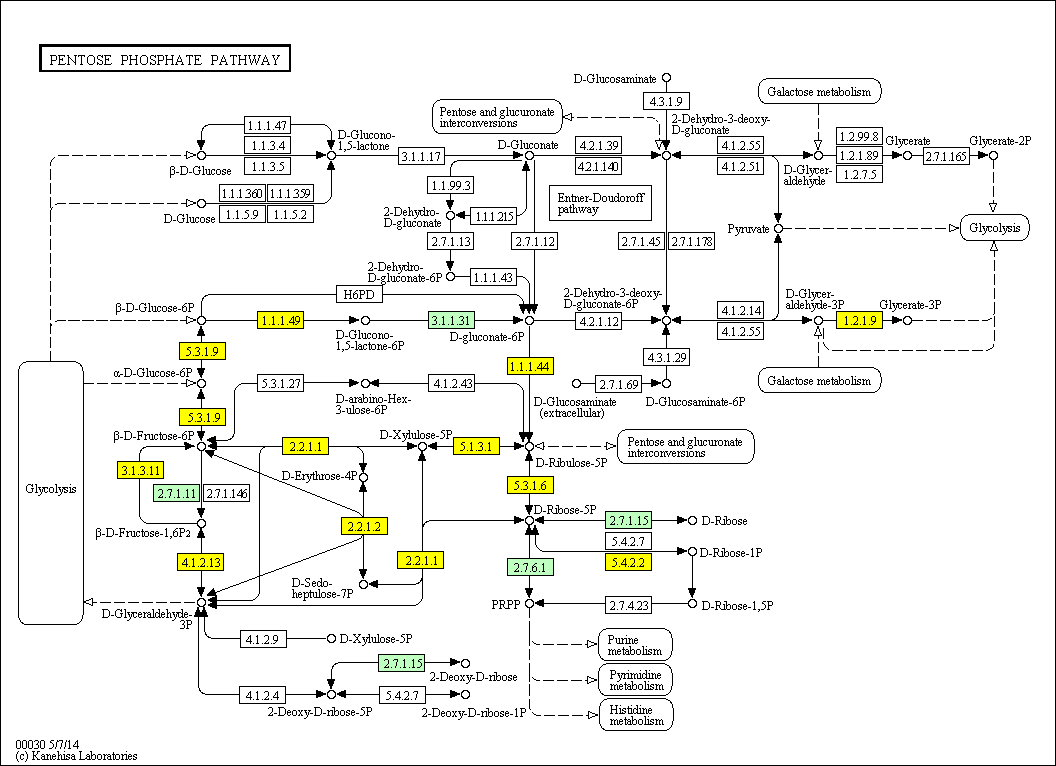

Supplement: Supplementary file 5 [file DataSheet1.ZIP › images/fve00030.png]

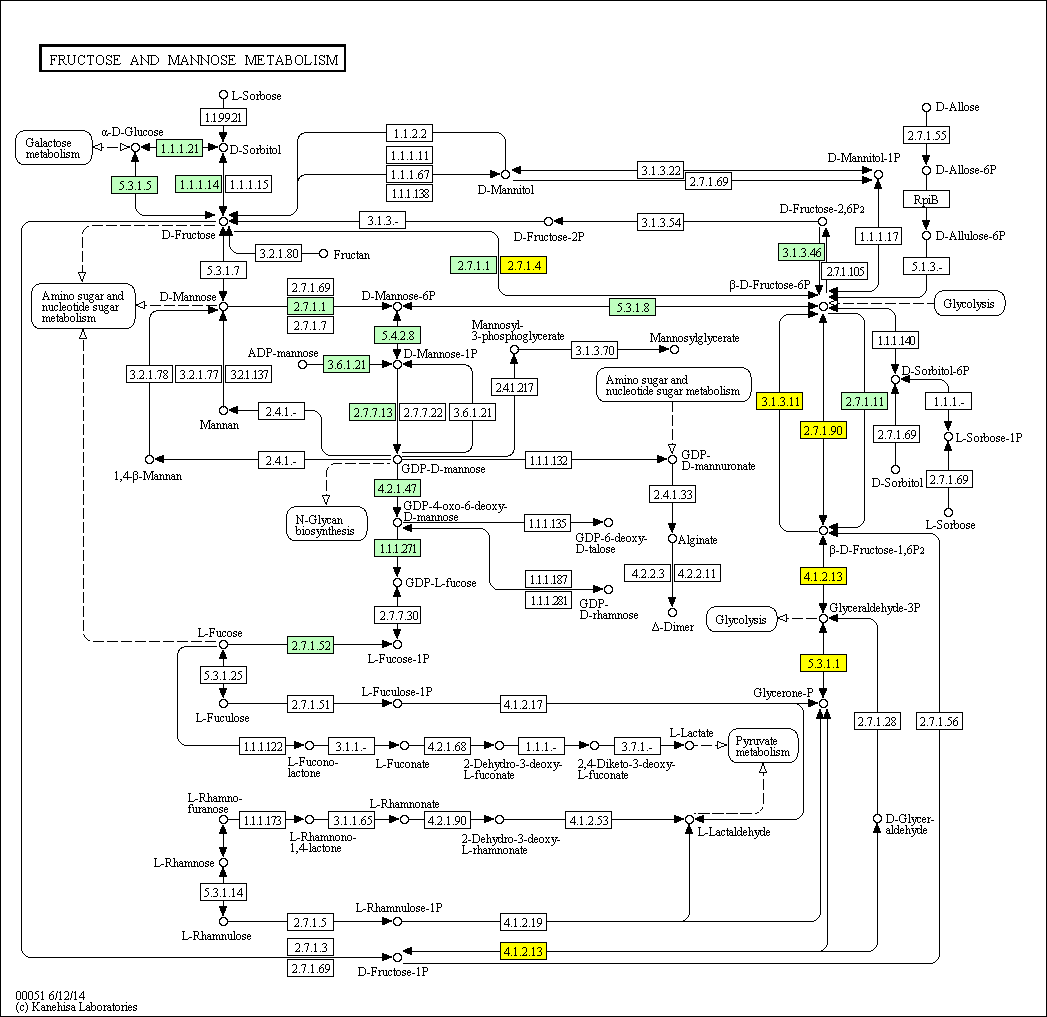

Supplement: Supplementary file 5 [file DataSheet1.ZIP › images/fve00051.png]

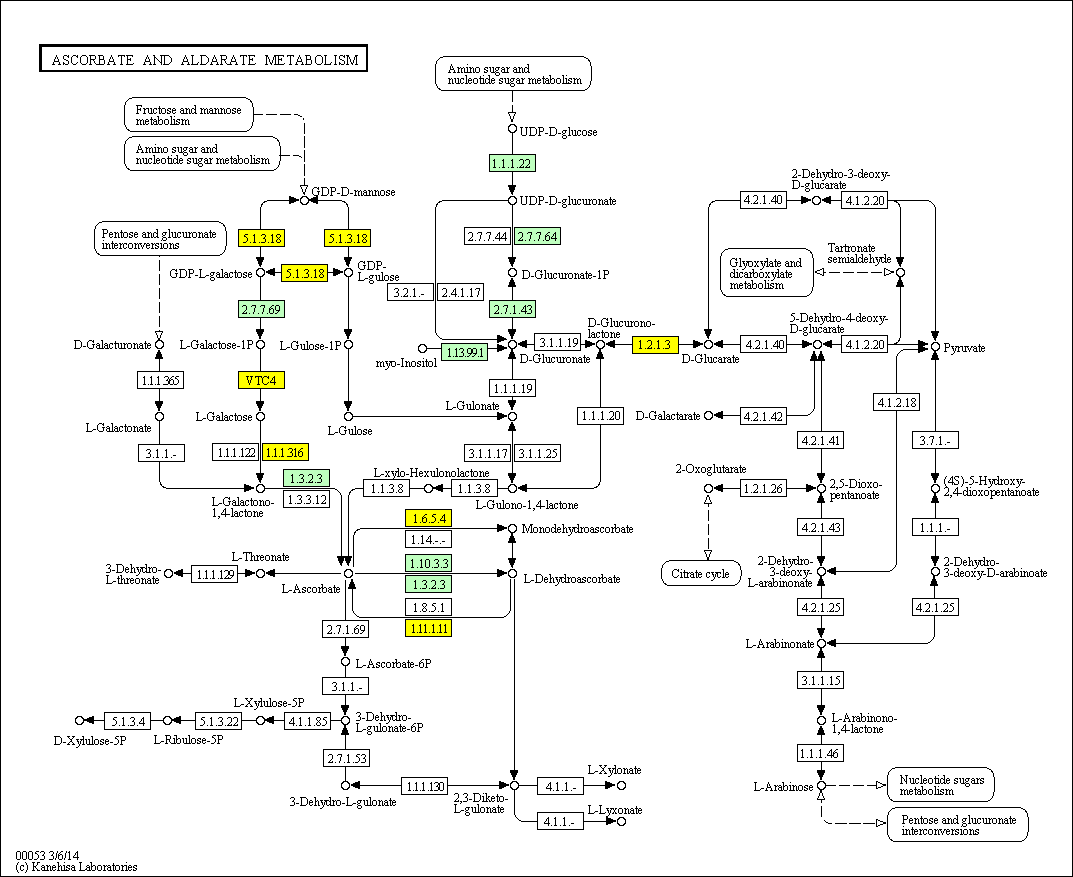

Supplement: Supplementary file 5 [file DataSheet1.ZIP › images/fve00053.png]

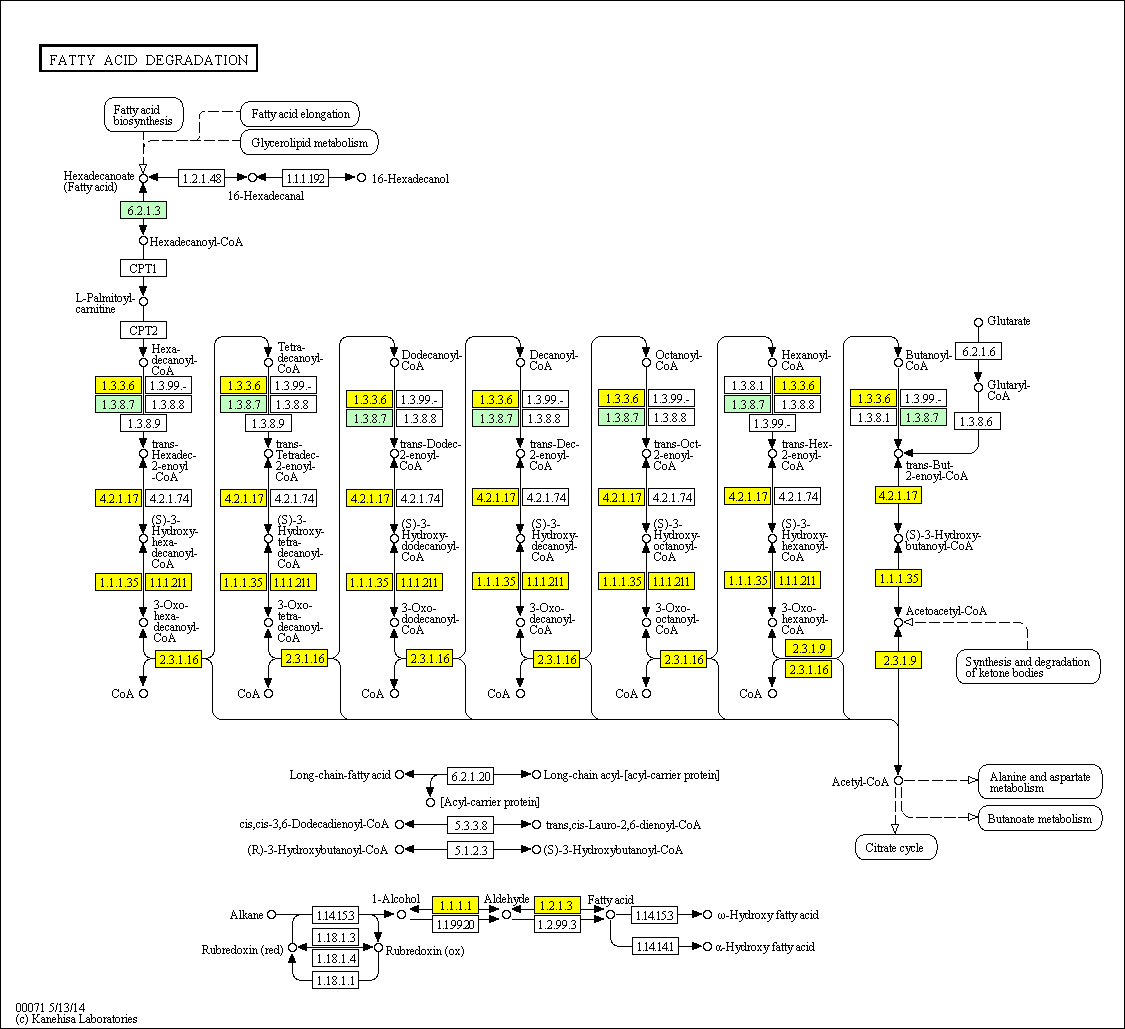

Supplement: Supplementary file 5 [file DataSheet1.ZIP › images/fve00071.png]

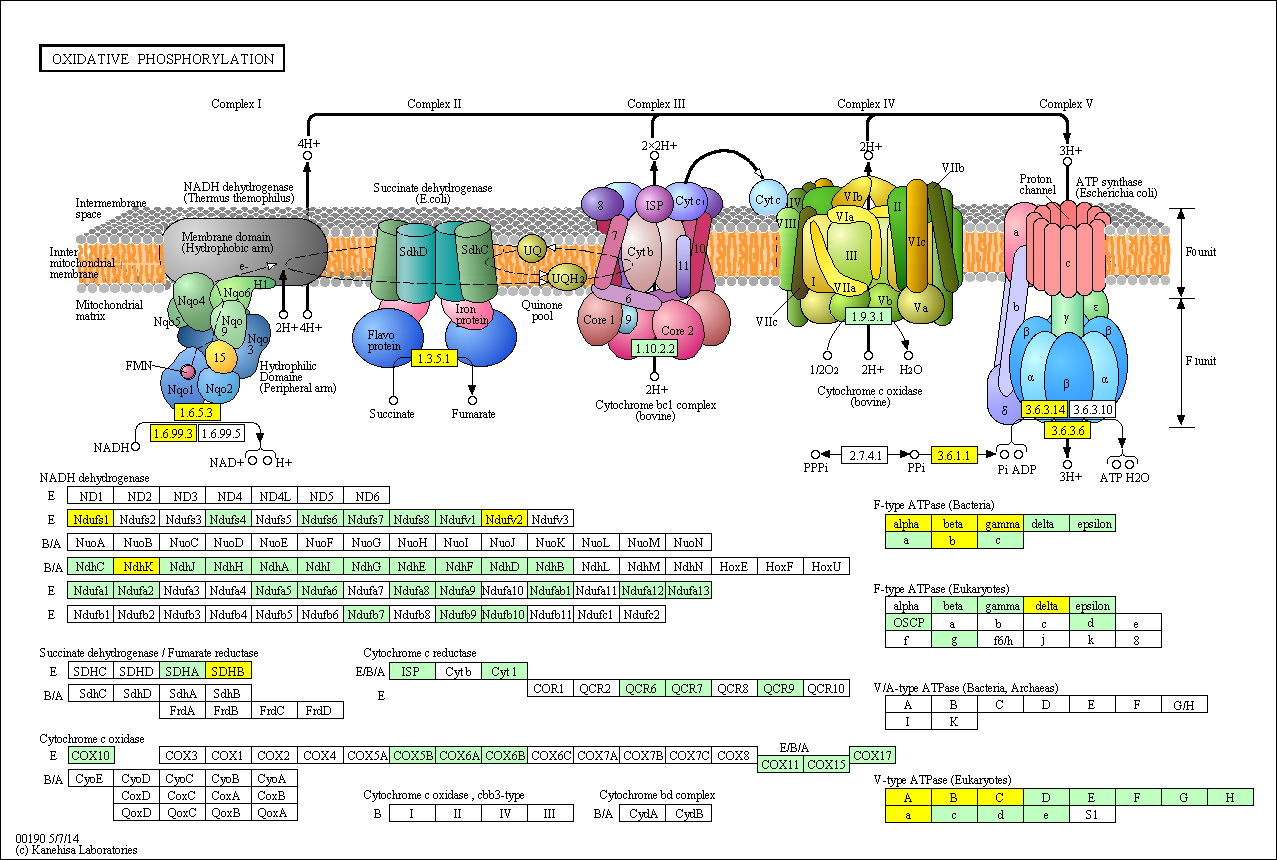

Supplement: Supplementary file 5 [file DataSheet1.ZIP › images/fve00190.png]

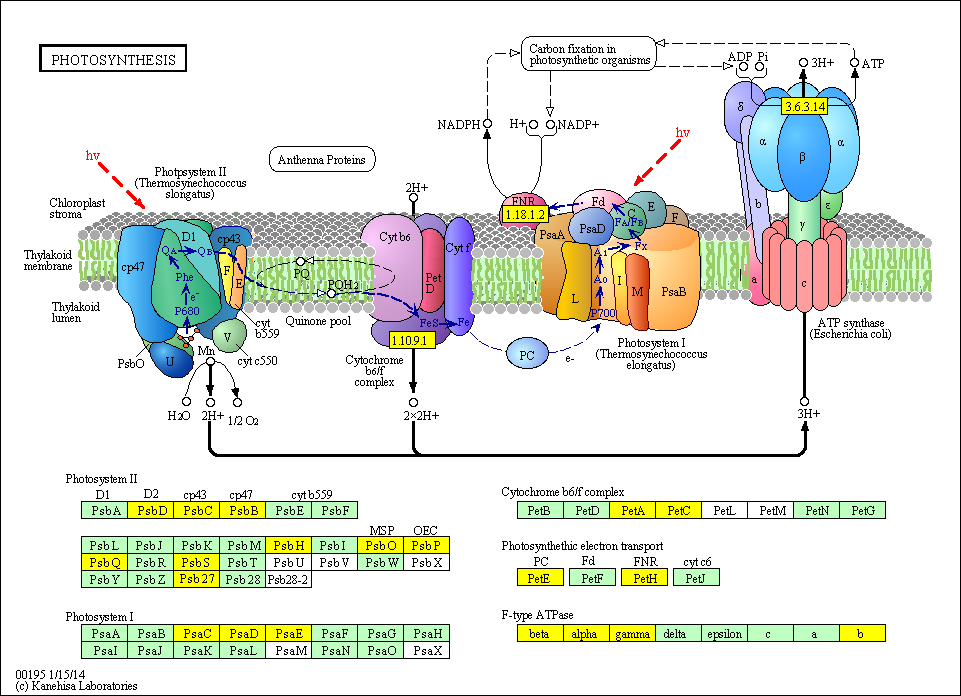

Supplement: Supplementary file 5 [file DataSheet1.ZIP › images/fve00195.png]

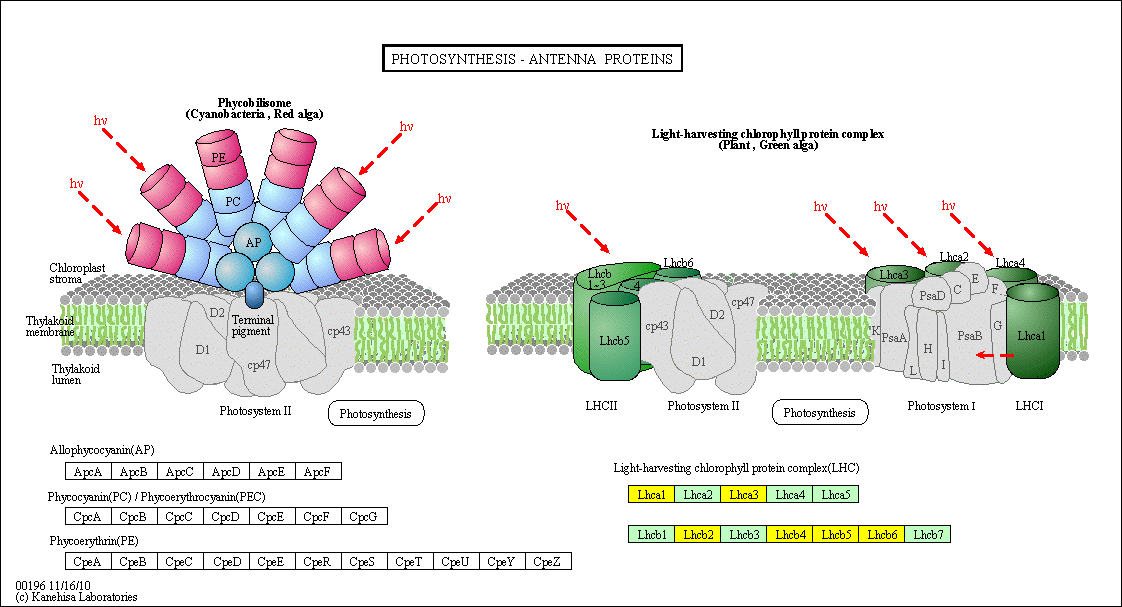

Supplement: Supplementary file 5 [file DataSheet1.ZIP › images/fve00196.png]

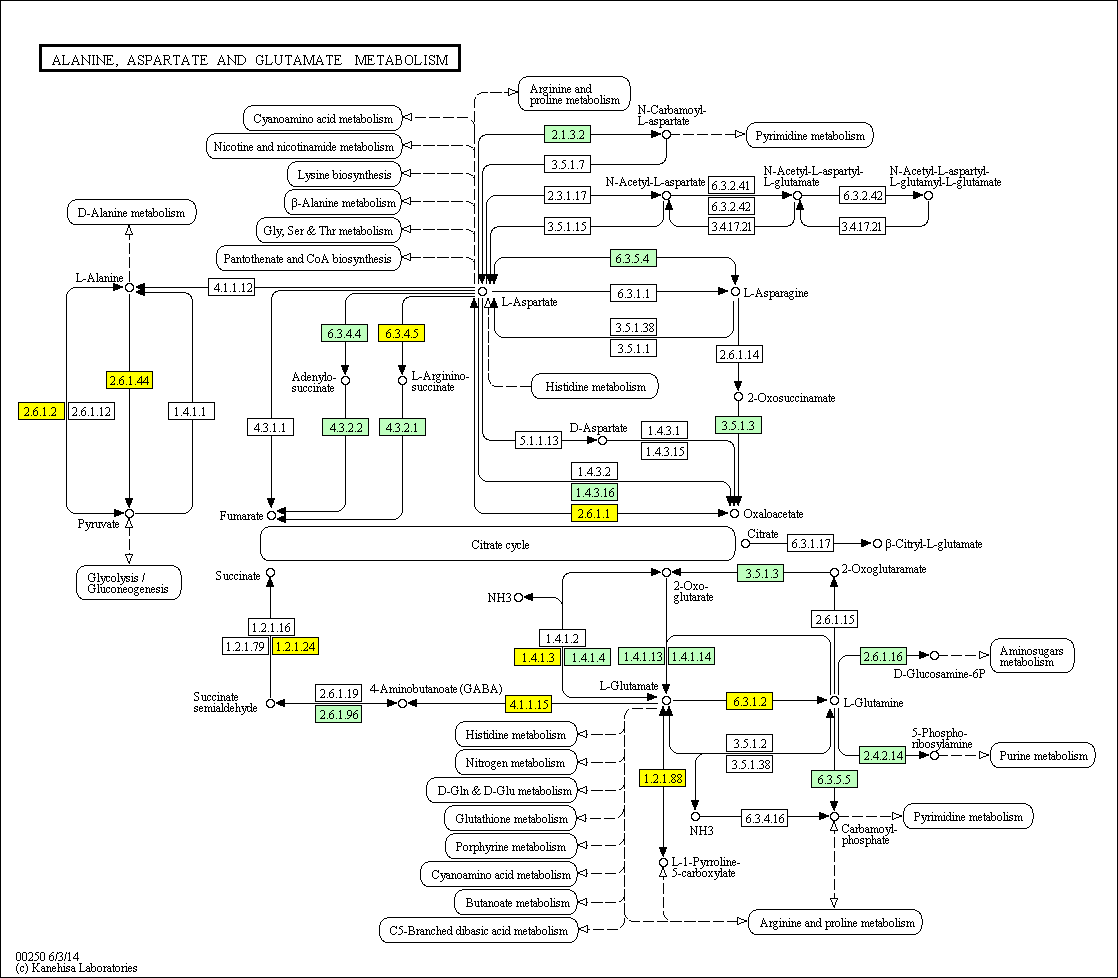

Supplement: Supplementary file 5 [file DataSheet1.ZIP › images/fve00250.png]

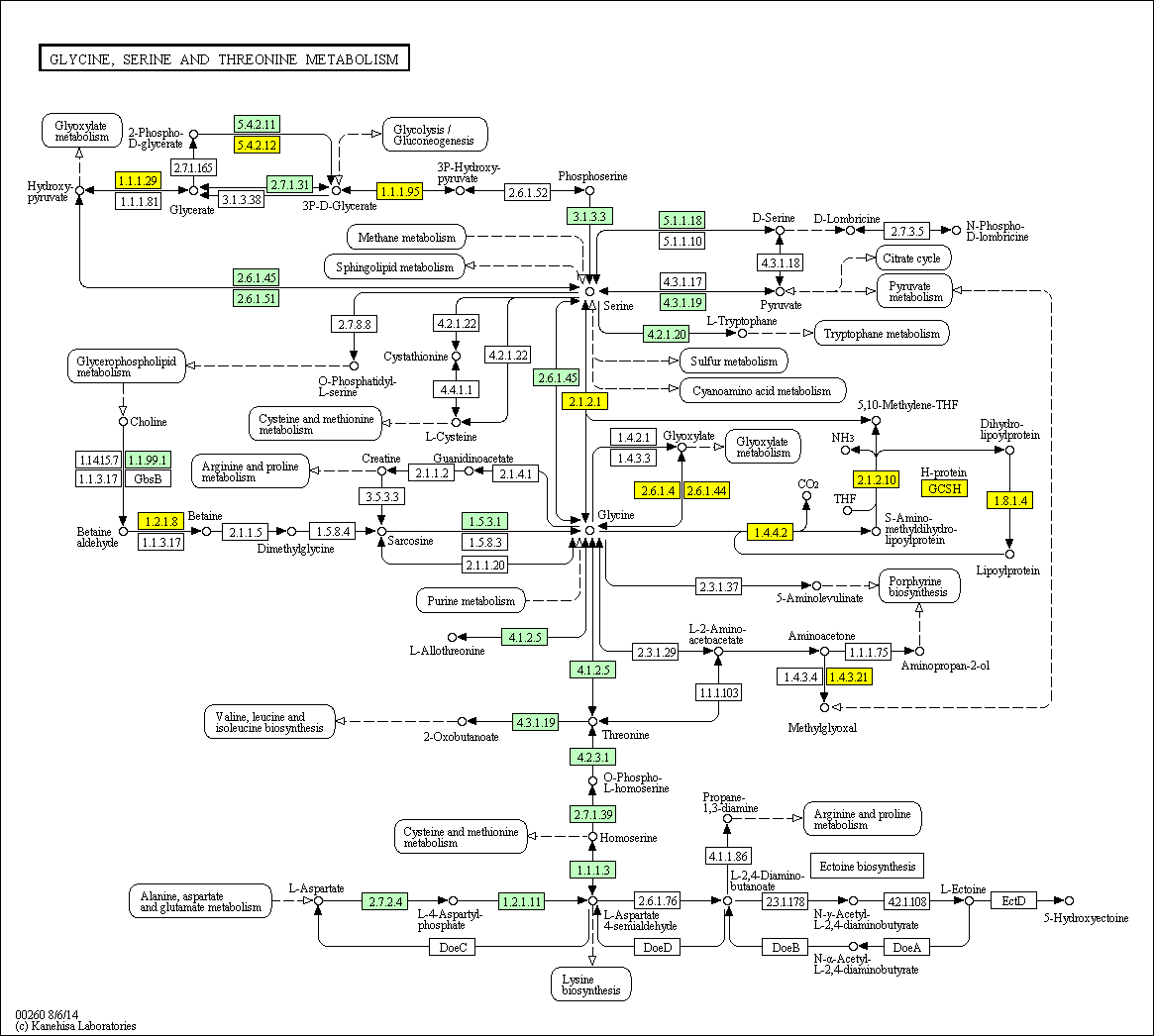

Supplement: Supplementary file 5 [file DataSheet1.ZIP › images/fve00260.png]

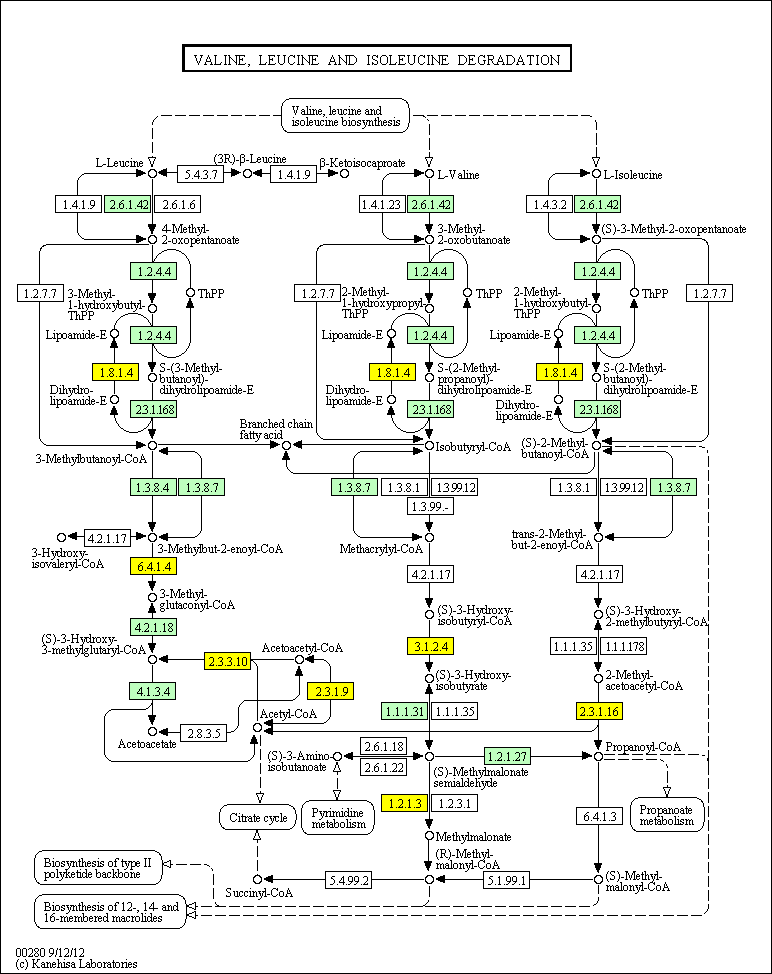

Supplement: Supplementary file 5 [file DataSheet1.ZIP › images/fve00280.png]

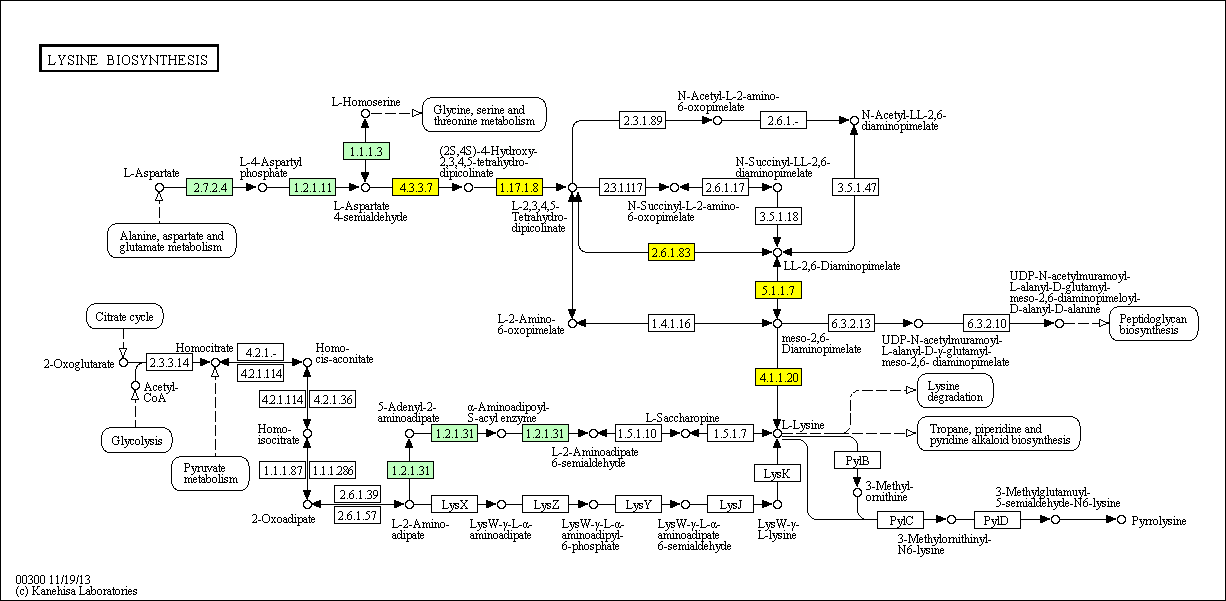

Supplement: Supplementary file 5 [file DataSheet1.ZIP › images/fve00300.png]

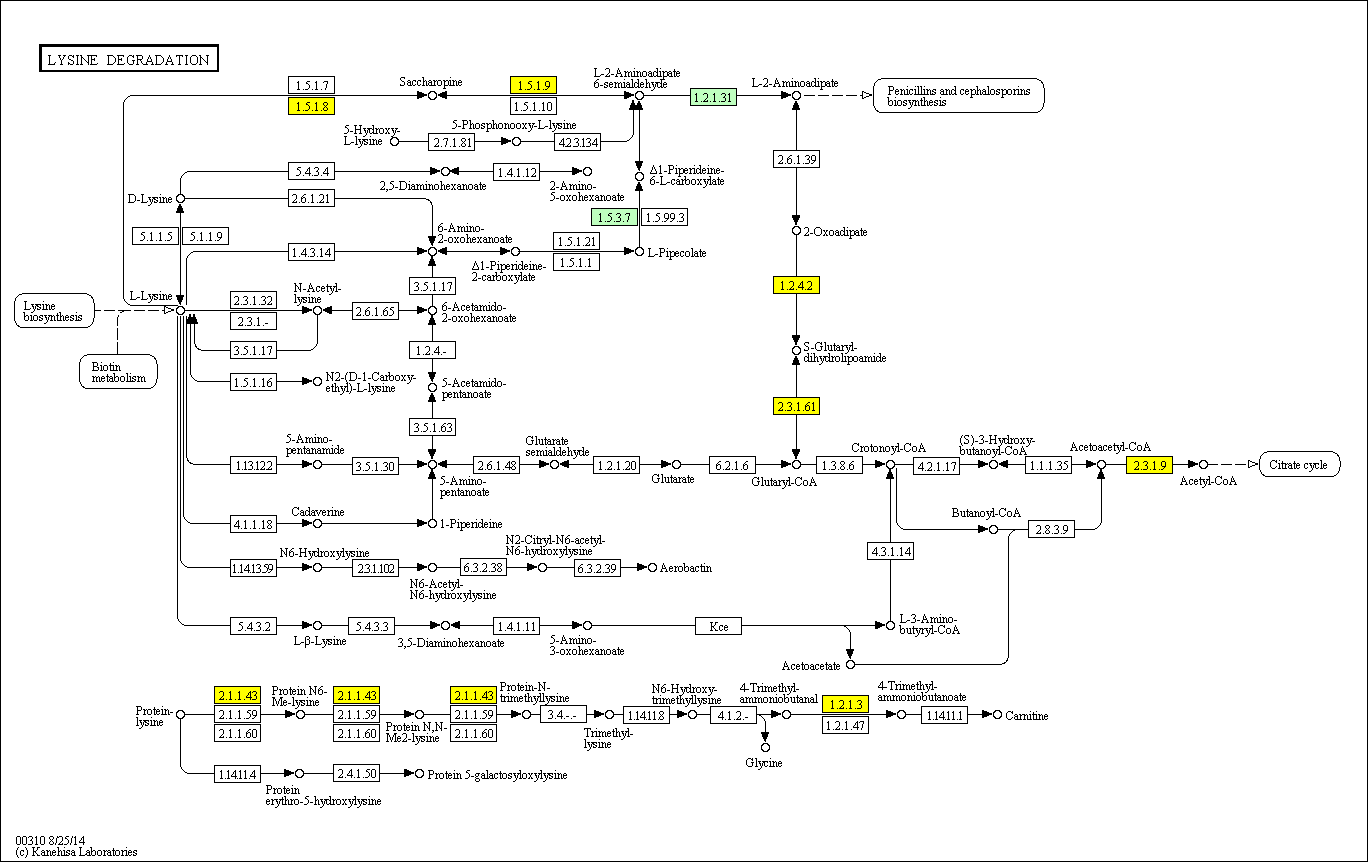

Supplement: Supplementary file 5 [file DataSheet1.ZIP › images/fve00310.png]

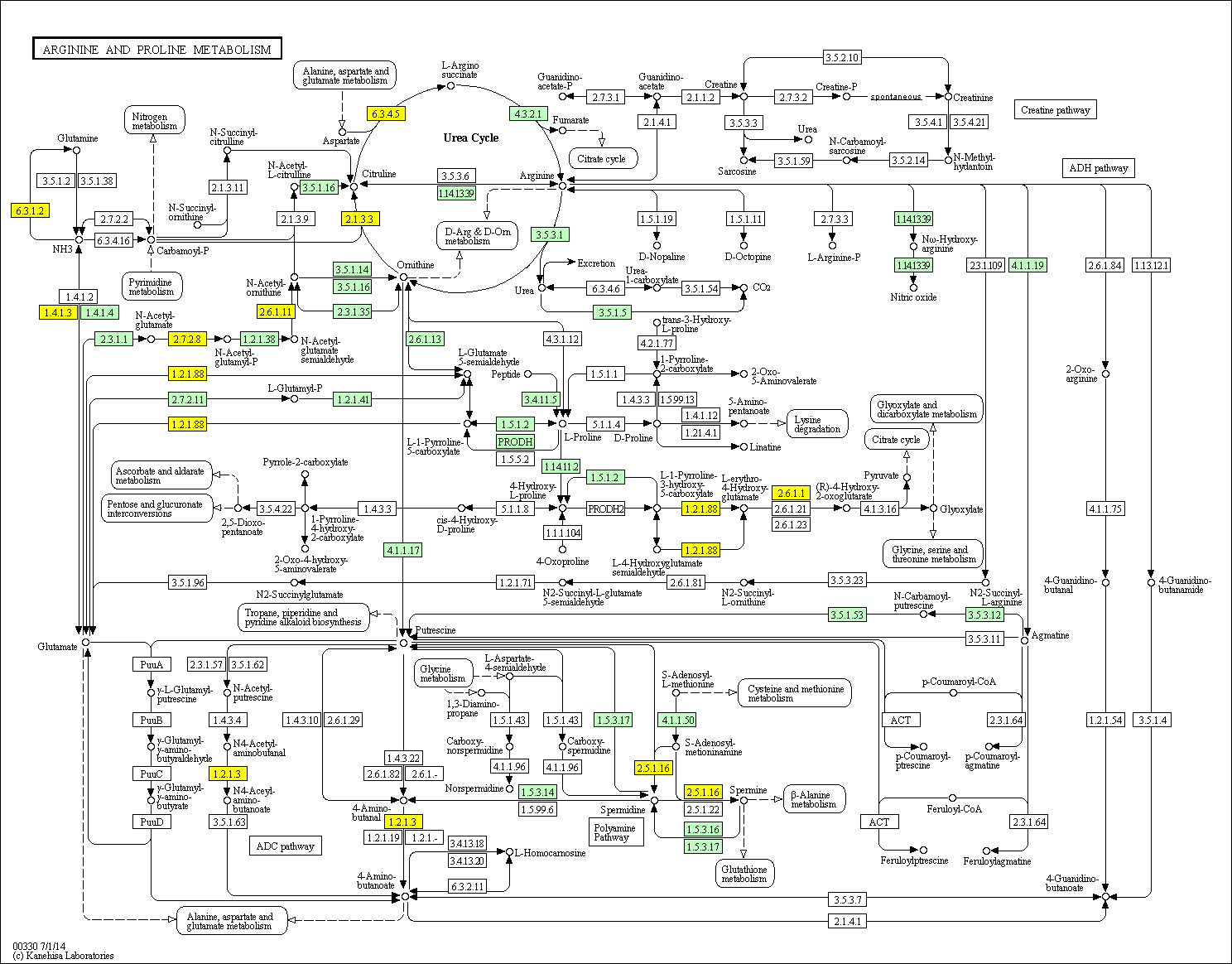

Supplement: Supplementary file 5 [file DataSheet1.ZIP › images/fve00330.png]

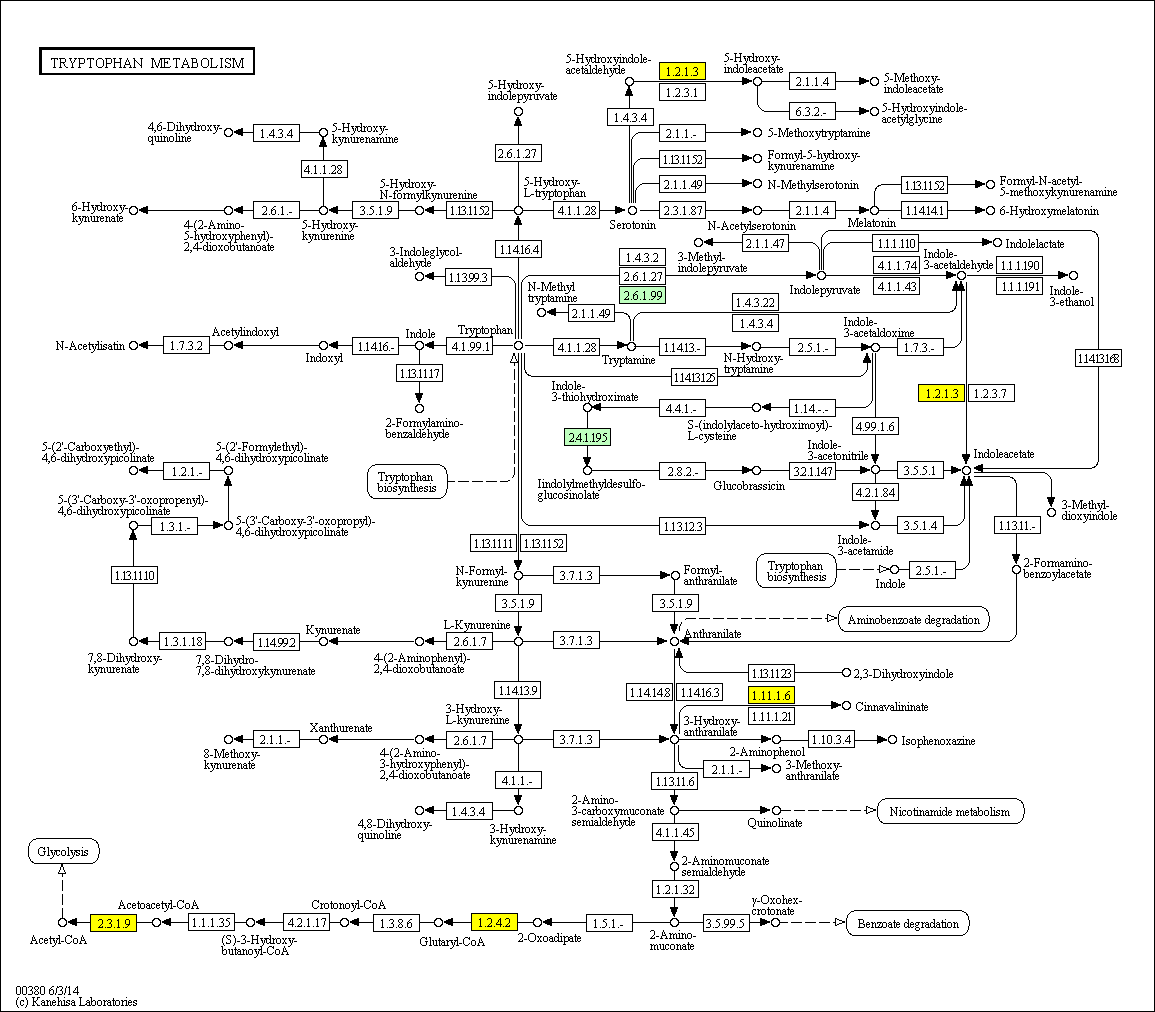

Supplement: Supplementary file 5 [file DataSheet1.ZIP › images/fve00380.png]

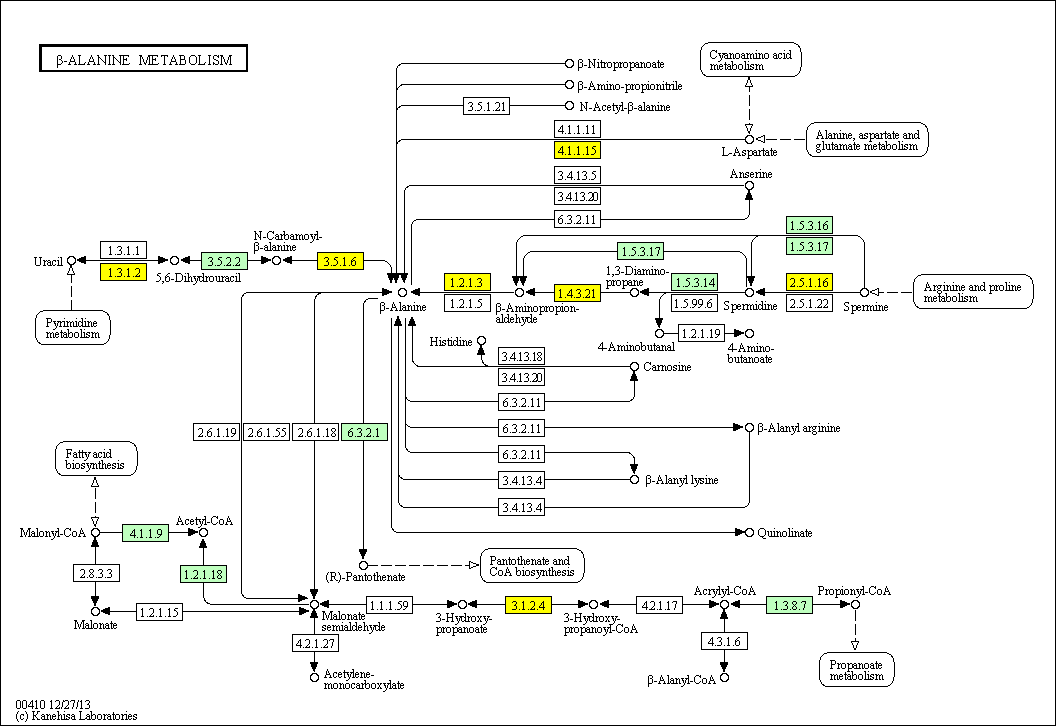

Supplement: Supplementary file 5 [file DataSheet1.ZIP › images/fve00410.png]

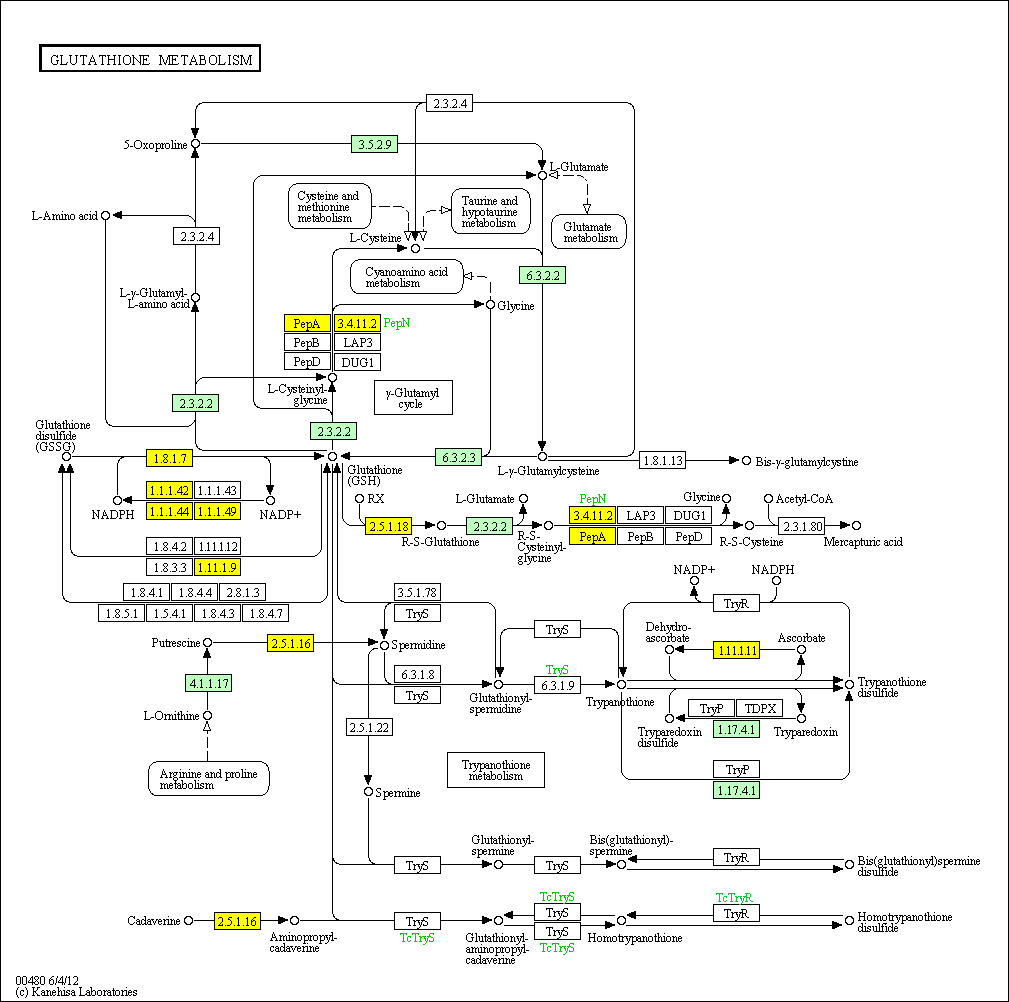

Supplement: Supplementary file 5 [file DataSheet1.ZIP › images/fve00480.png]

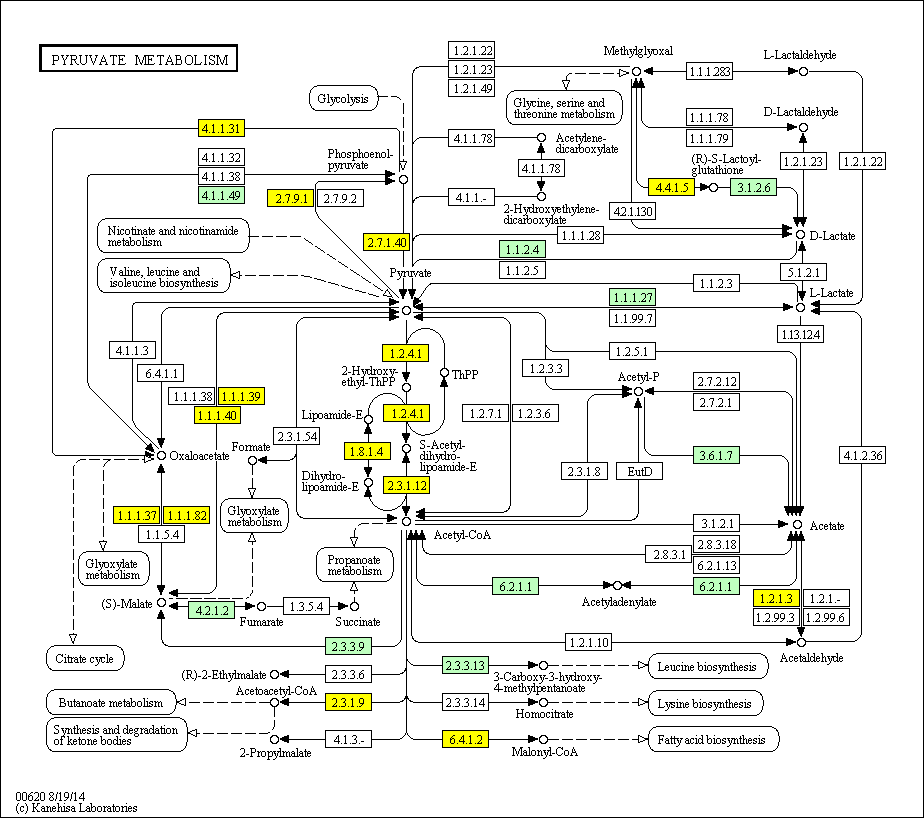

Supplement: Supplementary file 5 [file DataSheet1.ZIP › images/fve00620.png]

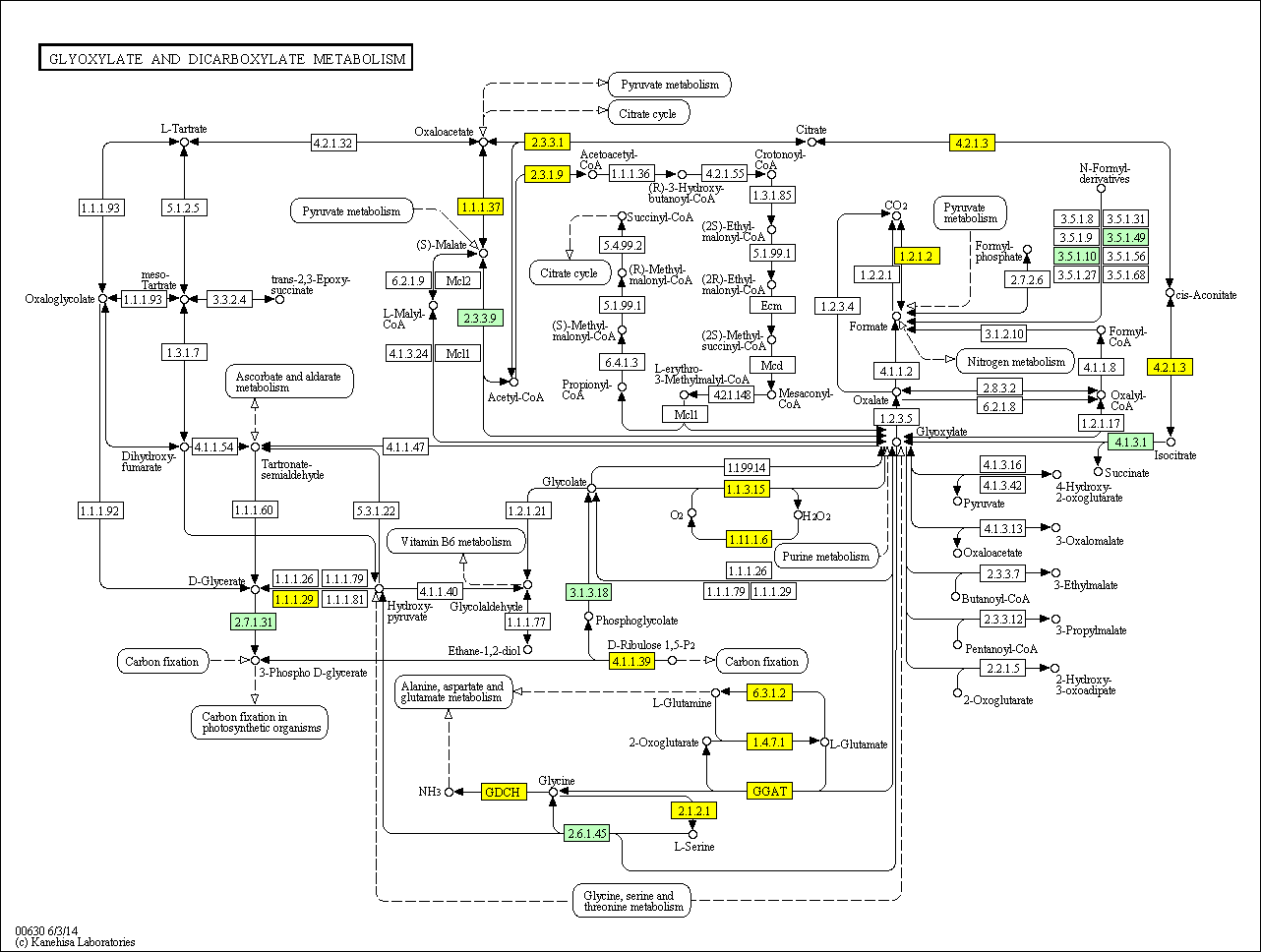

Supplement: Supplementary file 5 [file DataSheet1.ZIP › images/fve00630.png]

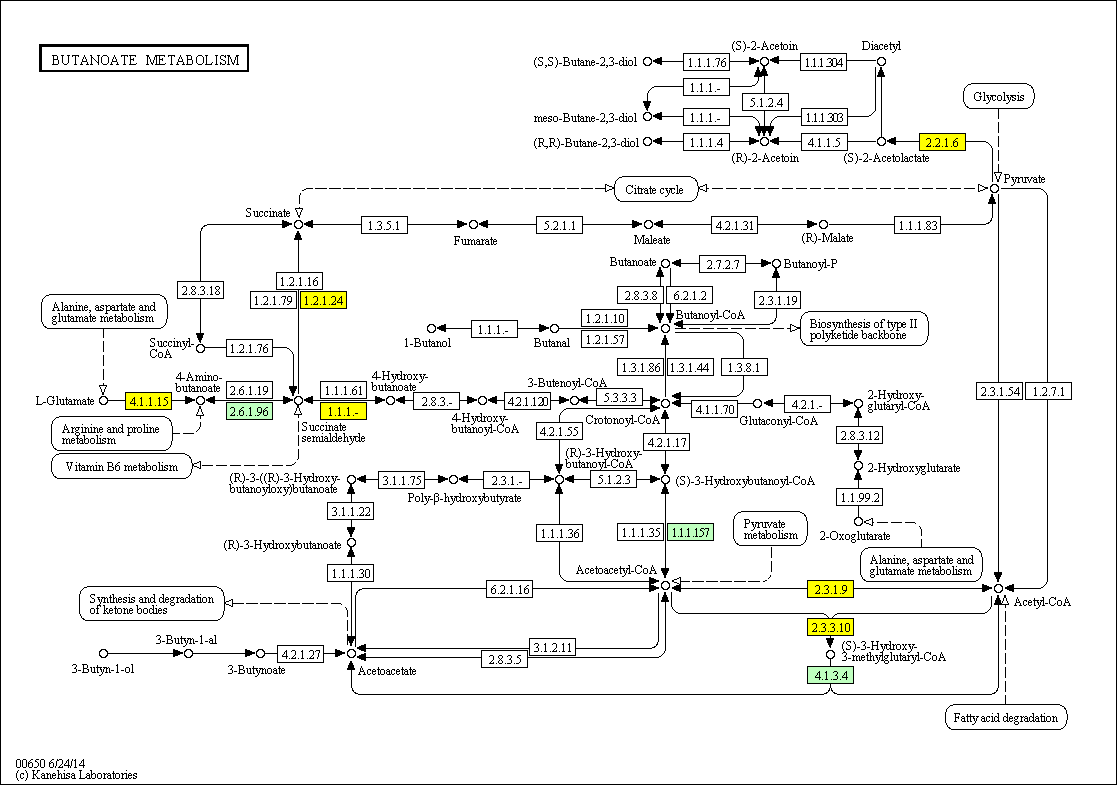

Supplement: Supplementary file 5 [file DataSheet1.ZIP › images/fve00650.png]

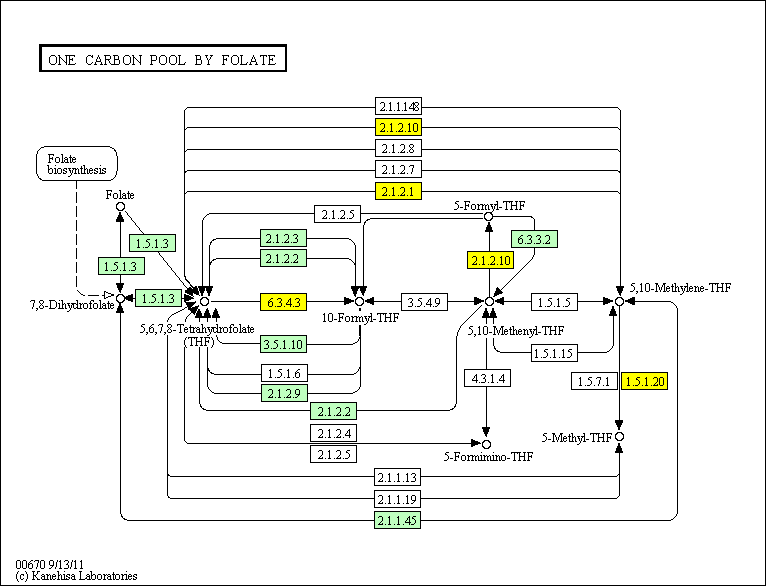

Supplement: Supplementary file 5 [file DataSheet1.ZIP › images/fve00670.png]

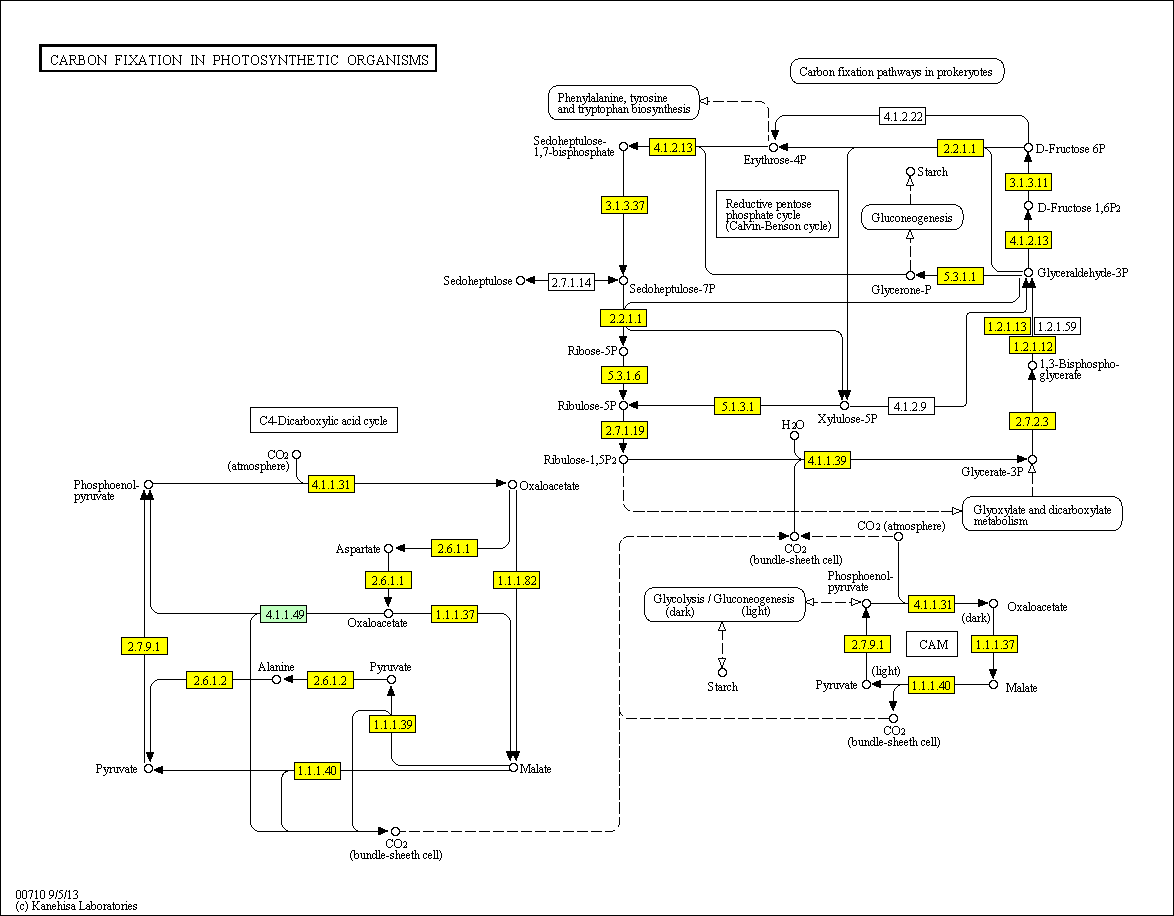

Supplement: Supplementary file 5 [file DataSheet1.ZIP › images/fve00710.png]

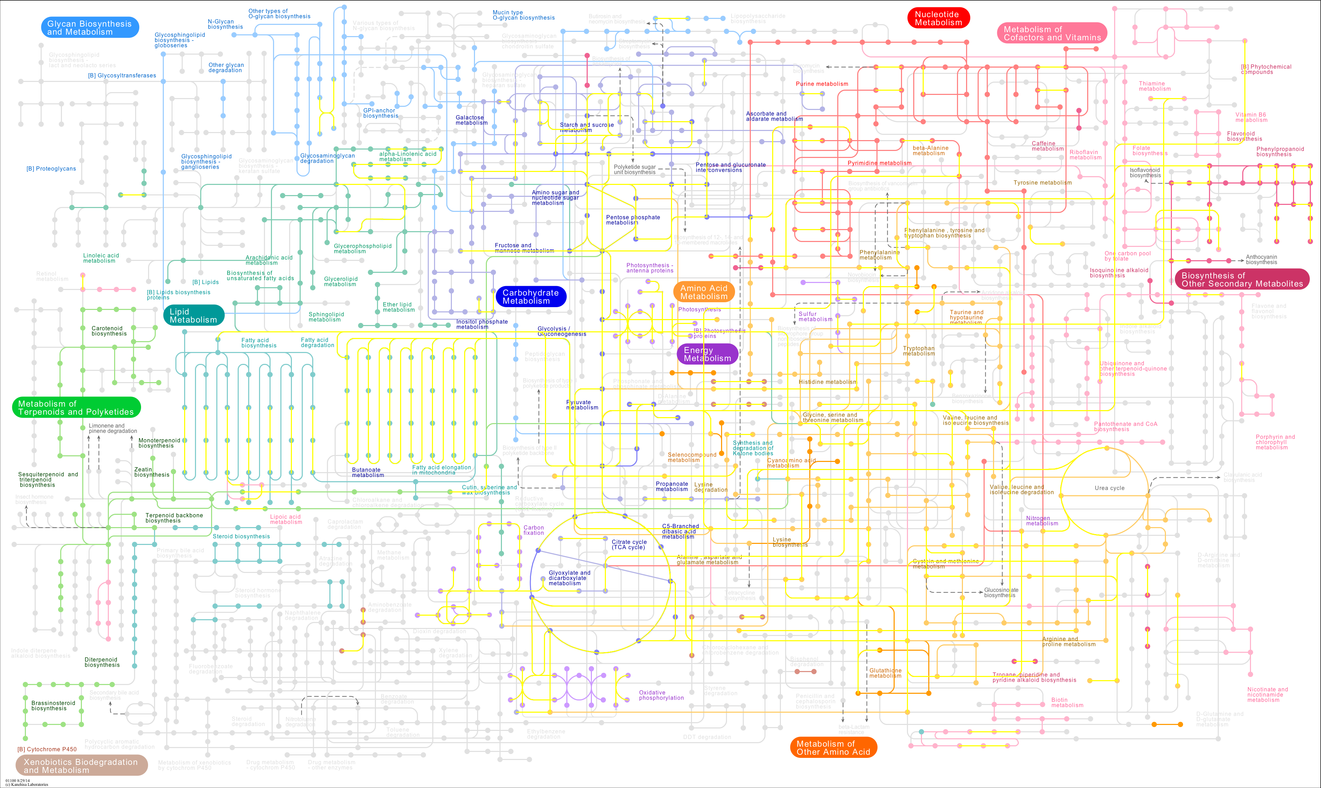

Supplement: Supplementary file 5 [file DataSheet1.ZIP › images/fve01100_0.3513941.png]

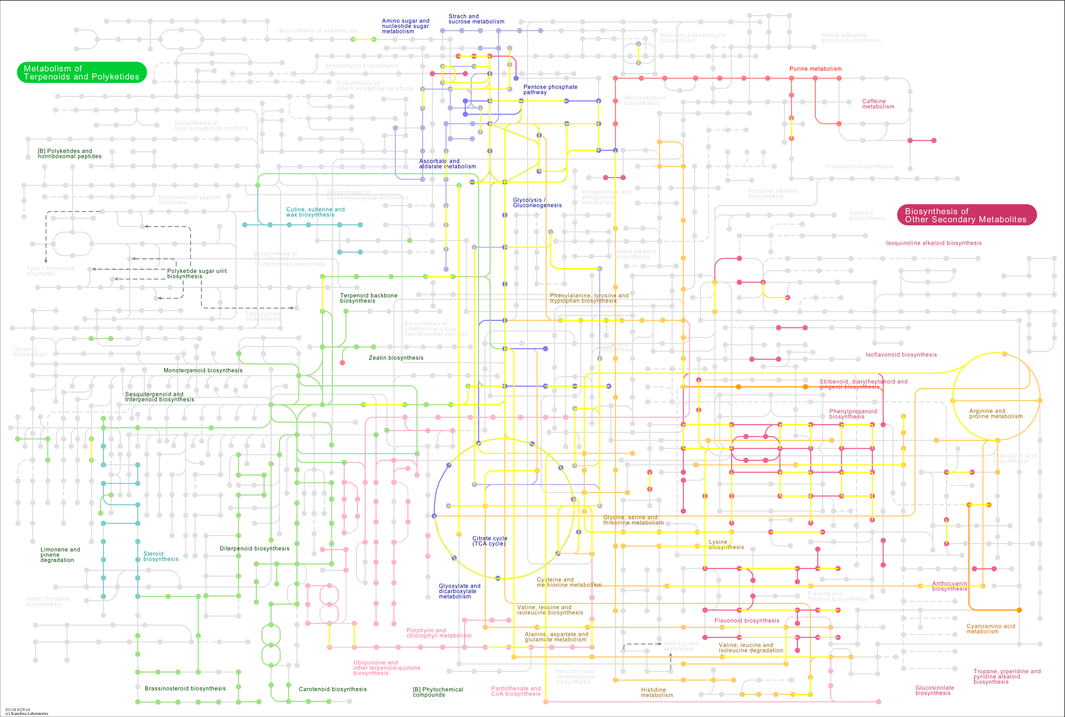

Supplement: Supplementary file 5 [file DataSheet1.ZIP › images/fve01110_0.3516600.png]

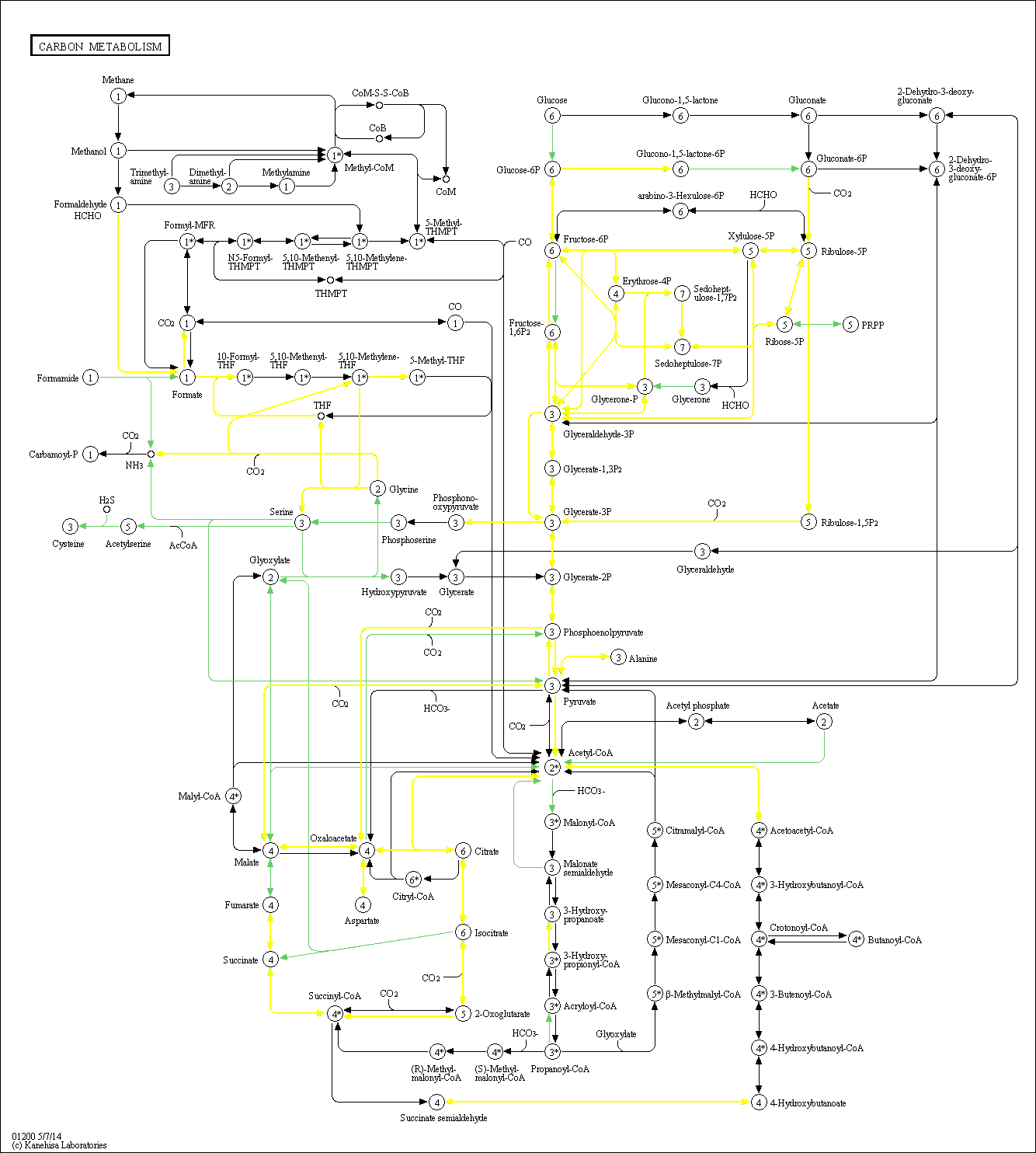

Supplement: Supplementary file 5 [file DataSheet1.ZIP › images/fve01200.png]

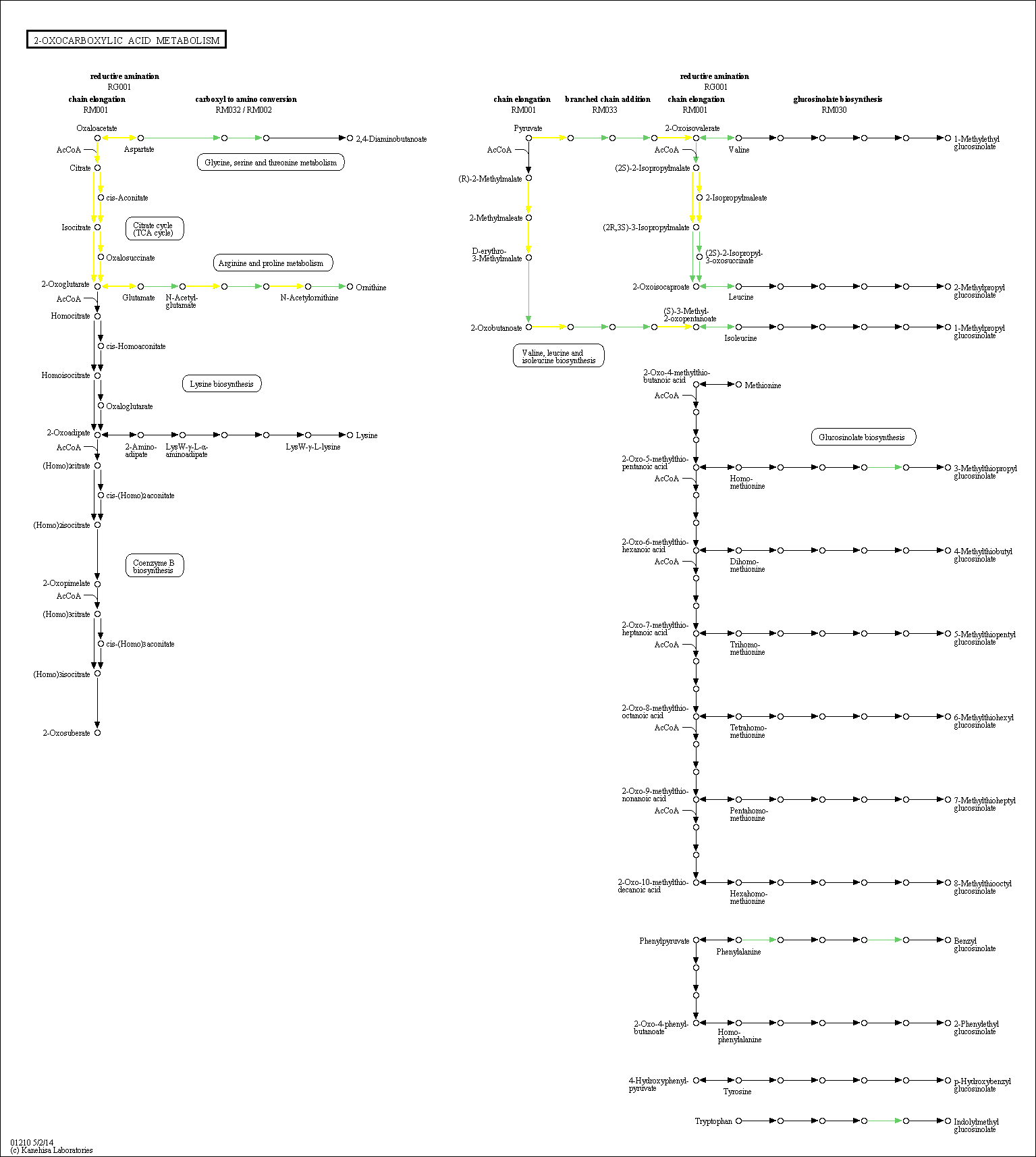

Supplement: Supplementary file 5 [file DataSheet1.ZIP › images/fve01210.png]

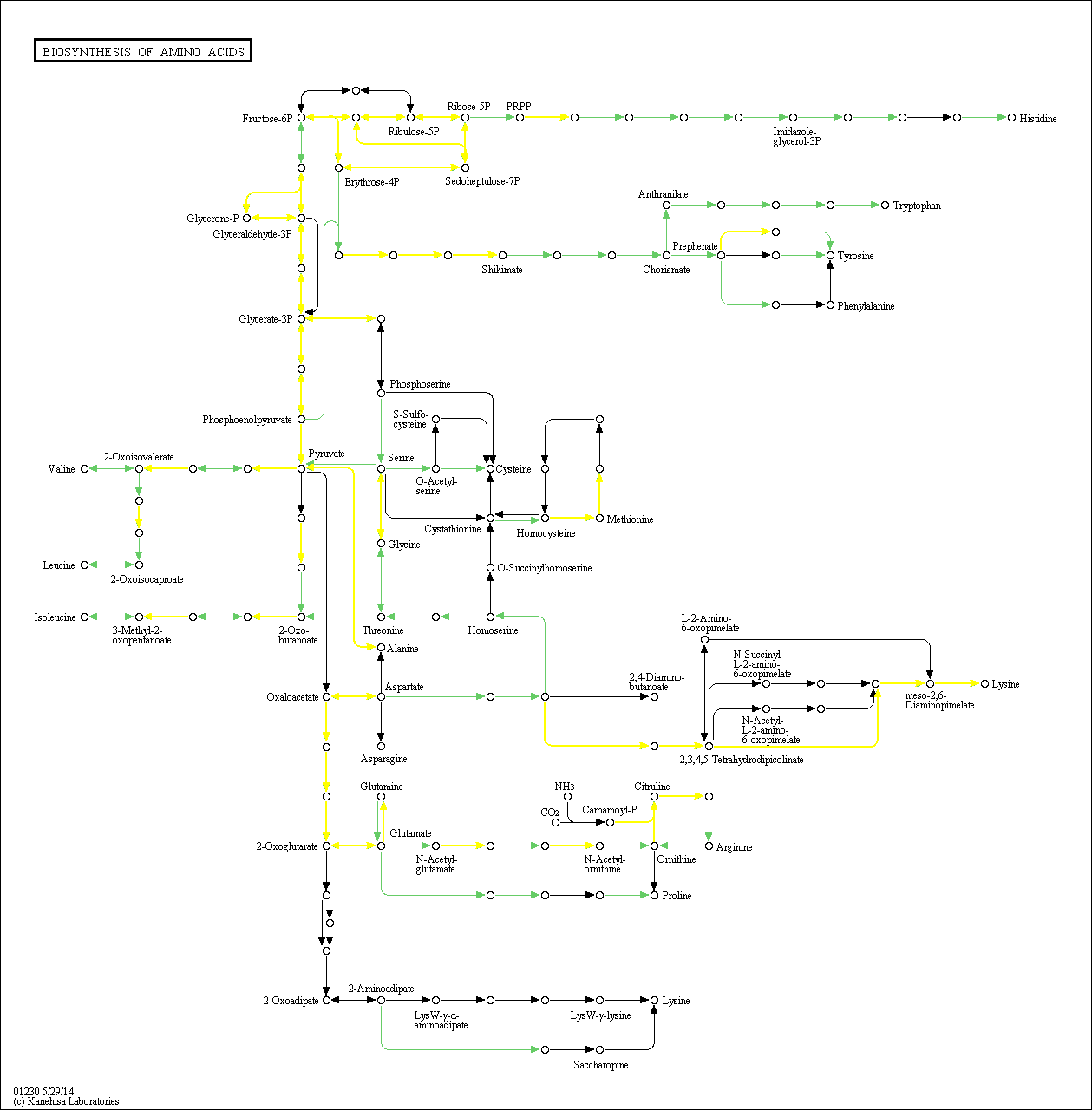

Supplement: Supplementary file 5 [file DataSheet1.ZIP › images/fve01230.png]

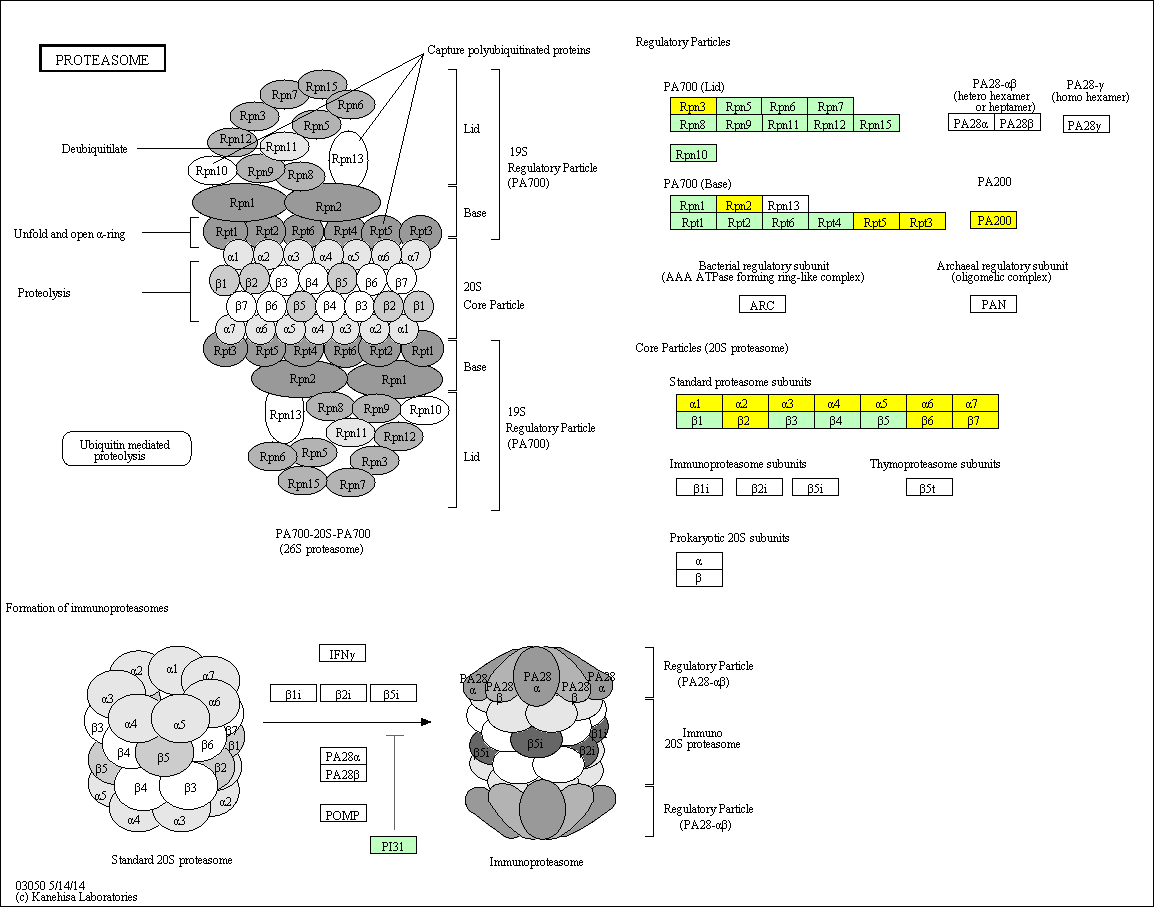

Supplement: Supplementary file 5 [file DataSheet1.ZIP › images/fve03050.png]

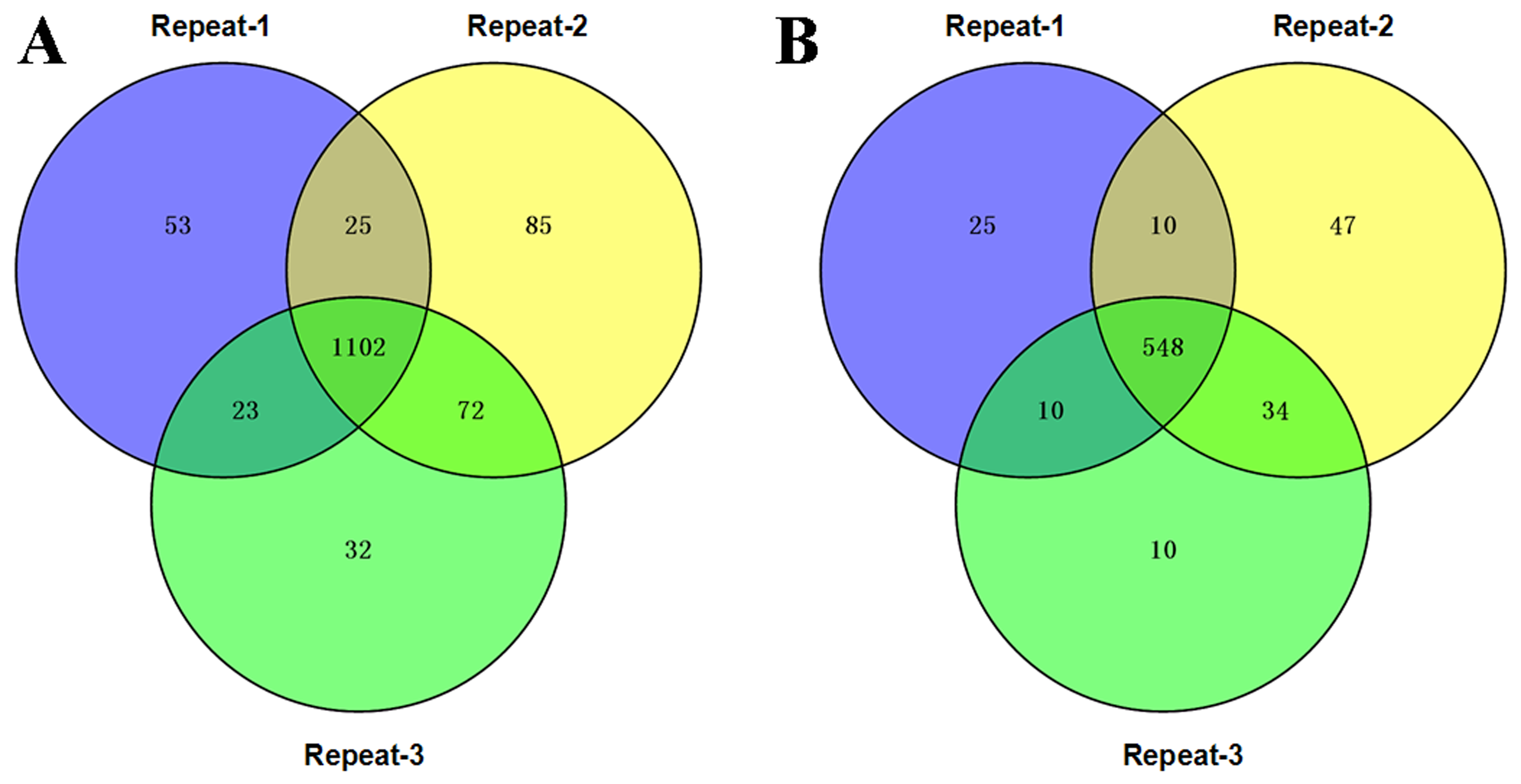

Supplement: Supplementary file 7 [file Image1.TIF]

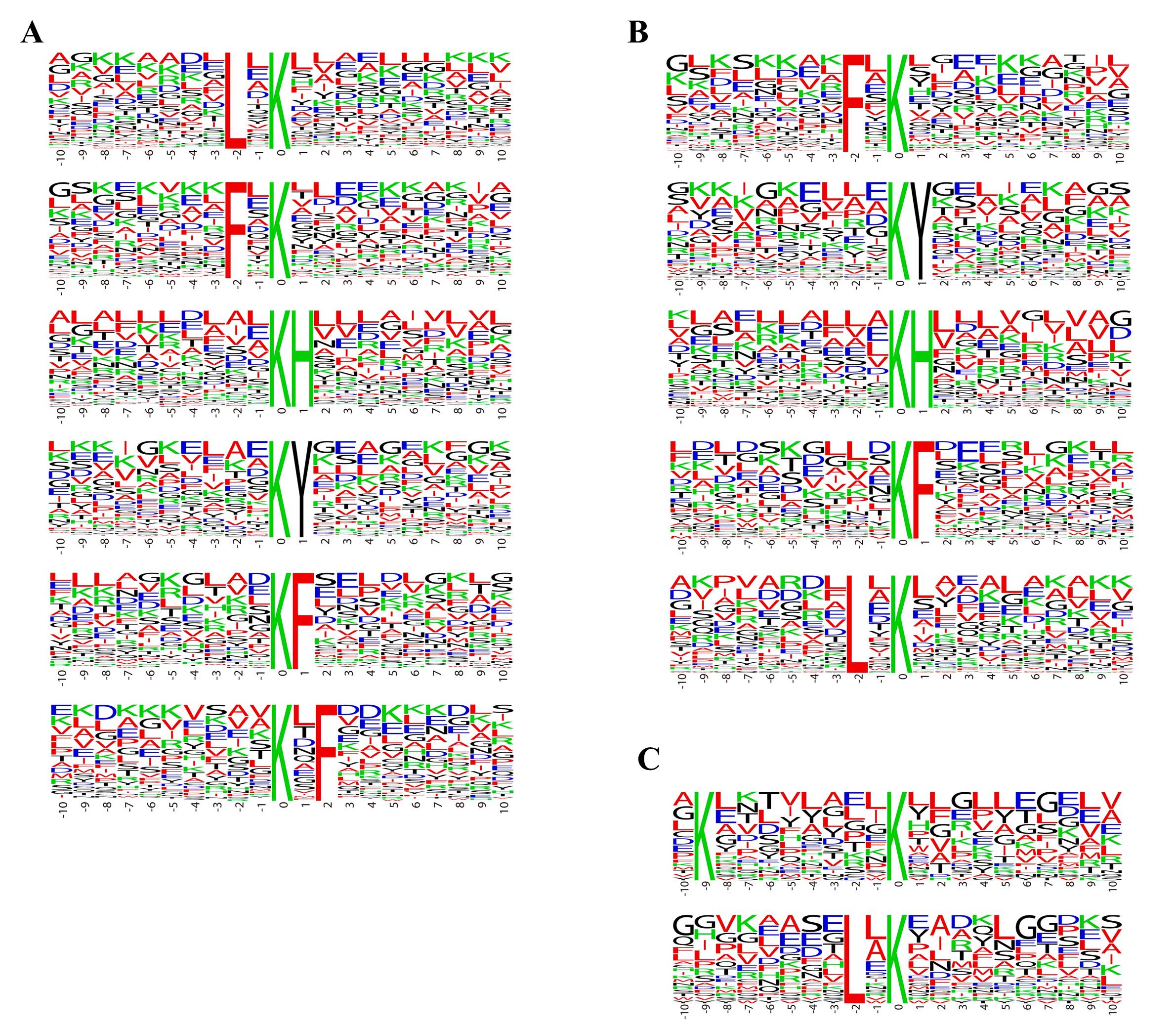

Supplement: Supplementary file 8 [file Image2.JPEG]
