# Supplementary material for: Global analysis of lysine acetylation in strawberry leaves
Source: Front Plant Sci. 2015 Sep 15;6:739. doi: 10.3389/fpls.2015.00739 (PMC4569977; doi:10.3389/fpls.2015.00739)
Supplement: Supplementary file 6 [file DataSheet2.DOCX]

**Supporting information**

**Supplementary Figure S1**. Venn diagram analysis of all the lysine acetylated peptides and proteins from three biological replicates. (A), Venn diagram analysis of all the lysine acetylated peptides (B) Venn diagram analysis of all the lysine acetylated proteins.
**Supplementary Figure S2**. Motif analysis of all the lysine acetylated peptides (A), chloroplast related peptides (B) and photosynthesis related peptides (C) in strawberry leaves.

**Supplementary Table 1** Summary of all the identified acetylated sites and proteins in strawberry leaves.

**Supplementary Table** **2** The degree, protein name and accession informations of the acetylated proteins in the protein-protein interaction network.

**Supplementary Table 3** The degree, protein name and accession information of the acetylated proteins in the significantly enriched subclusters.

**Supplementary Table 4** Summary of all the photosynthesis related acetylated peptides and proteins in strawberry leaves.

**Supplementary Package 1** The demonstration of all the significantly enriched KEGG pathways.


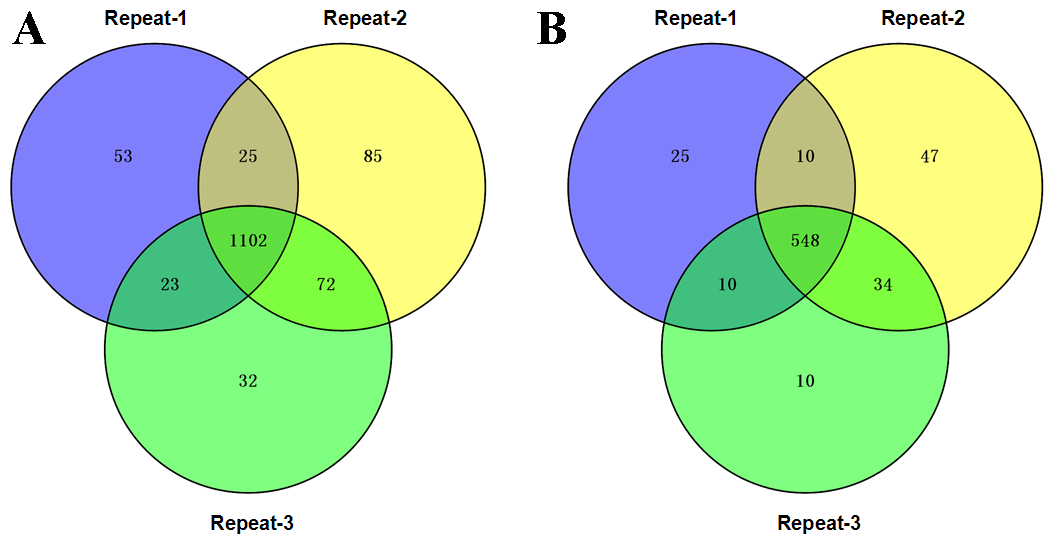


**Supplementary Figure S1**. Venn diagram analysis of all the lysine acetylated peptides and proteins from three biological replicates. (A), Venn diagram analysis of all the lysine acetylated peptides (B) Venn diagram analysis of all the lysine acetylated proteins.


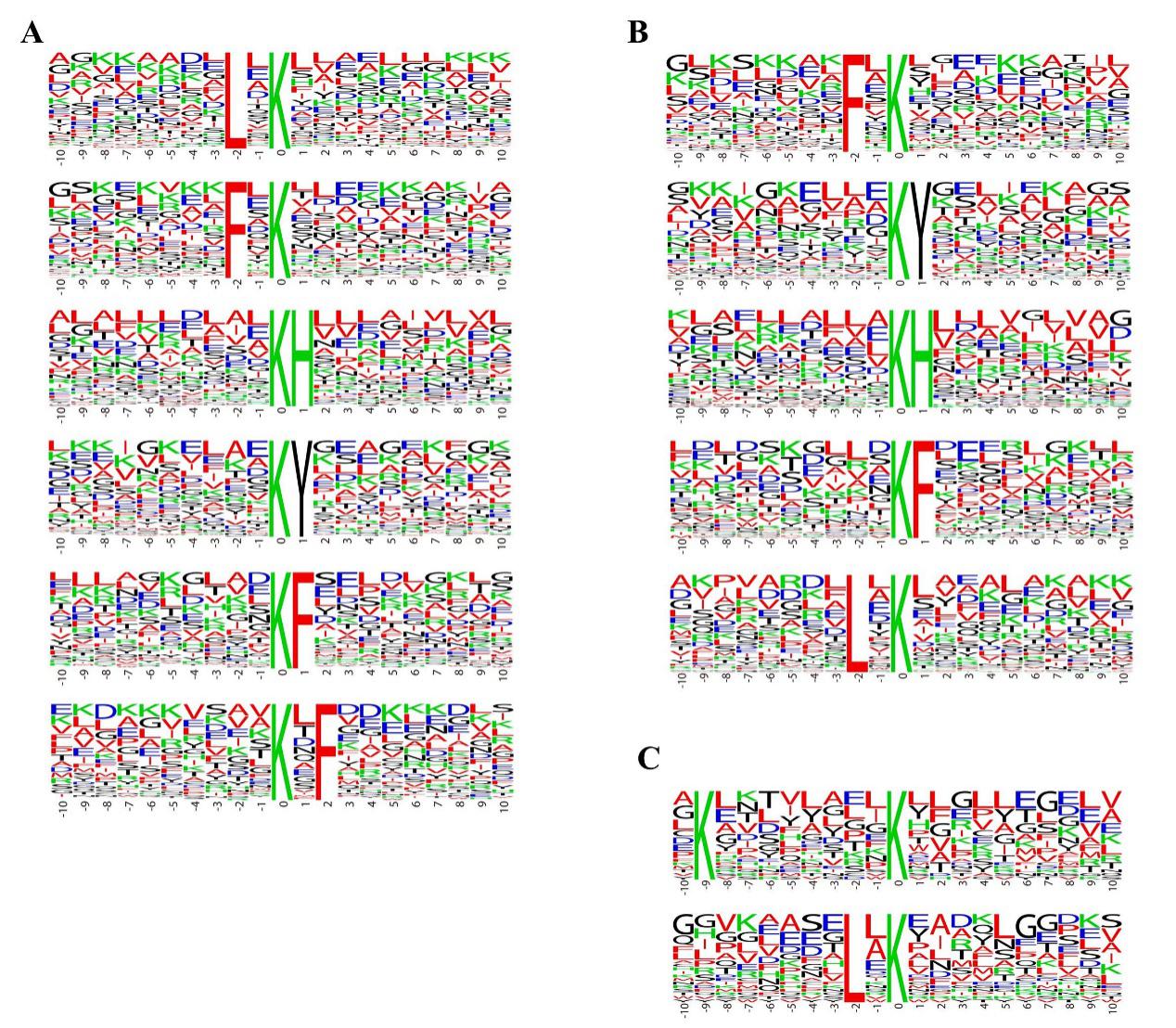


**Supplementary Figure S2**. Motif analysis of all the lysine acetylated peptides (A), chloroplast related peptides (B) and photosynthesis related peptides (C) in strawberry leaves.
